# Comparative Genomic Analysis of* Trichinella spiralis* Reveals Potential Mechanisms of Adaptive Evolution

**DOI:** 10.1155/2019/2948973

**Published:** 2019-05-21

**Authors:** Zigang Qu, Wenhui Li, Nianzhang Zhang, Li Li, Hongbin Yan, Tingting Li, Jianmin Cui, Yang Yang, Wanzhong Jia, Baoquan Fu

**Affiliations:** ^1^State Key Laboratory of Veterinary Etiological Biology, Key Laboratory of Veterinary Public Health of the Ministry of Agriculture, Key Laboratory of Veterinary Parasitology of Gansu Province, Lanzhou Veterinary Research Institute, Chinese Academy of Agricultural Sciences, Lanzhou 730046, China; ^2^Jiangsu Co-Innovation Center for Prevention and Control of Important Animal Infectious Disease, Yangzhou 225009, China

## Abstract

Trichinellosis caused by parasitic nematodes of the genus* Trichinella *may result in human morbidity and mortality worldwide. Deciphering processes that drive species diversity and adaptation are key to understanding parasitism and developing effective control strategies. Our goal was to identify genes that are under positive selection and possible mechanisms of adaptive evolution of* Trichinella spiralis *genes using a comparative genomic analysis with the genomes of* Brugia malayi*,* Trichuris suis*,* Ancylostoma ceylanicum, *and* Caenorhabditis elegans*. The CODEML program derived from the PAML package was used to deduce the most probable dN/dS ratio, a measurement to detect genes/proteins undergoing adaptation. For each pair of sequences, those with a dN/dS ratio > 1 were considered positively selected genes (PSGs). Altogether, 986 genes were positively selected (*p*-value < 0.01). Genes involved in metabolic pathways, signaling pathways, and cytosolic DNA-sensing pathways were significantly enriched among the PSGs. Several PSGs are associated with exploitation of the host: modification of the host's metabolism, creation of new parasite-specific morphological structures between* T. spiralis *and the host interface, xenobiotic metabolism to combat low oxygen concentrations and host toxicity, muscle cell transformation, cell cycle arrest, DNA repair processes during nurse cell formation, antiapoptotic factors, immunomodulation, and regulation of epigenetic processes. Some of the* T. spiralis *PSGs have* C. elegans *orthologs that confer severe or lethal RNAi phenotypes. Fifty-seven PSGs in* T. spiralis *were analyzed to encode differentially expressed proteins. The present study utilized an overall comparative genomic analysis to discover PSGs within* T. spiralis *and their relationships with biological function and organism fitness. This analysis adds to our understanding of the possible mechanism that contributes to* T. spiralis *parasitism and biological adaptation within the host, and thus these identified genes may be potential targets for drug and vaccine development.

## 1. Introduction

Trichinellosis is caused by nematodes of the genus* Trichinella*. These parasites have a worldwide distribution and include at least nine species, plus three additional recognized genotypes [1]. The genus has extensive biodiversity and is able to parasitize many species, including mammals, birds, and reptiles. Adult* T. spiralis *are parasites that live in the intestinal epithelium. In contrast, juvenile forms have an anaerobic metabolism and reside in nurse cells, which are host muscle cells. This occurs when* T. spiralis *newborn larvae penetrate individual muscle fibers and subvert and redirect host cell activities to promote their own survival. These parasites alter gene expression in the host cell from that of a contractile muscle fiber to that of a nurse cell, a cell that functions solely to nourish the worm. Changes occur after the nematode enters the host muscle cell; the fiber loses its myofilaments, its nuclei enlarge (hypertrophy), the amount of smooth endoplasmic reticulum increases, mitochondria degenerate, and eventually the entire nurse cell/parasite unit becomes encapsulated with collagen, which is secreted by the nurse cell. During the developmental phase from muscle larva to adult worm, parasite metabolism changes from anaerobic to aerobic [2]. Although these biological characteristics have been recognized for decades, the genetic basis for this adaptive metamorphosis of nurse cell formation and metabolic alterations associated with* T. spiralis *infection are still poorly understood.

Generally, parasites develop survival strategies for existence within the host including highly specific genetic adaptation. For* Trichinella*, conversion of host striated muscle cells to nurse cells is a crucial adaptation in the host that enables the parasite to reproduce successfully. For this particular adaptation to occur, the organism must have employed mechanisms to change gene function over time. Random genetic mutation may confer a selective advantage that can alter amino acids, and thus individual genes may acquire a function that has a positive effect on the survival of the parasite. An increased rate of nonsynonymous substitutions in rapidly evolving genes can also occur and this is assisted by directional selection of advantageous mutations. A systematic analysis of positive selection within the* T. spiralis *genome could provide perspective into the evolutionary and biological mechanisms that allow* T. spiralis *to occupy such a specific niche within the host. The positively selected protein-coding genes are recognized as a higher rate of nonsynonymous substitutions than synonymous substitutions. Mechanisms that are involved in a change of function depend on the occurrence of random mutations of preexisting genes, which enable a change to optimal fitness through a process of adaptation to the novel host environment. The search for genes that may have undergone positive selection for adaptive parasitism involves the detection of a significantly higher rate of amino acid replacement or nonsynonymous substitutions compared to synonymous substitutions. Large-scale systematic searches for genes under positive selection have defined direct links between selection and function in mammals, virus and bacteria [3–5].* In silico *screening for signals of positive selection in parasites becomes a practical feasibility because of the availability of genomes from other related parasites. The detection of genes under positive selection requires identification of orthologs by comparing several closely related species and then conducting positive selection scanning.

In the present study, we analyzed the genes that might be positively selected within the* T. spiralis* genome. Our goal was to explore the adaptive evolution of* T. spiralis* using a form of comparative genomic analysis with the genomes of* Brugia malayi*,* Trichuris suis*,* Ancylostoma ceylanicum,* and* Caenorhabditis elegans* using the dN/dS ratio as an indicator of positive selection. This analysis may serve as a foundation for future investigations to better understand parasitic adaptation at the molecular level and may also provide insights for holistic strategies to treat and control trichinellosis.

## 2. Materials and Methods

### 2.1. Genome Data

Genome data were downloaded from the corresponding websites as follows:

 
*A. ceylanicum *(ftp://ftp.wormbase.org/pub/wormbase/species/a_ceylanicum) [6]
 
*B. malayi* (ftp://ftp.wormbase.org/pub/wormbase/species/b_malayi) [7]
 
*C. elegans *(ftp://ftp.wormbase.org/pub/wormbase/species/c_elegans) [8]
 
*T. spiralis *(ftp://ftp.wormbase.org/pub/wormbase/species/t_spiralis) [9]
 
*T. suis *(ftp://ftp.wormbase.org/pub/wormbase/species/t_suis) [10]

### 2.2. Analysis of Positive Selection

One-to-one orthologs between the five nematodes were determined by applying the reciprocal BLAST best-hit means with an E-value cutoff of 1×10^−10^ and nucleotide sequence identity of more than 30% was selected.* T. spiralis*,* T. suis*,* B. malayi*,* A. caninum, *and* C. elegan *are phylogenetically distant members of the phylum Nematode. Besides, if its cutoff value was more stringent it would remove most of the orthologs. So a lower nucleotide identity was used.

Every orthologous gene set was compared by utilizing MUSCLE [11] and Gblocks was used for trimming [12]. All gaps and “N” within the alignments were deleted to decrease the effect of equivocal bases on the inference of positive selection. Finally, trimmed sequences of less than 150 bp (50 codons) were removed.

The dN/dS ratio is a trustworthy means for evaluating evolutionary pressures on protein-coding genes. In pairwise alignments of orthologous genes, the ratio of nonsynonymous distance (i.e., number of substitutions per nonsynonymous site; dN) over synonymous distance (dS) gives a general but conservative indication of the mode and strength of selection. Independent codon sites were simulated to produce data that could be analyzed by the PAML package [13]. The CODEML program from the PAML package was used to infer the most likely dN/dS ratio for each pair of sequences. The likelihood rate test (LRT) was used to detect significant positive selection on the foreground branch. The LRT was used to compare a model that allowed sites to be under positive selection in the foreground branch with the null model, in which sites could evolve neutrally and under purifying selection. Positively selected genes (PSGs) were inferred only if their* p-*values were less than 0.01. After identifying PSGs, the Bayes empirical Bayes (BEB) method was implemented to calculate posterior probabilities and to record positively selected sites.* p*-values of all PSGs also were normalized by controlling the false discovery rate using the Benjamini and Hochberg approach [14]. Genes with adjusted* p*-values < 0.01 showed statistically significant differences and were treated as candidates for positive selection. The gene name, functional annotations, protein orthologous classification, molecular interaction, and reaction networks of the PSGs were analyzed by BLAST, Gene Ontology (GO) (http://www.geneontology.org), and the Kyoto Encyclopedia of Genes and Genomes (KEGG) Pathway database (http://www.genome.jp/kegg), respectively.

### 2.3. Identification of PSGs That Encode Differentially Expressed Proteins

Differentially expressed proteins, identified by Liu et al. [15], were defined using isobaric tags for relative and absolute quantitation (iTRAQ) as those with at least a 1.5-fold change relative to one another, with p < 0.05. The above database of differentially expressed gene products was compared with the* T. spiralis *PSGs to reveal PSGs that are expressed specifically in different life stages.

### 2.4. Identification of T. spiralis PSGs with C. elegans Orthologs That Confer RNAi Phenotypes

In a previous report, 463* T. spiralis *genes were identified that have* C. elegans *orthologs that confer RNAi phenotypes (https://www.wormbase.org/) [16]. PSGs that overlapped with these 463* T. spiralis *genes with* C. elegans* RNAi orthologs were identified.

## 3. Results

### 3.1. Functional Classification of PSGs

**Figure 1:**
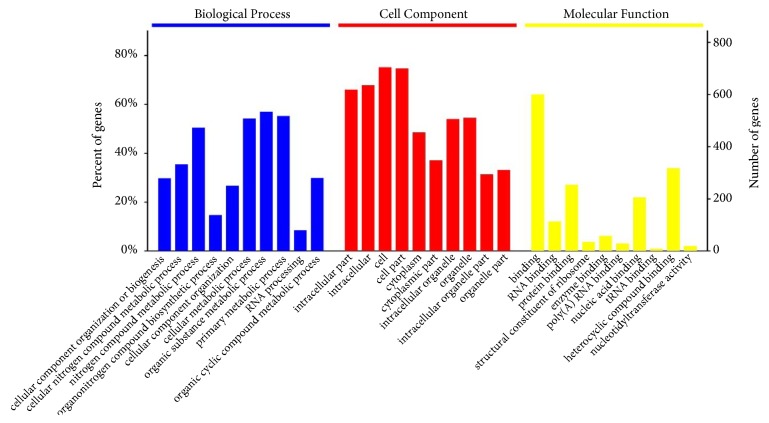
Gene Ontology (GO) term analysis for the positively selected genes of* T. spiralis*. The identified proteins were classified as GO terms in biological process, cellular component, and molecular function, according to their GO signatures. The left y-axis conveys the proportion of genes annotated in each GO term (gene number in each GO term divided by gene number in all GO terms). The right y-axis describes the gene number annotated in each GO term.

Nucleotide sequences with more than 30% identity were selected and a total of 1997 orthologs were obtained (See Supplementary Dataset 1). By using CODEML program to infer a dN/dS ratio for each pair of sequences, 986 genes were selected (*p*-value<0.01) (see Supplementary Dataset 2). PSGs were grouped into the GO categories of biological processes, cellular components, and molecular functions. GO analysis of the PSGs (see Supplementary Dataset 3) revealed that the PSGs encode proteins with a multiplicity of functions, including those with the following GO terms: binding, RNA binding, protein binding, structural constituent of ribosome, enzyme binding, poly (A) RNA binding, nucleic acid binding, tRNA binding, heterocyclic compound binding, and nucleotidyltransferase activity. Genes involved in cellular component organization or biogenesis, cellular nitrogen compound metabolic process, organonitrogen compound biosynthetic process, cellular metabolic process, organic substance metabolic process, primary metabolic process, RNA processing, and organic cyclic compound metabolic process were identified (Figure 1).

### 3.2. Pathway Enrichment of PSGs

**Figure 2:**
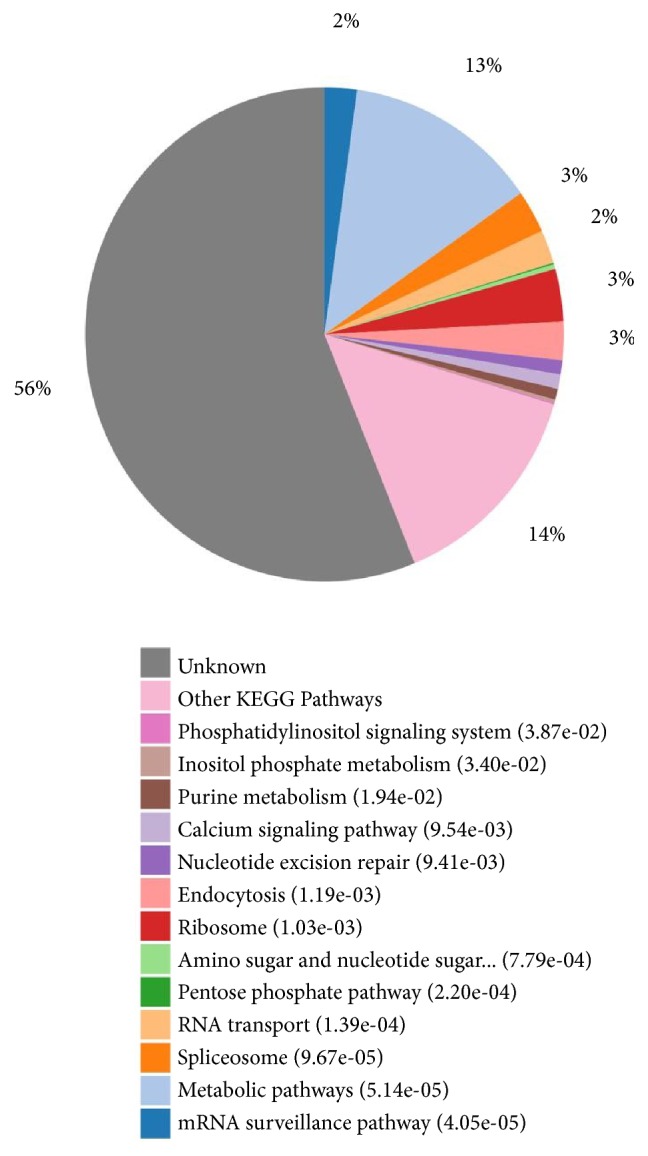
Pathway enrichment of positively selected genes in* T. spiralis* as defined by KEGG Pathway maps.

Molecular interaction and reaction networks of identified PSGs products were analyzed through KEGG pathway maps, which revealed that some PSGs could be ascribed to specific pathways, including metabolic pathways, the mRNA surveillance pathway, pentose phosphate pathway, amino sugar and nucleotide sugar, synthesis pathways, endocytosis, nucleotide excision repair, calcium signaling pathway, purine metabolism, inositol phosphate metabolism, and the phosphatidylinositol signaling system (Figure 2, see Supplementary Dataset 4 and Table 1).

Success parasitism of* T. spiralis *within host is likely to involve parasite adaptation of the host cells inhabited. During the nurse cell formation process, the mRNA surveillance pathway and nucleotide excision repair process are activated to adjust chromosome stability for adaptation to the host.* T. spiralis *inhabit intracellular niches during both the enteric and muscle phases of the infection; its metabolic pathways changed by decreasing its metabolic capacity to accommodate the lowered amount of nutrients within host within the niches of enteric and muscle phases to survive for substantial periods of time.

### 3.3. Analysis of Differentially Expressed PSGs

Over 1000 stage-specific proteins in* T. spiralis *have been identified previously [15, 17, 18]. In the current study, we examined the corresponding genes for the presence of PSGs and identified 57* T. spiralis *PSGs that are differentially expressed in different life-cycle stages (see Table 2).

The putative chitin binding peritrophin-A domain protein (GenBank No. EFV59360.1), angiotensin-converting enzyme, testis-specific isoform (GenBank No. EFV57539.1), putative IQ calmodulin-binding motif protein (GenBank No. EFV56231.1), and cuticle collagen 34 protein (GenBank No. EFV60533.1) were all differentially expressed in muscle larvae (ML) vs. newborn larvae (NBL), NBL vs. adult L3 larvae (Ad3), or ML vs. Ad3. Chitin binding peritrophin-A domain protein was involved in chitin binding which it may participate in cuticle formation [19]. The putative IQ calmodulin-binding motif protein, which binds calmodulin or calmodulin-like proteins, can interact with proteins that function in cell signaling, cytoskeletal reorganization, and cell differentiation; thus the putative IQ calmodulin-binding motif protein of* T. spiralis* may be also involved in regulating cell signaling, cytoskeletal reorganization and cell differentiation process, especially in regulating aspects of the infected muscle cell type. Several transporters differentially expressed in life-cycle were also PSGs, including a mitochondrial pyruvate carrier (brain protein 44), a putative proton-coupled amino acid transporter 4, the Y+L amino acid transporter 1, and excitatory amino acid transporter 1.

Other stage-specific PSGs identified include the G2/mitotic-specific cyclin-B3 and mitotic checkpoint protein BUB3, both of which regulate the cell cycle. Cell surface proteins, fructose-bisphosphate aldolase class-I, enolase, and putative ATP synthase, F1 delta subunit were also found. Actomyosin cytoskeletal organization proteins, GTP-binding ADP-ribosylation factor, actin-binding protein anillin, DNA replication and repair, transcription process components, transcriptional adapter 2-beta, histone acetyltransferases, and DNA topoisomerase were also differentially expressed PSGs.

### 3.4. Positively Selected Genes in T. spiralis Correspond to C. elegans Orthologs That Resulted in Severe RNAi Phenotypes

Previous research demonstrated that* T. spiralis *contains genes with* C. elegans *orthologs that produce severe or even lethal RNAi phenotypes. These* T. spiralis *genes were compared to the PSG list to determine if any of these orthologs have adaptive potential.  Table 3 shows a partial list of the identified PSGs in* T. spiralis*, focusing on those that conferred lethal or severe phenotypes in* C. elegans *(see Table 3).

Some of the PSGs that may confer lethal RNAi phenotypes were also differentially expressed. These included fatty acid synthase, a PDZ domain-containing protein, pyruvate dehydrogenase, zinc finger domain (C2H2 type) containing protein, GTP-binding ADP-ribosylation factor, and cyb-3 cyclin B like protein (G2/mitotic-specific cyclin-B3). The* C. elegans *orthologs produced severe RNAi phenotypes, including embryonic lethality, morphological body defects, and uncoordinated movement phenotypes.

## 4. Discussion

The evolution of parasitism is an example of the acquisition of complex traits that required multiple independent adaptations and changes in physiology, morphology, and life stage traits that involved many independent mutations in the genome. This study used a comparative genomics approach to identify PSGs of* T. spiralis *to help explain its unique adaptation to its host, characteristics such as adapting to low oxygen concentrations and tolerating toxicity within the host, and synthesis of new and unique* Trichinella*-specific morphological structures; PSGS may be one, along with other genetic mechanisms that explain the molecular adaptations for parasitism in* T. spiralis* [20].

Using the synonymous rate as a benchmark, one can determine whether fixation of nonsynonymous mutation is assisted or impeded by natural selection. If the nonsynonymous/synonymous rate ratio, *ω* = dN/dS, where dN > dS and *ω* > 1, the selection has an effect on fitness, and if nonsynonymous mutations are deleterious, purifying selection will reduce their fixation rate, so that dN < dS, *ω* < 1. A substantially higher nonsynonymous rate than synonymous rate is thus evidence for adaptive protein evolution [21]. Parasitic nematodes and protozoan use various strategies to adapt to the host environment, including positive selection at the genomic level, for example,* Toxoplasma gondii*,* Strongyloides papillosus*,* Leishmania *parasite, and* Plasmodium falciparum* [22–25].

*T. spiralis *and* T. suis *belong to Clade I,* B. malayi *belongs to Clade III,* C. elegan *and* A. caninum *belong to Clade V [2], and Clade II contains few vertebrate parasite that we do not choose. Clad I, Clade III, and Clade V members were chosen to study the adaptive evolution. Although their infection-routes are different,* B. malayi *is skin-penetrating,* A. caninum *is active-invasive,* T. spiralis* and* T. suis *are passive ingestion route, and* T. spiralis*,* T. suis*,* B. malayi, *and* A. caninum *have common features that they are all vertebrate parasite.* C. elegan *is a reasonable model system for other nematodes, although the percentage of identity between* C. elegan *and some parasitic nematodes can be quite low.

Parasitism is a generally recognized as living at the expense of a host. Existing species of nematodes reveal biological characteristics that are suggestive of an evolutionary pathway to parasitism [20]. Infection by* T. spiralis *immature L1 larvae initiates extensive reorganization of infected skeletal muscle cells, leading to reprogramming of a former terminally differentiated host cell to express a new phenotype, a nurse cell. During formation of the nurse cell, normal muscle nuclei are arrested in the G0/G1 state within the cell cycle progression, the phase at which gene expression within muscle is usually restricted [2]. Despommier proposed “parakines” as messengers to implement the communication between* T. spiralis *and host muscle cells by molecular cross-talking for the sake of providing permanent coexistence. It was hypothesized that the parakines direct specific cellular behavior by effecting signaling pathways [2]. In* T. spiralis*, several genes participate in signal transduction to regulate the cell cycle, including small G protein signaling modulator 3-like protein, G2/mitotic-specific cyclin-B3, mitotic checkpoint protein BUB3, cyclin-dependent kinase inhibitor 2B-related protein, and extracellular signal-regulated kinase 1; small G protein signaling modulator 3-like protein also participates in cell arrest. Extracellular signal-regulated kinase 1 is involved in the regulation of meiosis, mitosis, and postmitotic functions in differentiated cells. Cyclin-dependent kinase inhibitor 2B-related protein functions as a cell growth regulator that inhibits cell cycle G1 progression [26–28]. The G2/mitotic-specific cyclin-B3 and mitotic checkpoint protein BUB3 are checkpoint regulators in cell cycle [29].

The cell cycle is a strictly regulated and highly ordered process and several inherent checkpoints exist to ensure the high fidelity of cell replication. The G2/mitotic-specific cyclin-B3 and mitotic checkpoint protein BUB3 are checkpoint regulators that are positively selected by* T. spiralis *when compared in our analysis with other related nematodes. The G2/mitotic-specific cyclin-B3 has a severe lethal RNAi phenotype in its* C. elegans* ortholog [16], so the G2/mitotic-specific cyclin-B3 may be considered an essential gene in* T. spiralis*. Cyclins are positive regulatory subunits of cyclin-dependent kinases, which play an essential role in the control of the cell cycle, notably via their destruction during cell division [29]. The G2/mitotic-specific cyclin-B3 functions as a regulator of the G2/M transition in mitosis and may be important for events occurring in early meiotic prophase I; thus its rapid evolution would be needed to regulate the cyclin-dependent kinases, checkpoints of cell cycle [29]. Since nuclei of nurse cells undergo DNA synthesis, become 4N, and then stop in the G2/M phase [30], a fast evolving G2/mitotic-specific cyclin-B3 may contribute to the loss of the restricted control over cell division, resulting in a cell cycle halted at the G2/M phase. The mitotic checkpoint protein BUB3 has a role in mitotic spindle assembling checkpoint signaling to regulate the cell cycle arrest and also functions in oocyst meiosis as a regulator of chromosomal segregation [31]. The mitotic checkpoint protein BUB3 was positively selected in* T. spiralis*, suggesting that* T. spiralis *has a considerably higher pressure to maintain the high-fidelity chromosomal segregation than that of the other related nematodes, we examined, perhaps because of the nurse cell complex formation process. All signals are required in nurse cell complex formation and possibly and positively selected because of this unique adaptation of* Trichinella*.

The nurse cell is a unique site that supports maturation of* Trichinella *larvae, supplying nutrients for development, and* T. spiralis *undergoes metamorphosis in response to the drastically different host locations it occupies during its life-cycle. Once the collagen capsule forms around the nurse cell, the availability of substrates is restricted, so it would logically follow that* T. spiralis *encounters oxidative stress and xenobiotics. Physiologically,* T. spiralis *has adapted to low oxygen concentrations and tolerates toxicity within its environment. Thus, the rapidly evolving genes of the* T. spiralis *redox system may be a mechanism the parasite adapted to protect itself against the effects of reactive oxygen species. After* T. spiralis *forms the nurse cell, it is believed that* T. spiralis *adapts to its host by decreasing its metabolic capacity to accommodate the lowered amount of nutrients within host. It has been suggested that hosts utilize oxidative stress to defend against parasites [15, 32] and it follows that parasites, thus, would have sophisticated redox systems, which participate in the decomposition, detoxification, and biosynthesis of various compounds to combat reactive oxygen species [33]. Accordingly,* T. spiralis *may use the same strategies to adapt to its specific niche of creating a nurse cell from host tissue. In the current study oxidoreductase, members of the short chain dehydrogenase/reductase family, dimethylaniline monooxygenase [N-oxide-forming] 4, cytochrome P450 4V2, heparan sulfate glucosamine 3-O-sulfotransferase 3A1, protein-tyrosine sulfotransferase A, a putative ABC transporter, and an ATP-binding protein were positively selected. These genes encode detoxification enzymes and proteins involved in intracellular transport and may be used for host adaptation and ecology and may confer to* T. spiralis *the ability to adapt to low oxygen concentrations and tolerate some level of toxicity within the host. During the cyst formation process, there is increased metabolism of proteins, glucose, and lipids within the nurse cells. The blood supply of the* Trichinella *larva also increases to meet the demands of the elevated metabolic processes, resulting in angiogenesis in the area surrounding the cyst [34]. Another gene identified in this study, SPARC, known to be upregulated in angiogenesis [35], may be associated with angiogenesis around the cyst. After* T. spiralis *forms the nurse cell, it is believed that* T. spiralis *adapts to its host by decreasing its metabolic capacity to accommodate the lowered amount of nutrients within the host. In the current study, genes involved in modifying metabolic pathways were positively selected, including genes associated with *β*-oxidation, glycolysis, and phospholipid metabolism. Conceivably, these PSGs may participate in the accelerated metabolism within the nurse cell. For example, enolase functions as an important enzyme in glycolytic pathway and in* Trichinella *can promote newborn larval migration and invasion of host tissues [36]. We hypothesize that enolase may be a rapidly evolving gene because it may be required for the unique energy requirements in the nurse cell. L-lactate dehydrogenase, involved in anaerobic metabolism, was significantly upregulated in mature larvae as compared to the adult and newborn larval stages, which confirms that anaerobic metabolism is activated in the ML stage [15]. In the current study, L-lactate dehydrogenase was not only differentially expressed, but was also positively selected, suggesting that L-lactate dehydrogenase was required for rapid adaptation to anaerobic conditions. Fatty acid synthase and pyruvate dehydrogenase were essential for* C. elegans *survival; thus it can be speculated that fatty acid synthase and pyruvate dehydrogenase play the same in* T. spiralis*. Both fatty acid synthase and pyruvate dehydrogenase were differentially expressed and positively selected, suggesting that the fatty acid and acetyl-CoA metabolic processes for material and energy requirements in the switched nurse cell complex and intestinal epithelial cells required rapid adaptation.

During the metamorphosis of the host muscle cell into the nurse cell, several proteins are involved in structural changes, including chromatin structure changes and actin remodeling. ADP-ribosylation factors (ARFs) can function as regulators of vesicular traffic and actin remodeling [37]. GTP-binding ADP-ribosylation factor functions as regulators of vesicular traffic and actin remodeling, and RNAi experiments revealed that its absence can be lethal; thus, its rapid evolution can accelerate actin remodeling to form the nurse cell complex [38]. Actin-binding protein anillin plays a role in actomyosin cytoskeletal organization. Transcriptional adapter 2-beta participates in chromatin remodeling. Histone acetyltransferases and DNA topoisomerase can alter DNA replication, DNA repair, and transcription [39], so positive selection of these genes permits chromatin and actin structure changes that would facilitate the survival of* T. spiralis *within the host. In addition to genome encoded factors, epigenetic factors might participate in the transformation of* T. spiralis *into different life stages via temporally regulated gene expression. DNA methylation was present in* T. spiralis* and [40, 41] expression of some genes that encode proteases and other proteins with possible roles in penetration were regulated by methylation, suggesting that changes in DNA methylation might play a vital role in the transition of muscle cells to nurse cells. Furthermore, DNA-binding proteins [42] are frequently influenced by alterations in methylation and as a result are thought to inhibit host cell signaling thereby facilitating* T. spiralis *transformation to different life stages. In the current study, we found that a dnmt1 (de novo methyltransferase 1) homolog (GenBank No. EFV58204) was positively selected. Although dnmt1 is part of the maintenance methylation machinery, the* T. spiralis *homolog was identified as rapidly evolving and as such may regulate parasitism-related genes via DNA methylation, again lending support to the potential biological significance of epigenetics in* T. spiralis *parasitism, especially in the nurse cell formation process.

During the metamorphosis of the host muscle cell into the nurse cell, nuclear antigens are in close association with chromatin in the infected muscle, and these antigens may function as regulating infected muscle cell phenotype [16, 43]. In the current research, a proliferating cell nuclear antigen (GenBank No. EFV60368.1) was positively selected; thus it may be involved in the formation of nurse cell complex phenotype. Among the PSGs that are differentially expressed in the life-cycle, Leucine-rich repeat containing protein, fibronectin type-III domain-containing protein, putative fibronectin III domain-containing protein, and putative cadherin domain-containing protein are all proteins that function in cell adhesion [44], and fibronectin type-III domain-containing protein is involved in cell differentiation, facilitating nurse cell complex formation. Some MyoD-like, helix-loop-helix-like, DNA-binding FYVE finger domain, and required cell differentiation 1-like proteins homologs have been found in* T. spiralis *excretory-secretory fractions, implicating these proteins in the dedifferentiation/differentiation processes that occur in the muscle cell to nurse cell transformation [34]. In the current study, putative helix-loop-helix DNA-binding domain proteins and cell differentiation protein, RCD1-like protein, were positively selected, providing additional support to the notion that these parasite gene products are involved in the dedifferentiation/differentiation process. Furthermore, several genes function in DNA repair were positively selected, suggesting that thus these rapidly evolving genes may generate an elevated rate of nucleotide mismatches during nurse cell formation.

The parasite surface is a critical interface with the host immune system [45]. Parasitic helminths have evolved a series of surface-modification strategies, such as encystment (for example, nurse cell formation in* T. spiralis*) that allows the parasite to survive for several years within the host [2]. Cuticle proteins and excretory-secretory products that are incorporated in the cuticle together form an array of surface proteins that function to evade host immunity, to nurture development of the parasite, and promote parasite penetration [46]. In addition the cuticular proteins directly interface with the host immune system. At the molecular level the genes utilized are variable depending on the means of encapsulation; however, nematodes typically use surface proteins, such as tetraspanins, collagen, apomucin, and cadherins, often organized to create new structures with parasite-specific features [46]. Previous research revealed that, in the nematode,* Haemonchus contortus*, collagen and other cuticular proteins may be utilized in a cuticle remodeling process during the transition from its free-living to parasitic life stage [47, 48]. It is possible that this is a “universal” principal for adaptive evolution of parasites, and if so,* T. spiralis *may use the same strategy. During* T. spiralis *cuticular encystment and encapsulation, the cuticle of the parasite is modified, an external layer secreted, which stimulates the host to encapsulate the worm. Little is known about the molecular mechanisms of this process. The PSGs of* T. spiralis* contain several cuticle proteins and one, collagen 34 protein, was differentially expressed. Several other cuticular collagens and putative cadherin domain proteins were positively selected. We suggest that the PSGs in the collagen and cadherin families may be involved in structural remodeling during the nurse cell formation process, suggesting that these genes merit further investigation.

Proteases and peptidases are necessary for penetration, digestion, or modification of host tissue and thus have important roles in parasitism [49]. The positively selected protease genes identified here may have roles in host muscle penetration by* T. spiralis *and its adaptation in this specific location in the host. Serpins participate in modulating host immune responses, fibrinolysis, coagulation, and inflammation [50]. In our study, a serpin (GenBank No. EFV57375.1) was positively selected; thus it can be speculated that, during the adaptive process, serpin serves to modulate the immune response at the host-parasite interface.

In previous research, birth/death, duplication/deletion, and domain shuffling events among protein families and domains were explored to interpret* T. spiralis *evolution [20, 51]. Their work demonstrated that loss of protein families involved in the DNA catabolic process and in DNA repair process resulted in increased chromosome instability [20]. In the current study, several genes that were identified as part of the DNA repair process and chromatin remolding process were positively selected, suggesting that positive selection may adjust chromosome stability for adaptation to the host.

The unique formation of nurse cell complex involves morphological and physiological alterations of* T. spiralis *and its host. With the available genome data, adaptation and parasitism can be studied at the genomic level [20, 52]. This study, using one form of comparative genomics, indicates that* T. spiralis *may have evolved various mechanisms, through positive selection, to adapt to its unique and specific niche in the host. By comparing the genome sequences of* T. spiralis*, to other nematode genomes, specific genes within the* T. spiralis *genome were identified that are under positive selection, suggesting roles in the formation of the unique nurse cell complex and overall biological adaptation of this parasite. The pitfall of this study is that genomics does not indicate whether a gene is actually expressed in the parasite. Thus, the follow-up biochemical analysis of the parasite would, in the future, be further investigated. Ultimately, these rapidly evolving genes and gene products, used by* T. spiralis *to adapt to its unique environment in the host, may be targets for treatment and preventative measures against this diverse and widespread parasite.

## Figures and Tables

**Table 1:** Gene functions for some of the positively selected genes identified in *T. spiralis*.

Gene function	GenBank No.	Gene family
*Xenobiotic metabolism*	EFV51745	oxidoreductase, short chain dehydrogenase/reductase family
	EFV54917	dimethylaniline monooxygenase [N-oxide-forming] 4
	EFV50316	cytochrome P450 4V2
	EFV50200	heparan sulfate glucosamine 3-O-sulfotransferase 3A1
	EFV57943	protein-tyrosine sulfotransferase A
	EFV50642	putative ABC transporter, ATP-binding protein
	EFV53848	putative ABC transporter, ATP-binding protein
	EFV54367	putative ABC transporter, ATP-binding protein
	EFV56075	putative ABC transporter, ATP-binding protein
	EFV48926	putative ABC transporter, ATP-binding protein, partial

*DNA repair proteins*	EFV4940	DNA mismatch repair protein Msh6
	EFV57314	DNA repair protein RAD51
	EFV55613	DNA repair protein Rad4
	EFV55989	DNA cross-link repair 1A protein
	EFV62248	DNA excision repair protein haywire

*Penetration/digestion*	EFV60084	lysosomal aspartic protease
	EFV52155	ATP-dependent Clp protease, proteolytic subunit ClpP
	EFV51535	putative ATP-dependent Clp protease, ATP-binding subunit ClpX
	EFV53966	cysteine protease ATG4B
	EFV54564	putative ATP-dependent protease La

*Muscle cell transformation*	EFV53122	putative helix-loop-helix DNA-binding domain protein
	EFV57695	putative helix-loop-helix DNA-binding domain protein
	EFV61981	putative helix-loop-helix DNA-binding domain protein
	EFV55197	cell differentiation protein RCD1-like protein

*Cell cycle arrest*	EFV56859	G2/mitotic-specific cyclin-B3
	EFV56912	cyclin-dependent kinase inhibitor 2B-related protein

*Antiapoptotic and immunomodulatory activities*	EFV60199	translationally-controlled tumor protein-like protein

*Surface proteins used for creating new parasite-specific morphological structures*		

Cadherin	EFV54837	putative cadherin domain protein, partial
	EFV54875	putative cadherin domain protein, partial
	EFV57941	putative cadherin domain protein

Cuticle collagen	EFV60533	cuticle collagen 34 protein
	EFV60602	cuticle collagen 3
	EFV52694	cuticle collagen rol-6
	EFV52706	cuticle collagen dpy-7
	EFV52845	cuticle collagen dpy-13

*Metabolic Pathways*		

*β*-oxidation pathway	EFV50357	putative 3-hydroxyacyl-CoA dehydrogenase, NAD binding domain protein
	EFV58588	lonCoA ligase 5

Glycolysis	EFV55075	putative hexokinase HKDC1
	EFV52822	enolase
	EFV54356	L-lactate dehydrogenase

Phospholipid metabolism	EFV56439	phospholipase, patatin family
	EFV60905	phospholipase D3 protein

*Epigenetic process regulatory machinery*	EFV58204	putative CXXC zinc finger domain protein

**Table 2:** *T. spiralis* PSGs encoding differentially expressed proteins in adults, muscle larvae and newborn larvae stages.

Accession No.	Description	Ratio	Ratio	Ratio
		ML/Ad3	ML/NBL	Ad3/NBL
E5S768/EFV59360.1	putative chitin binding peritrophin-A domain protein	2.26	0.54	2.89

E5SCG4/EFV57539.1	angiotensin-converting enzyme, testis-specific isoform	1.85	2.75	0.36

E5SG15/EFV56231.1	putative IQ calmodulin-binding motif protein	0.62	2.12	0.55

E5SJW0/EFV54917.1	dimethylaniline monooxygenase [N-oxide-forming] 4	-* *-* *-	2.01	-* *-* *-

E5SCL8/EFV57458.1	gamma-glutamyltranspeptidase 1	-* *-* *-	2.58	-* *-* *-

E5SHM8/EFV55721.1	diacylglycerol O-acyltransferase 2	1.74	-* *-* *-	-* *-* *-

E5SEH3/EFV56859.1	G2/mitotic-specific cyclin-B3	-* *-* *-	-* *-* *-	2.26

E5S186/EFV61456.1	leucine-rich repeat and fibronectin type-III domain-containing protein 4	-* *-* *-	3.06	-* *-* *-

E5S732/EFV59418.1	Putative fibronectin type III domain protein	-* *-* *-	2.60	-* *-* *-

E5SK45/EFV54875.1	putative cadherin domain protein, partial	-* *-* *-	3.06	-* *-* *-

E5S9G6/EFV58492	cystathionine beta-synthase	2.14	0.56	-* *-* *-

E5S621/EFV59764.1	mitochondrial pyruvate carrier (brain protein 44)	-* *-* *-	-* *-* *-	1.75

E5S718/EFV59404.1	putative proton-coupled amino acid transporter 4	2.11	-* *-* *-	-* *-* *-

E5RYX1/EFV62291.1	Y+L amino acid transporter 1	-* *-* *-	2.51	-* *-* *-

E5SFC3/EFV56520.1	excitatory amino acid transporter 1	-* *-* *-	2.25	-* *-* *-

E5S9E0/EFV58588.1	lonCoA ligase 5	1.60	0.52	-* *-* *-

E5SGN7/EFV56123.1	fatty acid synthase	1.69	-* *-* *-	1.76

E5S673/EFV59699.1	GDP-L-fucose synthetase	2.20	-* *-* *-	1.88

E5RYD9/EFV62441.1	phosphoglucomutase-2	2.29	-* *-* *-	-* *-* *-

E5SGM8/EFV56114.1	phosphomevalonate kinase	1.62	-* *-* *-	1.94

E5SJ10/EFV55223.1	calcium/calmodulin-dependent 3′,5′-cyclic nucleotide phosphodiesterase1C	-* *-* *-	3.93	-* *-* *-

E5S2V7/EFV60868.1	conserved hypothetical protein	1.90	-* *-* *-	1.93

E5SEI1/EFV56867.1	conserved hypothetical protein	-* *-* *-	2.41	-* *-* *-

E5S2J6/EFV60967.1	hypothetical protein Tsp_03059	2.81	2.22	-* *-* *-

E5S923 /EFV58705.1	small G protein signaling modulator 3-like protein	1.63	-* *-* *-	-* *-* *-

E5SFG5/EFV56461.1	activating signal cointegrator 1	-* *-* *-	2.08	-* *-* *-

E5SBW7/EFV57723.1	extracellular signal-regulated kinase 1	-* *-* *-	-* *-* *-	1.91

E5SIP9/EFV55337.1	transcriptional adapter 2-beta	1.64	-* *-* *-	1.98

E5SIM1/ EFV55358.1	putative BTB/POZ domain protein	2.08	-* *-* *-	-* *-* *-

E5S9P2 /EFV58568.1	putative PDZ domain protein	1.77	-* *-* *-	-* *-* *-

E5SEX3/EFV56656.1	diphthamide biosynthesis protein 1	1.77	-* *-* *-	2.10

E5S3A2/EFV60724.1	zinc finger protein	-* *-* *-	0.55	2.02

E5S8F0/EFV58915.1	putative actin-binding protein anillin	-* *-* *-	0.29	2.71

E5SFF4/EFV56450.1	GTP-binding ADP-ribosylation factor	-* *-* *-	2.44	-* *-* *-

E5SFH3/EFV56469	chromatin regulator subfamily B member 1	1.60	-* *-* *-	-* *-* *-

E5SB63/EFV58014.1	DNA topoisomerase 2-alpha	-* *-* *-	-* *-* *-	1.78

E5SGN0/EFV56116.1	histone acetyltransferase type B catalytic subunit	-* *-* *-	-* *-* *-	1.93

E5S4U4/EFV60187.1	putative thromboxane-A synthase	-* *-* *-	2.58	-* *-* *-

E5SJ08/EFV55221.1	eukaryotic translation initiation factor 3 subunit E, partial	-* *-* *-	3.39	-* *-* *-

E5RZQ0/EFV61978.1	hint module superfamily	-* *-* *-	6.66	-* *-* *-

E5SWW7/EFV50703.1	putative irregular chiasm C-roughest protein	-* *-* *-	2.01	-* *-* *-

E5S3J0/EFV60653.1	conserved hypothetical protein	1.96	0.40	-* *-* *-

E5S3I6/EFV60649.1	conserved hypothetical protein	-* *-* *-	2.54	-* *-* *-

E5SIR3/EFV55280.1	conserved hypothetical protein	-* *-* *-	2.02	-* *-* *-

E5S3U6/EFV60533.1	cuticle collagen 34 protein	0.28	0.45	0.59

E5SC11/EFV57673.1	Gut-specific cysteine proteinase	-* *-* *-	0.56	-* *-* *-

E5S2Q2/EFV60927.1	pyruvate dehydrogenase complex, E1 component,			
	pyruvate dehydrogenase, beta subunit	0.63	-* *-* *-	0.58

E5SET0/EFV56681.1	bestrophin-1	0.55	-* *-* *-	0.46

E5SC18/EFV57649.1	protein phosphatase PTC7-like protein	-* *-* *-	-* *-* *-	0.61

E5S7E3/EFV59293.1	phosphatidylinositol-3,4,5-trisphosphate 3-phosphatase PTEN	-* *-* *-	0.45	-* *-* *-

E5S8M1/EFV58830.1	Phosphoribosyl pyrophosphate synthetase-associated protein 2	0.60	-* *-* *-	-* *-* *-

E5SK74/EFV54817.1	putative BAR domain protein	0.65	-* *-* *-	0.60

E5RZN4/EFV61962.1	putative peptidase dimerization domain protein	-* *-* *-	0.55	-* *-* *-

E5SU43/EFV51698.1	conserved hypothetical protein	0.45	-* *-* *-	0.40

E5S2M0/EFV60948.1	conserved hypothetical protein, partial	0.57	-* *-* *-	0.53

E5RYF1/EFV62453.1	conserved domain protein	-* *-* *-	0.46	-* *-* *-

E5SDL6/EFV57109.1	conserved hypothetical protein	0.24	-* *-* *-	0.19

Differentially expressed proteins were defined using isobaric tags for relative and absolute quantitation (iTRAQ) as those with at least a 1.5 fold change relative to one another, with p < 0.05. Ratio data shows stage specific expression of proteins from Liu et al, 2016 [21]. ML, mature larvae, Ad3, adult L3 larvae, NBL, newborn larvae.

**Table 3:** *T. spiralis* PSGs correspond to *C. elegans* orthologs that confer severe RNAi phenotypes.

*T. spiralis* gene	*C. elegans* gene	Descriptor ^a^	*C. elegans* RNAi ^b^ phenotype
Tsp_09591/EFV53546.1	M05B5.2	hypothetical protein	Lon (long) Unc (uncoordinated) thin (decreased girth slim) Gro (slow growth)

Tsp_09505/EFV52757.1	W08F4.6	hypothetical protein	Prl (paralyzed) Unc (uncoordinated) Lva (larval arrest) Lvl (larval lethal) Bmd
			(body morphology defect) Ela (embryonic cell lineage abnormal)

EFV56123.1	F32H2.5	fatty acid synthase	Emb (embryonic lethal) Oth (other)

EFV54185.1	T26E3.3	PDZ domain	Emb (embryonic lethal) Spo (Abnormal embryonic spindle position and orientation)

EFV60927.1	C04C3.3	pyruvate dehydrogenase	Gro (slow growth) Emb (embryonic lethal)

EFV60724.1	Y40B1A.4	Zinc finger, C2H2 type	Unc (uncoordinated) Bmd (body morphology defect)

EFV56450.1	F54C9.10	GTP-binding	Emb (embryonic lethal)
		ADP-ribosylation factor	

EFV56859.1	T06E6.2a	cyb-3 cyclin B like (G2/mitotic-specific cyclin-B3)	Emb (embryonic lethal)

^a^Descriptor, annotation based on KEGG Orthology and Interpro.

^b^RNAi phenotype description (http://www.wormbase.org/).

## Data Availability

The data used to support the findings of this study are included within the article and the Supplementary Materials.

## Supplementary Materials

Supplementary Materials 2. Nucleotide sequences with more than 30% identity were selected and a total of 1997 orthologs were obtained (See Supplementary Dataset 1). Orthologs genes of five nematodes (A. ceylanicum, B. malayi, C. elegans, T. spiralis, and T. suis) for positive selection were identified. The data has been deposited in the orthologs genes of five nematodes (A. ceylanicum, B. malayi, C. elegans, T. spiralis, and T. suis) for positive selection repository. See Supplementary Dataset 1. 3. By using CODEML program to infer a dN/dS ratio for each pair of sequences, 986 genes were selected (p-value<0.01) (Supplementary Dataset 2). The data has been deposited in the positive selection genes of T. spiralis by comparing with four related nematodes. See Supplementary Dataset 2. 4. PSGs were grouped into the GO categories of biological processes, cellular components, and molecular functions (Supplementary Dataset 3). The data has been deposited in the GO analysis of PSGs in Trichinella spiralis by comparing with other four related nematodes. See Supplementary Dataset 3. 5. Gene Ontology (GO) term analysis for the positively selected genes of T. spiralis with four related nematodes (Figure 1). The figure has been deposited in the Gene Ontology (GO) term analysis for the positively selected genes of T. spiralis. See Figure 1 (included in the manuscript). 6. Molecular interaction and reaction networks of identified PSGs products were analyzed through KEGG pathway maps, which revealed that some PSGs could be ascribed to specific pathways, including metabolic pathways, the mRNA surveillance pathway, pentose phosphate pathway, amino sugar and nucleotide sugar, synthesis pathways, endocytosis, nucleotide excision repair, calcium signaling pathway, purine metabolism, inositol phosphate metabolism, and the phosphatidylinositol signaling system (Figure 2; Supplementary Dataset 4). The data has been deposited in the Pathway Information of Kyoto Encyclopedia of Genes and Genomes (KEGG) of positively selected genes of Trichinella spiralis. See Supplementary Dataset 4. The figure has been deposited in the pathway enrichment of positively selected genes in T. spiralis as defined by KEGG Pathway maps. See Figure 2 (included in the manuscript). 7. The pathways delineated proteins that may participate in nurse cell formation: modification of metabolic pathways in the host cells, creation of new parasite-specific morphological structures between T. spiralis and the host, control of xenobiotic metabolism, when contending with low oxygen concentrations and host toxicity, transformation of muscle cells, regulation of the cell cycle and DNA repair processes and antiapoptotic events during nurse cell formation, immunomodulation, and regulation of epigenetic processes (Table 1). The table has been deposited in the gene functions for some of the positively selected genes identified in T. spiralis. See Table 1 (included in the manuscript). 8. Differentially expressed proteins, identified by Liu et al. [21], were defined using isobaric tags for relative and absolute quantitation (iTRAQ) as those with at least a 1.5-fold change relative to one another, with p < 0.05. The related link was https://www.sciencedirect.com/science/article/pii/S0304401716302291?via%3Dihub. The data source was from the author. In the current study, we examined the corresponding genes for the presence of PSGs and identified 57 T. spiralis PSGs that are differentially expressed in different life-cycle stages (Table 2). The table has been deposited in the T. spiralis PSGs encoding differentially expressed proteins in adults, muscle larvae and newborn larvae stages. See Table 2 (included in the manuscript). 9. In a previous report, 463 T. spiralis genes were identified that have C. elegans orthologs that confer RNAi phenotypes (https://www.wormbase.org/) [22]. The link was https://ac.els-cdn.com/S0166685104001793/1-s2.0-S0166685104001793-main.pdf?_tid=7e4f7930-38ba-480e-8dfa-93ebe0bcc94c&acdnat=1538385967_01d2d0b31d5f0fb38e90bad5ded7241c. See Table S5: complete list of C. elegans RNAi phenotypes for genes with T. spiralis homologs. PSGs that overlapped with these 463 T. spiralis genes with C. elegans RNAi orthologs were identified. These T. spiralis genes were compared to the PSG list to determine if any of these orthologs have adaptive potential.  Table 3 shows a partial list of the identified PSGs in T. spiralis, focusing on those that conferred lethal or severe phenotypes in C. elegans. The table has been deposited in the T. spiralis PSGs correspond to C. elegans orthologs that confer severe RNAi phenotypes. See Table 3 (included in the manuscript).
