# Supplementary material for: Comparative Genomic Analysis of Trichinella spiralis Reveals Potential Mechanisms of Adaptive Evolution
Source: Biomed Res Int. 2019 May 21;2019:2948973. doi: 10.1155/2019/2948973 (PMC6556364; doi:10.1155/2019/2948973)
Supplement: Supplementary Materials — 2. Nucleotide sequences with more than 30% identity were selected and a total of 1997 orthologs were obtained (See Supplementary Dataset 1). Orthologs genes of five nematodes (A. ceylanicum, B. malayi, C. elegans, T. spiralis, and T. suis) for positive selection were identified. The data has been deposited in the orthologs genes of five nematodes (A. ceylanicum, B. malayi, C. elegans, T. spiralis, and T. suis) for positive selection repository. See Supplementary Dataset 1. 3. By using CODEML program to infer a dN/dS ratio for each pair of sequences, 986 genes were selected (p-value<0.01) (Supplementary Dataset 2). The data has been deposited in the positive selection genes of T. spiralis by comparing with four related nematodes. See Supplementary Dataset 2. 4. PSGs were grouped into the GO categories of biological processes, cellular components, and molecular functions (Supplementary Dataset 3). The data has been deposited in the GO analysis of PSGs in Trichinella spiralis by comparing with other four related nematodes. See Supplementary Dataset 3. 5. Gene Ontology (GO) term analysis for the positively selected genes of T. spiralis with four related nematodes (Figure 1). The figure has been deposited in the Gene Ontology (GO) term analysis for the positively selected genes of T. spiralis. See Figure 1 (included in the manuscript). 6. Molecular interaction and reaction networks of identified PSGs products were analyzed through KEGG pathway maps, which revealed that some PSGs could be ascribed to specific pathways, including metabolic pathways, the mRNA surveillance pathway, pentose phosphate pathway, amino sugar and nucleotide sugar, synthesis pathways, endocytosis, nucleotide excision repair, calcium signaling pathway, purine metabolism, inositol phosphate metabolism, and the phosphatidylinositol signaling system (Figure 2; Supplementary Dataset 4). The data has been deposited in the Pathway Information of Kyoto Encyclopedia of Genes and Genomes (KEGG) of positively [file 2948973.f1.zip › supplementary files/Supplementary dataset 2 Positive selection genes of T. spiralis by comparing with four related nematodes.docx]

**Positively selected genes of *T. spiralis* identified by comparing four related nematodes**

| **GenBank No.** | ***p*-value** | **gene description** |
| --- | --- | --- |
| EFV46349.1 | 0 | innexin-11 |
| EFV46647.1 | 0 | AMP deaminase (Myoadenylate deaminase) |
| EFV46914.1 | 0 | conserved hypothetical protein, partial |
| EFV46951.1 | 0 | putative fibronectin type III domain protein, partial |
| EFV47048.1 | 0 | histone H2A type 1, partial |
| EFV47329.1 | 0 | conserved hypothetical protein |
| EFV47343.1 | 0 | phosphatidylcholine:ceramide cholinephosphotransferase 2, partial |
| EFV47512.1 | 0 | signal recognition particle protein, partial |
| EFV47608.1 | 0 | WD domain, G-beta repeat-containing domain protein |
| EFV47803.1 | 0 | phosphatidylinositol-4,5-bisphosphate phosphodiesterase beta-4 |
| EFV47834.1 | 0 | sodium/calcium exchanger 1 |
| EFV47885.1 | 0 | putative fibronectin type III domain protein, partial |
| EFV48146.1 | 0 | angiogenic factor with G patch and FHA domain 1 |
| EFV48151.1 | 0 | DNA ligase 1 |
| EFV48228.1 | 0 | protein NDRG3 |
| EFV48290.1 | 0 | conserved hypothetical protein, partial |
| EFV48442.1 | 0 | vacuolar ATP synthase catalytic subunit A, partial |
| EFV48461.1 | 0 | ribosomal protein L22 |
| EFV48512.1 | 0 | putative SH3 domain protein |
| EFV48526.1 | 0 | long-chain fatty acid transport protein 1, partial |
| EFV48558.1 | 0 | eukaryotic translation initiation factor 2C 2, partial |
| EFV48808.1 | 0 | SPARC protein |
| EFV48926.1 | 0 | putative ABC transporter, ATP-binding protein, partial |
| EFV48950.1 | 0 | conserved hypothetical protein |
| EFV48975.1 | 0 | Ion channel superfamily, partial |
| EFV49205.1 | 0 | putative ribonuclease H1, partial |
| EFV49235.1 | 0 | tuftelin-interacting protein 11, partial |
| EFV49296.1 | 0 | COP9 signalosome complex subunit 2 |
| EFV49322.1 | 0 | conserved hypothetical protein, partial |
| EFV49327.1 | 0 | tRNA guanosine-2'-O-methyltransferase TRM11-like protein, partial |
| EFV49402.1 | 0 | DNA mismatch repair protein Msh6 |
| EFV49415.1 | 0 | putative beige/BEACH domain protein |
| EFV49435.1 | 0 | eukaryotic translation initiation factor 2 subunit 3, partial |
| EFV49477.1 | 0 | putative nucleolar complex protein 4, partial |
| EFV49567.1 | 0 | putative immunoglobulin domain protein |
| EFV49612.1 | 0 | gremlin-1 |
| EFV49637.1 | 0 | splicing factor 3A subunit 2, partial |
| EFV49642.1 | 0 | phosphotyrosine interaction domain protein, partial |
| EFV49650.1 | 0 | CDP-diacylglycerol--glycerol-3-phosphate3-phosphatidyltransferase partial |
| EFV49666.1 | 0 | putative calponin |
| EFV49690.1 | 0 | kynurenine--oxoglutarate transaminase, partial |
| EFV49715.1 | 0 | putative ATP synthase F1, gamma subunit, partial |
| EFV49737.1 | 0 | putative amphiphysin |
| EFV49768.1 | 0 | WD repeat domain phosphoinositide-interacting protein 4 |
| EFV49855.1 | 0 | casein kinase I isoform alpha, partial |
| EFV49875.1 | 0 | putative calcium binding EGF domain protein, partial |
| EFV49905.1 | 0 | putative translocon-associated protein subunit alpha |
| EFV49924.1 | 0 | tyrosine-protein phosphatase non-receptor type 4 |
| EFV49955.1 | 0 | RtcB protein, partial |
| EFV50049.1 | 0 | Ham1 family protein |
| EFV50052.1 | 0 | putative TRAF and TNF receptor-associated protein |
| EFV50053.1 | 0 | putative NADH dehydrogenase [ubiquinone] 1 beta subcomplex subunit3 |
| EFV50066.1 | 0 | copine subfamily |
| EFV50166.1 | 0 | RNA-binding protein 42 |
| EFV50200.1 | 0 | heparan sulfate glucosamine 3-O-sulfotransferase 3A1 |
| EFV50242.1 | 0 | putative translocon-associated protein subunit delta |
| EFV50268.1 | 0 | conserved hypothetical protein |
| EFV50278.1 | 0 | 7 transmembrane receptor |
| EFV50305.1 | 0 | SNW domain-containing protein 1 |
| EFV50309.1 | 0 | NADH-ubiquinone oxidoreductase ASHI subunit |
| EFV50315.1 | 0 | replication factor C subunit 3 |
| EFV50316.1 | 0 | cytochrome P450 4V2 |
| EFV50329.1 | 0 | peptidase, M16 family |
| EFV50332.1 | 0 | 6-pyruvoyl tetrahydrobiopterin synthase |
| EFV50357.1 | 0 | putative 3-hydroxyacyl-CoA dehydrogenase, NAD binding domain protein |
| EFV50370.1 | 0 | eukaryotic translation initiation factor 2C 2 |
| EFV50374.1 | 0 | eukaryotic translation initiation factor 2C 2 |
| EFV50386.1 | 0 | putative transcription factor COE1 |
| EFV50422.1 | 0 | conserved hypothetical protein |
| EFV50459.1 | 0 | putative GYF domain protein |
| EFV50471.1 | 0 | cell division protein |
| EFV50496.1 | 0 | protein strawberry notch |
| EFV50515.1 | 0 | vacuolar protein sorting-associated protein 35 |
| EFV50579.1 | 0 | serine/threonine-protein phosphatase 2A regulatory subunit B |
| EFV50595.1 | 0 | splicing factor 3B subunit 3 |
| EFV50617.1 | 0 | tRNA (adenine-N(1)-)-methyltransferase catalytic subunit TRM61 |
| EFV50642.1 | 0 | putative ABC transporter, ATP-binding protein |
| EFV50644.1 | 0 | putative CD82 antigen |
| EFV50651.1 | 0 | putative PAN domain protein |
| EFV50660.1 | 0 | cytochrome b5 |
| EFV50700.1 | 0 | transmembrane protein 41B |
| EFV50701.1 | 0 | zinc finger CCCH domain-containing protein 15 |
| EFV50746.1 | 0 | putative FERM central domain protein |
| EFV50852.1 | 0 | elongation factor 1-beta' |
| EFV50886.1 | 0 | beta-galactosidase |
| EFV50920.1 | 0 | protein unc-50 |
| EFV50975.1 | 0 | protein arginine N-methyltransferase 1 |
| EFV50984.1 | 0 | phosphopantothenoylcysteine decarboxylase |
| EFV51123.1 | 0 | 60S ribosomal protein L36 |
| EFV51148.1 | 0 | transmembrane protein 49 |
| EFV51149.1 | 0 | phosphoglycerate kinase |
| EFV51151.1 | 0 | putative PAN domain protein |
| EFV51193.1 | 0 | vesicular GABA transporter |
| EFV51202.1 | 0 | putative methyltransferase BUD23 |
| EFV51271.1 | 0 | poly(A) polymerase |
| EFV51308.1 | 0 | cytochrome c oxidase subunit IV |
| EFV51309.1 | 0 | N-acetyltransferase 11 |
| EFV51368.1 | 0 | conserved hypothetical protein |
| EFV51374.1 | 0 | putative I/LWEQ domain protein, partial |
| EFV51389.1 | 0 | putative iroquois-class homeodomain protein IRX-6 |
| EFV51418.1 | 0 | tropomyosin |
| EFV51437.1 | 0 | diphosphomevalonate decarboxylase |
| EFV51439.1 | 0 | acetyl-coenzyme A transporter 1 |
| EFV51487.1 | 0 | conserved hypothetical protein |
| EFV51492.1 | 0 | ceramide glucosyltransferase |
| EFV51535.1 | 0 | putative ATP-dependent Clp protease, ATP-binding subunit ClpX |
| EFV51541.1 | 0 | zinc finger protein |
| EFV51542.1 | 0 | putative N(6)-adenine-specific DNA methyltransferase 1 |
| EFV51544.1 | 0 | putative HEAT protein |
| EFV51562.1 | 0 | putative PX domain protein |
| EFV51563.1 | 0 | putative serine/arginine repetitive matrix protein 1 |
| EFV51565.1 | 0 | putative serine/threonine-protein phosphatase 4 regulatory subunit1 |
| EFV51566.1 | 0 | putative tetratricopeptide protein |
| EFV51623.1 | 0 | putative type I inositol-1,4,5-trisphosphate 5-phosphatase |
| EFV51636.1 | 0 | putative CUB domain protein |
| EFV51659.1 | 0 | la-related protein 4 |
| EFV51677.1 | 0 | 1,4-alpha-glucan-branching enzyme |
| EFV51739.1 | 0 | hypothetical protein Tsp_10574 |
| EFV51745.1 | 0 | oxidoreductase, short chain dehydrogenase/reductase family |
| EFV51763.1 | 0 | 7 transmembrane receptor |
| EFV51773.1 | 0 | josephin-2 |
| EFV51796.1 | 0 | mucolipin-1 |
| EFV51842.1 | 0 | sodium-dependent phosphate transporter 1-A |
| EFV51845.1 | 0 | conserved hypothetical protein |
| EFV51851.1 | 0 | hypothetical protein Tsp_11967 |
| EFV51898.1 | 0 | charged multivesicular body protein 1b |

| EFV51914.1 | 0 | | | | | | | | | | | | | | | | histidine triad nucleotide-binding protein 1 | | | | | | | | | |  |  |  |  |  |  |  |  |  |  |  |  |  |  |
| --- | --- | --- | --- | --- | --- | --- | --- | --- | --- | --- | --- | --- | --- | --- | --- | --- | --- | --- | --- | --- | --- | --- | --- | --- | --- | --- | --- | --- | --- | --- | --- | --- | --- | --- | --- | --- | --- | --- | --- | --- |
| EFV51950.1 | 0 | | | | | | | | | | | | | | | | conserved hypothetical protein | | | | | | | | | |  |  |  |  |  |  |  |  |  |  |  |  |  |  |
| EFV51952.1 | 0 | | | | | | | | | | | | | | | | NADH dehydrogenase | | | | | | | | | |  |  |  |  |  |  |  |  |  |  |  |  |  |  |
| EFV51955.1 | 0 | | | | | | | | | | | | | | | | TPPP family protein C32E8.3 | | | | | | | | | |  |  |  |  |  |  |  |  |  |  |  |  |  |  |
| EFV51962.1 | 0 | | | | | | | | | | | | | | | | hydrolase, HAD superfamily | | | | | | | | | |  |  |  |  |  |  |  |  |  |  |  |  |  |  |
| EFV51964.1 | 0 | | | | | | | | | | | | | | | | conserved hypothetical protein | | | | | | | | | |  |  |  |  |  |  |  |  |  |  |  |  |  |  |
| EFV51984.1 | 0 | | | | | | | | | | | | | | | | sorcin | | | | | | | | | |  |  |  |  |  |  |  |  |  |  |  |  |  |  |
| EFV51987.1 | 0 | | | | | | | | | | | | | | | | nucampholin-like protein | | | | | | | | | |  |  |  |  |  |  |  |  |  |  |  |  |  |  |
| EFV52019.1 | 0 | | | | | | | | | | | | | | | | putative RhoGEF domain protein | | | | | | | | | |  |  |  |  |  |  |  |  |  |  |  |  |  |  |
| EFV52023.1 | 0 | | | | | | | | | | | | | | | | single-stranded DNA-binding protein 4 | | | | | | | | | |  |  |  |  |  |  |  |  |  |  |  |  |  |  |
| EFV52027.1 | 0 | | | | | | | | | | | | | | | | putative fasciclin domain protein | | | | | | | | | |  |  |  |  |  |  |  |  |  |  |  |  |  |  |
| EFV52032.1 | 0 | | | | | | | | | | | | | | | | 26S proteasome non-ATPase regulatory subunit 7 | | | | | | | | | |  |  |  |  |  |  |  |  |  |  |  |  |  |  |
| EFV52052.1 | 0 | | | | | | | | | | | | | | | | zinc finger protein | | | | | | | | | |  |  |  |  |  |  |  |  |  |  |  |  |  |  |
| EFV52061.1 | 0 | | | | | | | | | | | | | | | | cytochrome c oxidase assembly protein CtaG | | | | | | | | | |  |  |  |  |  |  |  |  |  |  |  |  |  |  |
| EFV52066.1 | 0 | | | | | | | | | | | | | | | | sorting nexin-2 | | | | | | | | | |  |  |  |  |  |  |  |  |  |  |  |  |  |  |
| EFV52074.1 | 0 | | | | | | | | | | | | | | | | dynactin subunit 5 | | | | | | | | | |  |  |  |  |  |  |  |  |  |  |  |  |  |  |
| EFV52079.1 | 0 | | | | | | | | | | | | | | | | charged multivesicular body protein 4c | | | | | | | | | |  |  |  |  |  |  |  |  |  |  |  |  |  |  |
| EFV52084.1 | 0 | | | | | | | | | | | | | | | | cyclic AMP-responsive element-binding protein 3 | | | | | | | | | |  |  |  |  |  |  |  |  |  |  |  |  |  |  |
| EFV52088.1 | 0 | | | | | | | | | | | | | | | | [LSM14 protein](https://www.ncbi.nlm.nih.gov/protein/EFV52088.1/) | | | | | | | | | |  |  |  |  |  |  |  |  |  |  |  |  |  |  |
| EFV52092.1 | 0 | | | | | | | | | | | | | | | | dynactin subunit 5 | | | | | | | | | |  |  |  |  |  |  |  |  |  |  |  |  |  |  |
| EFV52135.1 | 0 | | | | | | | | | | | | | | | | guanylate cyclase 32E | | | | | | | | | |  |  |  |  |  |  |  |  |  |  |  |  |  |  |
| EFV52161.1 | 0 | | | | | | | | | | | | | | | | hypothetical protein Tsp_05540 | | | | | | | | | |  |  |  |  |  |  |  |  |  |  |  |  |  |  |
| EFV52168.1 | 0 | | | | | | | | | | | | | | | | putative thrombospondin type 1 domain protein | | | | | | | | | |  |  |  |  |  |  |  |  |  |  |  |  |  |  |
| EFV52174.1 | 0 | | | | | | | | | | | | | | | | DNA-binding protein A | | | | | | | | | |  |  |  |  |  |  |  |  |  |  |  |  |  |  |
| EFV52187.1 | 0 | | | | | | | | | | | | | | | | 4-hydroxybutyrate CoA-transferase | | | | | | | | | |  |  |  |  |  |  |  |  |  |  |  |  |  |  |
| EFV52191.1 | 0 | | | | | | | | | | | | | | | | ubiquitin carboxyl- hydrolase 6 | | | | | | | | | |  |  |  |  |  |  |  |  |  |  |  |  |  |  |
| EFV52224.1 | 0 | | | | | | | | | | | | | | | | putative trimeric intracellular cation channel type B | | | | | | | | | |  |  |  |  |  |  |  |  |  |  |  |  |  |  |
| EFV52245.1 | 0 | | | | | | | | | | | | | | | | conserved hypothetical protein, partial | | | | | | | | | |  |  |  |  |  |  |  |  |  |  |  |  |  |  |
| EFV52253.1 | 0 | | | | | | | | | | | | | | | | coiled-coil domain-containing protein 94 | | | | | | | | | |  |  |  |  |  |  |  |  |  |  |  |  |  |  |
| EFV52292.1 | 0 | | | | | | | | | | | | | | | | putative ADP-dependent glucokinase | | | | | | | | | |  |  |  |  |  |  |  |  |  |  |  |  |  |  |
| EFV52329.1 | 0 | | | | | | | | | | | | | | | | SAC3/GANP family protein | | | | | | | | | |  |  |  |  |  |  |  |  |  |  |  |  |  |  |
| EFV52332.1 | 0 | | | | | | | | | | | | | | | | cleavage and polyadenylation specificity factor subunit 1 | | | | | | | | | |  |  |  |  |  |  |  |  |  |  |  |  |  |  |
| EFV52363.1 | 0 | | | | | | | | | | | | | | | | putative TPP requiring enzyme, partial | | | | | | | | | |  |  |  |  |  |  |  |  |  |  |  |  |  |  |
| EFV52385.1 | 0 | | | | | | | | | | | | | | | | triosephosphate isomerase | | | | | | | | | |  |  |  |  |  |  |  |  |  |  |  |  |  |  |
| EFV52403.1 | 0 | | | | | | | | | | | | | | | | paramyosin | | | | | | | | | |  |  |  |  |  |  |  |  |  |  |  |  |  |  |
| EFV52405.1 | 0 | | | | | | | | | | | | | | | | conserved hypothetical protein | | | | | | | | | |  |  |  |  |  |  |  |  |  |  |  |  |  |  |
| EFV52412.1 | 0 | | | | | | | | | | | | | | | | conserved hypothetical protein | | | | | | | | | |  |  |  |  |  |  |  |  |  |  |  |  |  |  |
| EFV52426.1 | 0 | | | | | | | | | | | | | | | | conserved domain protein | | | | | | | | | |  |  |  |  |  |  |  |  |  |  |  |  |  |  |
| EFV52428.1 | | 0 | | | | | | | | | | | | | | | multicatalytic endopeptidase | | | | | | | | |  |  |  |  |  |  |  |  |  |  |  |  |  |  |  |
| EFV52429.1 | | 0 | | | | | | | | | | | | | | | vacuolar proton pump subunit D 1 | | | | | | | | |  |  |  |  |  |  |  |  |  |  |  |  |  |  |  |
| EFV52537.1 | | 0 | | | | | | | | | | | | | | | chord superfamily | | | | | | | | |  |  |  |  |  |  |  |  |  |  |  |  |  |  |  |
| EFV52578.1 | | 0 | | | | | | | | | | | | | | | myophilin | | | | | | | | |  |  |  |  |  |  |  |  |  |  |  |  |  |  |  |
| EFV52580.1 | | 0 | | | | | | | | | | | | | | | putative ANTH domain protein | | | | | | | | |  |  |  |  |  |  |  |  |  |  |  |  |  |  |  |
| EFV52591.1 | | 0 | | | | | | | | | | | | | | | putative radical SAM domain protein | | | | | | | | |  |  |  |  |  |  |  |  |  |  |  |  |  |  |  |
| EFV52599.1 | | 0 | | | | | | | | | | | | | | | conserved hypothetical protein | | | | | | | | |  |  |  |  |  |  |  |  |  |  |  |  |  |  |  |
| EFV52604.1 | | 0 | | | | | | | | | | | | | | | eyes absent protein | | | | | | | | |  |  |  |  |  |  |  |  |  |  |  |  |  |  |  |
| EFV52612.1 | | 0 | | | | | | | | | | | | | | | putative transcription factor Sp8 | | | | | | | | |  |  |  |  |  |  |  |  |  |  |  |  |  |  |  |
| EFV52618.1 | | 0 | | | | | | | | | | | | | | | chaperonin, 10 kDa | | | | | | | | |  |  |  |  |  |  |  |  |  |  |  |  |  |  |  |
| EFV52620.1 | | 0 | | | | | | | | | | | | | | | peptidyl-prolyl cis-trans isomerase SDCCAG10 | | | | | | | | |  |  |  |  |  |  |  |  |  |  |  |  |  |  |  |
| EFV52650.1 | | 0 | | | | | | | | | | | | | | | putative RNA recognition motif protein | | | | | | | | |  |  |  |  |  |  |  |  |  |  |  |  |  |  |  |
| EFV52660.1 | | 0 | | | | | | | | | | | | | | | insulin protein enhancer protein ISL-1 | | | | | | | | |  |  |  |  |  |  |  |  |  |  |  |  |  |  |  |
| EFV52663.1 | | 0 | | | | | | | | | | | | | | | putative LIM domain protein | | | | | | | | |  |  |  |  |  |  |  |  |  |  |  |  |  |  |  |
| EFV52676.1 | | 0 | | | | | | | | | | | | | | | calreticulin | | | | | | | | |  |  |  |  |  |  |  |  |  |  |  |  |  |  |  |
| EFV52681.1 | | 0 | | | | | | | | | | | | | | | OTU domain-containing protein 7B | | | | | | | | |  |  |  |  |  |  |  |  |  |  |  |  |  |  |  |
| EFV52683.1 | | 0 | | | | | | | | | | | | | | | splicing factor 45 | | | | | | | | |  |  |  |  |  |  |  |  |  |  |  |  |  |  |  |
| EFV52694.1 | | 0 | | | | | | | | | | | | | | | cuticle collagen rol-6 | | | | | | | | |  |  |  |  |  |  |  |  |  |  |  |  |  |  |  |
| EFV52702.1 | | 0 | | | | | | | | | | | | | | | putative ribosomal protein L16 | | | | | | | | |  |  |  |  |  |  |  |  |  |  |  |  |  |  |  |
| EFV52706.1 | | 0 | | | | | | | | | | | | | | | cuticle collagen dpy-7 | | | | | | | | |  |  |  |  |  |  |  |  |  |  |  |  |  |  |  |
| EFV52708.1 | | 0 | | | | | | | | | | | | | | | MYND finger protein | | | | | | | | |  |  |  |  |  |  |  |  |  |  |  |  |  |  |  |
| EFV52719.1 | | 0 | | | | | | | | | | | | | | | putative protein lin-10 | | | | | | | | |  |  |  |  |  |  |  |  |  |  |  |  |  |  |  |
| EFV52749.1 | | 0 | | | | | | | | | | | | | | | potassium voltage-gated channel protein Shaw | | | | | | | | |  |  |  |  |  |  |  |  |  |  |  |  |  |  |  |
| EFV52750.1 | | 0 | | | | | | | | | | | | | | | adenylate kinase isoenzyme 6 | | | | | | | | |  |  |  |  |  |  |  |  |  |  |  |  |  |  |  |
| EFV52755.1 | | 0 | | | | | | | | | | | | | | | putative ATPase, AAA family | | | | | | | | |  |  |  |  |  |  |  |  |  |  |  |  |  |  |  |
| EFV52757.1 | | 0 | | | | | | | | | | | | | | | conserved hypothetical protein | | | | | | | | |  |  |  |  |  |  |  |  |  |  |  |  |  |  |  |
| EFV52770.1 | | 0 | | | | | | | | | | | | | | | retinal rod rhodopsin-sensitive cGMP 3',5'-cyclic phosphodiesterase subunit delta | | | | | | | | |  |  |  |  |  |  |  |  |  |  |  |  |  |  |  |
| EFV52783.1 | | 0 | | | | | | | | | | | | | | | conserved hypothetical protein | | | | | | | | |  |  |  |  |  |  |  |  |  |  |  |  |  |  |  |
| EFV52792.1 | | 0 | | | | | | | | | | | | | | | fatty-acid amide hydrolase 2-A | | | | | | | | |  |  |  |  |  |  |  |  |  |  |  |  |  |  |  |
| EFV52803.1 | | 0 | | | | | | | | | | | | | | | conserved hypothetical protein | | | | | | | | |  |  |  |  |  |  |  |  |  |  |  |  |  |  |  |
| EFV52807.1 | | 0 | | | | | | | | | | | | | | | N-acetylgalactosamine kinase | | | | | | | | |  |  |  |  |  |  |  |  |  |  |  |  |  |  |  |
| EFV52813.1 | | 0 | | | | | | | | | | | | | | | conserved hypothetical protein | | | | | | | | |  |  |  |  |  |  |  |  |  |  |  |  |  |  |  |
| EFV52814.1 | | 0 | | | | | | | | | | | | | | | alpha-1,2-mannosyltransferase ALG9 | | | | | | | | |  |  |  |  |  |  |  |  |  |  |  |  |  |  |  |
| EFV52822.1 | | 0 | | | | | | | | | | | | | | | enolase | | | | | | | | |  |  |  |  |  |  |  |  |  |  |  |  |  |  |  |
| EFV52831.1 | | 0 | | | | | | | | | | | | | | | putativeUDP-glucose/GDP-mannose dehydrogenase family,  central domain protein | | | | | | | | |  |  |  |  |  |  |  |  |  |  |  |  |  |  |  |
| EFV52845.1 | | 0 | | | | | | | | | | | | | | | cuticle collagen dpy-13 | | | | | | | | |  |  |  |  |  |  |  |  |  |  |  |  |  |  |  |
| EFV52847.1 | | 0 | | | | | | | | | | | | | | | putative DEAD/DEAH box helicase | | | | | | | | |  |  |  |  |  |  |  |  |  |  |  |  |  |  |  |
| EFV52856.1 | | 0 | | | | | | | | | | | | | | | hypothetical protein Tsp_09426 | | | | | | | | |  |  |  |  |  |  |  |  |  |  |  |  |  |  |  |
| EFV52864.1 | | | 0 | | | | | | | | | | | | | | | sodium Bile acid symporter family protein | | | | | | | |  |  |  |  |  |  |  |  |  |  |  |  |  |  |  |
| EFV52868.1 | | | 0 | | | | | | | | | | | | | | | N-acetylgalactosamine kinase | | | | | | | |  |  |  |  |  |  |  |  |  |  |  |  |  |  |  |
| EFV52876.1 | | | 0 | | | | | | | | | | | | | | | dihydropyridine sensitive L-type calcium channel family protein | | | | | | | |  |  |  |  |  |  |  |  |  |  |  |  |  |  |  |
| EFV52879.1 | | | 0 | | | | | | | | | | | | | | | Rho GTPase-activating protein 8 | | | | | | | |  |  |  |  |  |  |  |  |  |  |  |  |  |  |  |
| EFV52894.1 | | | 0 | | | | | | | | | | | | | | | putative PAN domain protein | | | | | | | |  |  |  |  |  |  |  |  |  |  |  |  |  |  |  |
| EFV52896.1 | | | 0 | | | | | | | | | | | | | | | endonuclease/exonuclease/phosphatase family protein | | | | | | | |  |  |  |  |  |  |  |  |  |  |  |  |  |  |  |
| EFV52898.1 | | | 0 | | | | | | | | | | | | | | | hypothetical protein Tsp_09420 | | | | | | | |  |  |  |  |  |  |  |  |  |  |  |  |  |  |  |
| EFV52901.1 | | | 0 | | | | | | | | | | | | | | | putative biotin--protein ligase | | | | | | | |  |  |  |  |  |  |  |  |  |  |  |  |  |  |  |
| EFV52919.1 | | | 0 | | | | | | | | | | | | | | | hypothetical protein Tsp_10478 | | | | | | | |  |  |  |  |  |  |  |  |  |  |  |  |  |  |  |
| EFV52920.1 | | | 0 | | | | | | | | | | | | | | | conserved hypothetical protein | | | | | | | |  |  |  |  |  |  |  |  |  |  |  |  |  |  |  |
| EFV52930.1 | | | 0 | | | | | | | | | | | | | | | putative chondroitin sulfate synthase 3 | | | | | | | |  |  |  |  |  |  |  |  |  |  |  |  |  |  |  |
| EFV52936.1 | | | 0 | | | | | | | | | | | | | | | putative eukaryotic-type carbonic anhydrase | | | | | | | |  |  |  |  |  |  |  |  |  |  |  |  |  |  |  |
| EFV52939.1 | | | 0 | | | | | | | | | | | | | | | arrestin protein | | | | | | | |  |  |  |  |  |  |  |  |  |  |  |  |  |  |  |
| EFV52942.1 | | | 0 | | | | | | | | | | | | | | | putative ATP synthase F1, epsilon subunit | | | | | | | |  |  |  |  |  |  |  |  |  |  |  |  |  |  |  |
| EFV52984.1 | | | 0 | | | | | | | | | | | | | | | protein maternal effect lethal 26 | | | | | | | |  |  |  |  |  |  |  |  |  |  |  |  |  |  |  |
| EFV52985.1 | | | 0 | | | | | | | | | | | | | | | putative PLAT/LH2 domain protein | | | | | | | |  |  |  |  |  |  |  |  |  |  |  |  |  |  |  |
| EFV53000.1 | | | 0 | | | | | | | | | | | | | | | M-phase inducer phosphatase 1-B | | | | | | | |  |  |  |  |  |  |  |  |  |  |  |  |  |  |  |
| EFV53011.1 | | | 0 | | | | | | | | | | | | | | | hypothetical protein Tsp_10404 | | | | | | | |  |  |  |  |  |  |  |  |  |  |  |  |  |  |  |
| EFV53012.1 | | | 0 | | | | | | | | | | | | | | | putative snoRNA binding domain protein | | | | | | | |  |  |  |  |  |  |  |  |  |  |  |  |  |  |  |
| EFV53025.1 | | | 0 | | | | | | | | | | | | | | | putative myosin head | | | | | | | |  |  |  |  |  |  |  |  |  |  |  |  |  |  |  |
| EFV53035.1 | | | 0 | | | | | | | | | | | | | | | GTP-dependent nucleic acid-binding protein EngD | | | | | | | |  |  |  |  |  |  |  |  |  |  |  |  |  |  |  |
| EFV53038.1 | | | 0 | | | | | | | | | | | | | | | complex I intermediate-associated protein 30 (CIA30) | | | | | | | |  |  |  |  |  |  |  |  |  |  |  |  |  |  |  |
| EFV53039.1 | | | 0 | | | | | | | | | | | | | | | conserved hypothetical protein | | | | | | | |  |  |  |  |  |  |  |  |  |  |  |  |  |  |  |
| EFV53075.1 | | | 0 | | | | | | | | | | | | | | | LMBR1 domain-containing protein 2 | | | | | | | |  |  |  |  |  |  |  |  |  |  |  |  |  |  |  |
| EFV53090.1 | | | 0 | | | | | | | | | | | | | | | inositol-trisphosphate 3-kinase B | | | | | | | |  |  |  |  |  |  |  |  |  |  |  |  |  |  |  |
| EFV53092.1 | | | 0 | | | | | | | | | | | | | | | putative activin types I and II receptor domain protein | | | | | | | |  |  |  |  |  |  |  |  |  |  |  |  |  |  |  |
| EFV53122.1 | | | 0 | | | | | | | | | | | | | | | putative helix-loop-helix DNA-binding domain protein | | | | | | | |  |  |  |  |  |  |  |  |  |  |  |  |  |  |  |
| EFV53154.1 | | | 0 | | | | | | | | | | | | | | | cyclin-L2 | | | | | | | |  |  |  |  |  |  |  |  |  |  |  |  |  |  |  |
| EFV53158.1 | | | 0 | | | | | | | | | | | | | | | putative caspase recruitment domain protein | | | | | | | |  |  |  |  |  |  |  |  |  |  |  |  |  |  |  |
| EFV53177.1 | | | 0 | | | | | | | | | | | | | | | putative nuclear transcription factor Y subunit alpha | | | | | | | |  |  |  |  |  |  |  |  |  |  |  |  |  |  |  |
| EFV53182.1 | | | 0 | | | | | | | | | | | | | | | leukocyte receptor cluster member 8-like protein | | | | | | | |  |  |  |  |  |  |  |  |  |  |  |  |  |  |  |
| EFV53312.1 | | | 0 | | | | | | | | | | | | | | | phosphatidylinositol transfer protein alpha isoform | | | | | | | |  |  |  |  |  |  |  |  |  |  |  |  |  |  |  |
| EFV53314.1 | | | 0 | | | | | | | | | | | | | | | sodium-dependent multivitamin transporter | | | | | | | |  |  |  |  |  |  |  |  |  |  |  |  |  |  |  |
| EFV53374.1 | | | 0 | | | | | | | | | | | | | | | CDK5 and ABL1 enzyme substrate 1 | | | | | | | |  |  |  |  |  |  |  |  |  |  |  |  |  |  |  |
| EFV53375.1 | | | 0 | | | | | | | | | | | | | | | zinc finger protein | | | | | | | |  |  |  |  |  |  |  |  |  |  |  |  |  |  |  |
| EFV53377.1 | | | 0 | | | | | | | | | | | | | | | WD domain, G-beta repeat-containing domain protein | | | | | | | |  |  |  |  |  |  |  |  |  |  |  |  |  |  |  |
| EFV53400.1 | | | 0 | | | | | | | | | | | | | | | GNT-I family protein | | | | | | | |  |  |  |  |  |  |  |  |  |  |  |  |  |  |  |
| EFV53432.1 | | | 0 | | | | | | | | | | | | | | | putative SH2 domain protein | | | | | | | |  |  |  |  |  |  |  |  |  |  |  |  |  |  |  |
| EFV53488.1 | | | 0 | | | | | | | | | | | | | | | conserved hypothetical protein | | | | | | | |  |  |  |  |  |  |  |  |  |  |  |  |  |  |  |
| EFV53497.1 | | | 0 | | | | | | | | | | | | | | | FAD-dependent oxidoreductase | | | | | | | |  |  |  |  |  |  |  |  |  |  |  |  |  |  |  |
| EFV53526.1 | | | | 0 | | | | | | | | | | | | | putative transporter, major facilitator family | | | | | | | | |  |  |  |  |  |  |  |  |  |  |  |  |  |  |  |
| EFV53535.1 | | | | 0 | | | | | | | | | | | | | fructose-bisphosphate aldolase class-I | | | | | | | | |  |  |  |  |  |  |  |  |  |  |  |  |  |  |  |
| EFV53546.1 | | | | 0 | | | | | | | | | | | | | conserved hypothetical protein | | | | | | | | |  |  |  |  |  |  |  |  |  |  |  |  |  |  |  |
| EFV53558.1 | | | | 0 | | | | | | | | | | | | | transcriptional enhancer factor TEF-4 | | | | | | | | |  |  |  |  |  |  |  |  |  |  |  |  |  |  |  |
| EFV53567.1 | | | | 0 | | | | | | | | | | | | | polyprenyl synthetase superfamily | | | | | | | | |  |  |  |  |  |  |  |  |  |  |  |  |  |  |  |
| EFV53576.1 | | | | 0 | | | | | | | | | | | | | HD domain-containing protein 2 | | | | | | | | |  |  |  |  |  |  |  |  |  |  |  |  |  |  |  |
| EFV53590.1 | | | | 0 | | | | | | | | | | | | | putative deoxyribose-phosphate aldolase | | | | | | | | |  |  |  |  |  |  |  |  |  |  |  |  |  |  |  |
| EFV53612.1 | | | | 0 | | | | | | | | | | | | | putative fibronectin type III domain protein | | | | | | | | |  |  |  |  |  |  |  |  |  |  |  |  |  |  |  |
| EFV53614.1 | | | | 0 | | | | | | | | | | | | | ribosomal-protein-alanine acetyltransferase | | | | | | | | |  |  |  |  |  |  |  |  |  |  |  |  |  |  |  |
| EFV53631.1 | | | | 0 | | | | | | | | | | | | | 5'-AMP-activated protein kinase catalytic subunit alpha-2 | | | | | | | | |  |  |  |  |  |  |  |  |  |  |  |  |  |  |  |
| EFV53682.1 | | | | 0 | | | | | | | | | | | | | Tau-tubulin kinase 1 | | | | | | | | |  |  |  |  |  |  |  |  |  |  |  |  |  |  |  |
| EFV53713.1 | | | | 0 | | | | | | | | | | | | | conserved hypothetical protein | | | | | | | | |  |  |  |  |  |  |  |  |  |  |  |  |  |  |  |
| EFV53721.1 | | | | 0 | | | | | | | | | | | | | formin 2 Domain protein | | | | | | | | |  |  |  |  |  |  |  |  |  |  |  |  |  |  |  |
| EFV53742.1 | | | | 0 | | | | | | | | | | | | | fatty oxidation complex, beta subunit | | | | | | | | |  |  |  |  |  |  |  |  |  |  |  |  |  |  |  |
| EFV53744.1 | | | | 0 | | | | | | | | | | | | | signal transduction-associated protein 1 | | | | | | | | |  |  |  |  |  |  |  |  |  |  |  |  |  |  |  |
| EFV53759.1 | | | | 0 | | | | | | | | | | | | | DnaJ protein subfamily B member 11 | | | | | | | | |  |  |  |  |  |  |  |  |  |  |  |  |  |  |  |
| EFV53808.1 | | | | 0 | | | | | | | | | | | | | putative YEATS domain-containing protein 4 | | | | | | | | |  |  |  |  |  |  |  |  |  |  |  |  |  |  |  |
| EFV53821.1 | | | | 0 | | | | | | | | | | | | | dystrobrevin-1 | | | | | | | | |  |  |  |  |  |  |  |  |  |  |  |  |  |  |  |
| EFV53824.1 | | | | 0 | | | | | | | | | | | | | conserved hypothetical protein | | | | | | | | |  |  |  |  |  |  |  |  |  |  |  |  |  |  |  |
| EFV53825.1 | | | | 0 | | | | | | | | | | | | | signal peptide peptidase family protein | | | | | | | | |  |  |  |  |  |  |  |  |  |  |  |  |  |  |  |
| EFV53848.1 | | | | 0 | | | | | | | | | | | | | putative ABC transporter, ATP-binding protein | | | | | | | | |  |  |  |  |  |  |  |  |  |  |  |  |  |  |  |
| EFV53862.1 | | | | 0 | | | | | | | | | | | | | EF hand domain containing protein | | | | | | | | |  |  |  |  |  |  |  |  |  |  |  |  |  |  |  |
| EFV53891.1 | | | | 0 | | | | | | | | | | | | | solute carrier family 35 member C2 | | | | | | | | |  |  |  |  |  |  |  |  |  |  |  |  |  |  |  |
| EFV53906.1 | | | | 0 | | | | | | | | | | | | | segment polarity protein | | | | | | | | |  |  |  |  |  |  |  |  |  |  |  |  |  |  |  |
| EFV53919.1 | | | | 0 | | | | | | | | | | | | | putative immunoglobulin domain protein | | | | | | | | |  |  |  |  |  |  |  |  |  |  |  |  |  |  |  |
| EFV53947.1 | | | | 0 | | | | | | | | | | | | | conserved hypothetical protein | | | | | | | | |  |  |  |  |  |  |  |  |  |  |  |  |  |  |  |
| EFV53956.1 | | | | 0 | | | | | | | | | | | | | chloride transporter, chloride channel family | | | | | | | | |  |  |  |  |  |  |  |  |  |  |  |  |  |  |  |
| EFV53958.1 | | | | 0 | | | | | | | | | | | | | vacuolar protein sorting-associated protein 28-like protein | | | | | | | | |  |  |  |  |  |  |  |  |  |  |  |  |  |  |  |
| EFV53959.1 | | | | 0 | | | | | | | | | | | | | tetratricopeptide repeat protein 35 | | | | | | | | |  |  |  |  |  |  |  |  |  |  |  |  |  |  |  |
| EFV53964.1 | | | | 0 | | | | | | | | | | | | | transcription factor HES-2 | | | | | | | | |  |  |  |  |  |  |  |  |  |  |  |  |  |  |  |
| EFV53966.1 | | | | 0 | | | | | | | | | | | | | cysteine protease ATG4B | | | | | | | | |  |  |  |  |  |  |  |  |  |  |  |  |  |  |  |
| EFV53987.1 | | | | 0 | | | | | | | | | | | | | putative RhoGEF domain protein | | | | | | | | |  |  |  |  |  |  |  |  |  |  |  |  |  |  |  |
| EFV54009.1 | | | | 0 | | | | | | | | | | | | | ribosomal protein L9, N- domain protein | | | | | | | | |  |  |  |  |  |  |  |  |  |  |  |  |  |  |  |
| EFV54010.1 | | | | 0 | | | | | | | | | | | | | general transcription factor 3C polypeptide 2 | | | | | | | | |  |  |  |  |  |  |  |  |  |  |  |  |  |  |  |
| EFV54039.1 | | | | 0 | | | | | | | | | | | | | putative JmjC domain-containing histone demethylation protein 2B | | | | | | | | |  |  |  |  |  |  |  |  |  |  |  |  |  |  |  |
| EFV54047.1 | | | | 0 | | | | | | | | | | | | | putative JmjC domain-containing histone demethylation protein 2B | | | | | | | | |  |  |  |  |  |  |  |  |  |  |  |  |  |  |  |
| EFV54055.1 | | | | 0 | | | | | | | | | | | | | glycosyl hydrolases family 38 N- domain protein | | | | | | | | |  |  |  |  |  |  |  |  |  |  |  |  |  |  |  |
| EFV54094.1 | | | | 0 | | | | | | | | | | | | | putative phospholipase D domain protein | | | | | | | | |  |  |  |  |  |  |  |  |  |  |  |  |  |  |  |
| EFV54106.1 | | | | 0 | | | | | | | | | | | | | conserved hypothetical protein | | | | | | | | |  |  |  |  |  |  |  |  |  |  |  |  |  |  |  |
| EFV54119.1 | | | | 0 | | | | | | | | | | | | | | vacuolar proton pump subunit G | | | | | | | | |  |  |  |  |  |  |  |  |  |  |  |  |  |  |
| EFV54136.1 | | | | 0 | | | | | | | | | | | | | | conserved hypothetical protein | | | | | | | | |  |  |  |  |  |  |  |  |  |  |  |  |  |  |
| EFV54156.1 | | | | 0 | | | | | | | | | | | | | | ADP-ribosylation factor GTPase-activating protein 3 | | | | | | | | |  |  |  |  |  |  |  |  |  |  |  |  |  |  |
| EFV54175.1 | | | | 0 | | | | | | | | | | | | | | putative G patch domain-containing protein 1-like protein | | | | | | | | |  |  |  |  |  |  |  |  |  |  |  |  |  |  |
| EFV54185.1 | | | | 0 | | | | | | | | | | | | | | putative PDZ domain protein | | | | | | | | |  |  |  |  |  |  |  |  |  |  |  |  |  |  |
| EFV54186.1 | | | | 0 | | | | | | | | | | | | | | putative patj-like protein | | | | | | | | |  |  |  |  |  |  |  |  |  |  |  |  |  |  |
| EFV54191.1 | | | | 0 | | | | | | | | | | | | | | cytochrome oxidase assembly protein | | | | | | | | |  |  |  |  |  |  |  |  |  |  |  |  |  |  |
| EFV54236.1 | | | | 0 | | | | | | | | | | | | | | calcipressin-3 | | | | | | | | |  |  |  |  |  |  |  |  |  |  |  |  |  |  |
| EFV54240.1 | | | | 0 | | | | | | | | | | | | | | period circadian protein | | | | | | | | |  |  |  |  |  |  |  |  |  |  |  |  |  |  |
| EFV54250.1 | | | | 0 | | | | | | | | | | | | | | conserved hypothetical protein | | | | | | | | |  |  |  |  |  |  |  |  |  |  |  |  |  |  |
| EFV54265.1 | | | | 0 | | | | | | | | | | | | | | conserved hypothetical protein | | | | | | | | |  |  |  |  |  |  |  |  |  |  |  |  |  |  |
| EFV54269.1 | | | | 0 | | | | | | | | | | | | | | acidic leucine-rich nuclear phosphoprotein 32 family member A | | | | | | | | |  |  |  |  |  |  |  |  |  |  |  |  |  |  |
| EFV54270.1 | | | | 0 | | | | | | | | | | | | | | iron-sulfur cluster assembly protein IscU | | | | | | | | |  |  |  |  |  |  |  |  |  |  |  |  |  |  |
| EFV54313.1 | | | | 0 | | | | | | | | | | | | | | protein arginine N-methyltransferase 5 | | | | | | | | |  |  |  |  |  |  |  |  |  |  |  |  |  |  |
| EFV54318.1 | | | | 0 | | | | | | | | | | | | | | protein transport protein Sec61 subunit beta | | | | | | | | |  |  |  |  |  |  |  |  |  |  |  |  |  |  |
| EFV54322.1 | | | | 0 | | | | | | | | | | | | | | 5'-3' exoribonuclease 2 | | | | | | | | |  |  |  |  |  |  |  |  |  |  |  |  |  |  |
| EFV54326.1 | | | | 0 | | | | | | | | | | | | | | putative surp module | | | | | | | | |  |  |  |  |  |  |  |  |  |  |  |  |  |  |
| EFV54353.1 | | | | 0 | | | | | | | | | | | | | | pleckstrin domain-containing family M member 1 | | | | | | | | |  |  |  |  |  |  |  |  |  |  |  |  |  |  |
| EFV54356.1 | | | | 0 | | | | | | | | | | | | | | L-lactate dehydrogenase | | | | | | | | |  |  |  |  |  |  |  |  |  |  |  |  |  |  |
| EFV54367.1 | | | | 0 | | | | | | | | | | | | | | putative ABC transporter, ATP-binding protein | | | | | | | | |  |  |  |  |  |  |  |  |  |  |  |  |  |  |
| EFV54370.1 | | | | 0 | | | | | | | | | | | | | | MAGUK p55 subfamily member 2 | | | | | | | | |  |  |  |  |  |  |  |  |  |  |  |  |  |  |
| EFV54372.1 | | | | 0 | | | | | | | | | | | | | | conserved hypothetical protein | | | | | | | | |  |  |  |  |  |  |  |  |  |  |  |  |  |  |
| EFV54416.1 | | | | 0 | | | | | | | | | | | | | | Sex muscle abnormal protein 5 | | | | | | | | |  |  |  |  |  |  |  |  |  |  |  |  |  |  |
| EFV54423.1 | | | | 0 | | | | | | | | | | | | | | putative ATP synthase F1, delta subunit | | | | | | | | |  |  |  |  |  |  |  |  |  |  |  |  |  |  |
| EFV54441.1 | | | | 0 | | | | | | | | | | | | | | hypothetical protein Tsp_07980 | | | | | | | | |  |  |  |  |  |  |  |  |  |  |  |  |  |  |
| EFV54444.1 | | | | 0 | | | | | | | | | | | | | | ATPase family protein | | | | | | | | |  |  |  |  |  |  |  |  |  |  |  |  |  |  |
| EFV54453.1 | | | | 0 | | | | | | | | | | | | | | putative SAM domain protein | | | | | | | | |  |  |  |  |  |  |  |  |  |  |  |  |  |  |
| EFV54462.1 | | | | 0 | | | | | | | | | | | | | | conserved hypothetical protein | | | | | | | | |  |  |  |  |  |  |  |  |  |  |  |  |  |  |
| EFV54486.1 | | | | 0 | | | | | | | | | | | | | | putative transcription factor RFX3 | | | | | | | | |  |  |  |  |  |  |  |  |  |  |  |  |  |  |
| EFV54496.1 | | | | 0 | | | | | | | | | | | | | | chloride intracellular channel exc-4 | | | | | | | | |  |  |  |  |  |  |  |  |  |  |  |  |  |  |
| EFV54561.1 | | | | 0 | | | | | | | | | | | | | | hypothetical protein Tsp_10113 | | | | | | | | |  |  |  |  |  |  |  |  |  |  |  |  |  |  |
| EFV54564.1 | | | | 0 | | | | | | | | | | | | | | putative ATP-dependent protease La | | | | | | | | |  |  |  |  |  |  |  |  |  |  |  |  |  |  |
| EFV54579.1 | | | | 0 | | | | | | | | | | | | | | UDP-N-acetylglucosamine transporter | | | | | | | | |  |  |  |  |  |  |  |  |  |  |  |  |  |  |
| EFV54618.1 | | | | 0 | | | | | | | | | | | | | | conserved hypothetical protein | | | | | | | | |  |  |  |  |  |  |  |  |  |  |  |  |  |  |
| EFV54634.1 | | | | 0 | | | | | | | | | | | | | | UDP-glucose 4-epimerase | | | | | | | | |  |  |  |  |  |  |  |  |  |  |  |  |  |  |
| EFV54635.1 | | | | 0 | | | | | | | | | | | | | | acyl-protein thioesterase 1 | | | | | | | | |  |  |  |  |  |  |  |  |  |  |  |  |  |  |
| EFV54640.1 | | | | 0 | | | | | | | | | | | | | | NADH dehydrogenase [ubiquinone] 1 alpha subcomplex subunit 13 | | | | | | | | |  |  |  |  |  |  |  |  |  |  |  |  |  |  |
| EFV54682.1 | | | | 0 | | | | | | | | | | | | | | DEAD-box helicase 1 | | | | | | | | |  |  |  |  |  |  |  |  |  |  |  |  |  |  |
| EFV54694.1 | | | | | 0 | | | | | | | | | | | | | | putative HEAT repeat-containing domain protein | | | | | | | | |  |  |  |  |  |  |  |  |  |  |  |  |  |
| EFV54700.1 | | | | | 0 | | | | | | | | | | | | | | PurA ssDNA and RNA-binding protein | | | | | | | | |  |  |  |  |  |  |  |  |  |  |  |  |  |
| EFV54702.1 | | | | | 0 | | | | | | | | | | | | | | histone arginine demethylase JMJD6 | | | | | | | | |  |  |  |  |  |  |  |  |  |  |  |  |  |
| EFV54752.1 | | | | | 0 | | | | | | | | | | | | | | conserved hypothetical protein | | | | | | | | |  |  |  |  |  |  |  |  |  |  |  |  |  |
| EFV54757.1 | | | | | 0 | | | | | | | | | | | | | | NEDD8-activating enzyme E1 catalytic subunit | | | | | | | | |  |  |  |  |  |  |  |  |  |  |  |  |  |
| EFV54761.1 | | | | | 0 | | | | | | | | | | | | | | heat shock protein beta-1 | | | | | | | | |  |  |  |  |  |  |  |  |  |  |  |  |  |
| EFV54762.1 | | | | | 0 | | | | | | | | | | | | | | zinc finger RAD18 domain-containing protein | | | | | | | | |  |  |  |  |  |  |  |  |  |  |  |  |  |
| EFV54770.1 | | | | | 0 | | | | | | | | | | | | | | putative endomembrane protein 70 | | | | | | | | |  |  |  |  |  |  |  |  |  |  |  |  |  |
| EFV54782.1 | | | | | 0 | | | | | | | | | | | | | | venom protein 2 | | | | | | | | |  |  |  |  |  |  |  |  |  |  |  |  |  |
| EFV54784.1 | | | | | 0 | | | | | | | | | | | | | | transmembrane protein 110 | | | | | | | | |  |  |  |  |  |  |  |  |  |  |  |  |  |
| EFV54785.1 | | | | | 0 | | | | | | | | | | | | | | hypothetical protein Tsp_09250 | | | | | | | | |  |  |  |  |  |  |  |  |  |  |  |  |  |
| EFV54801.1 | | | | | 0 | | | | | | | | | | | | | | soluble calcium-activated nucleotidase 1 | | | | | | | | |  |  |  |  |  |  |  |  |  |  |  |  |  |
| EFV54803.1 | | | | | 0 | | | | | | | | | | | | | | AMOP domain protein | | | | | | | | |  |  |  |  |  |  |  |  |  |  |  |  |  |
| EFV54807.1 | | | | | 0 | | | | | | | | | | | | | | dolichyl-phosphate beta-glucosyltransferase | | | | | | | | |  |  |  |  |  |  |  |  |  |  |  |  |  |
| EFV54816.1 | | | | | 0 | | | | | | | | | | | | | | adenylate kinase isoenzyme 1 | | | | | | | | |  |  |  |  |  |  |  |  |  |  |  |  |  |
| EFV54837.1 | | | | | 0 | | | | | | | | | | | | | | putative cadherin domain protein, partial | | | | | | | | |  |  |  |  |  |  |  |  |  |  |  |  |  |
| EFV54853.1 | | | | | 0 | | | | | | | | | | | | | | cleavage and polyadenylation specificity factor subunit 2 | | | | | | | | |  |  |  |  |  |  |  |  |  |  |  |  |  |
| EFV54860.1 | | | | | 0 | | | | | | | | | | | | | | zinc finger protein | | | | | | | | |  |  |  |  |  |  |  |  |  |  |  |  |  |
| EFV54863.1 | | | | | 0 | | | | | | | | | | | | | | hypothetical protein Tsp_09193 | | | | | | | | |  |  |  |  |  |  |  |  |  |  |  |  |  |
| EFV54875.1 | | | | | 0 | | | | | | | | | | | | | | putative cadherin domain protein, partial | | | | | | | | |  |  |  |  |  |  |  |  |  |  |  |  |  |
| EFV54878.1 | | | | | 0 | | | | | | | | | | | | | | arsenite-resistance protein 2-like protein | | | | | | | | |  |  |  |  |  |  |  |  |  |  |  |  |  |
| EFV54909.1 | | | | | 0 | | | | | | | | | | | | | | protein tyrosine phosphatase domain-containing protein 1 | | | | | | | | |  |  |  |  |  |  |  |  |  |  |  |  |  |
| EFV54917.1 | | | | | 0 | | | | | | | | | | | | | | dimethylaniline monooxygenase [N-oxide-forming] 4 | | | | | | | | |  |  |  |  |  |  |  |  |  |  |  |  |  |
| EFV54939.1 | | | | | 0 | | | | | | | | | | | | | | transcription initiation factor TFIID subunit 13 | | | | | | | | |  |  |  |  |  |  |  |  |  |  |  |  |  |
| EFV54945.1 | | | | | 0 | | | | | | | | | | | | | | putative IPT/TIG domain protein, partial | | | | | | | | |  |  |  |  |  |  |  |  |  |  |  |  |  |
| EFV54949.1 | | | | | 0 | | | | | | | | | | | | | | Yes-associated protein | | | | | | | | |  |  |  |  |  |  |  |  |  |  |  |  |  |
| EFV54952.1 | | | | | 0 | | | | | | | | | | | | | | 60S ribosomal protein L3 | | | | | | | | |  |  |  |  |  |  |  |  |  |  |  |  |  |
| EFV55010.1 | | | | | 0 | | | | | | | | | | | | | | putative glycerophosphodiester phosphodiesterase 5 | | | | | | | | |  |  |  |  |  |  |  |  |  |  |  |  |  |
| EFV55058.1 | | | | | 0 | | | | | | | | | | | | | | hypothetical protein Tsp_08369 | | | | | | | | |  |  |  |  |  |  |  |  |  |  |  |  |  |
| EFV55068.1 | | | | | 0 | | | | | | | | | | | | | | malate dehydrogenase | | | | | | | | |  |  |  |  |  |  |  |  |  |  |  |  |  |
| EFV55075.1 | | | | | 0 | | | | | | | | | | | | | | putative hexokinase HKDC1 | | | | | | | | |  |  |  |  |  |  |  |  |  |  |  |  |  |
| EFV55076.1 | | | | | 0 | | | | | | | | | | | | | | N- acetyltransferase A complex catalytic subunit Ard1 | | | | | | | | |  |  |  |  |  |  |  |  |  |  |  |  |  |
| EFV55077.1 | | | | | 0 | | | | | | | | | | | | | | Two pore potassium channel protein sup-9 | | | | | | | | |  |  |  |  |  |  |  |  |  |  |  |  |  |
| EFV55099.1 | | | | | 0 | | | | | | | | | | | | | | sodium-independent organic anion transporter family protein | | | | | | | | |  |  |  |  |  |  |  |  |  |  |  |  |  |
| EFV55194.1 | | | | | 0 | | | | | | | | | | | | | | WD repeat-containing protein 74 | | | | | | | | |  |  |  |  |  |  |  |  |  |  |  |  |  |
| EFV55197.1 | | | | | 0 | | | | | | | | | | | | | | cell differentiation protein RCD1-like protein | | | | | | | | |  |  |  |  |  |  |  |  |  |  |  |  |  |
| EFV55199.1 | | | | | 0 | | | | | | | | | | | | | | protein phosphatase inhibitor 2 | | | | | | | | |  |  |  |  |  |  |  |  |  |  |  |  |  |
| EFV55204.1 | | | | | 0 | | | | | | | | | | | | | | 60S ribosomal protein L22, partial | | | | | | | | |  |  |  |  |  |  |  |  |  |  |  |  |  |
| EFV55214.1 | | | | | 0 | | | | | | | | | | | | | | putative WH1 domain protein | | | | | | | | |  |  |  |  |  |  |  |  |  |  |  |  |  |
| EFV55221.1 | | | | | 0 | | | | | | | | | | | | | | eukaryotic translation initiation factor 3 subunit E, partial | | | | | | | | |  |  |  |  |  |  |  |  |  |  |  |  |  |
| EFV55230.1 | | | | | 0 | | | | | | | | | | | | | | serine/threonine-protein phosphatase 2A catalytic subunit beta isoform | | | | | | | | |  |  |  |  |  |  |  |  |  |  |  |  |  |
| EFV55242.1 | | | | | 0 | | | | | | | | | | | | | | WD repeat-containing protein 20 | | | | | | | | | |  |  |  |  |  |  |  |  |  |  |  |  |
| EFV55275.1 | | | | | 0 | | | | | | | | | | | | | | calcium-dependent secretion activator 1 | | | | | | | | | |  |  |  |  |  |  |  |  |  |  |  |  |
| EFV55280.1 | | | | | 0 | | | | | | | | | | | | | | conserved hypothetical protein | | | | | | | | | |  |  |  |  |  |  |  |  |  |  |  |  |
| EFV55284.1 | | | | | 0 | | | | | | | | | | | | | | putative alpha/beta hydrolase fold protein | | | | | | | | | |  |  |  |  |  |  |  |  |  |  |  |  |
| EFV55296.1 | | | | | 0 | | | | | | | | | | | | | | 40S ribosomal protein S19S | | | | | | | | | |  |  |  |  |  |  |  |  |  |  |  |  |
| EFV55297.1 | | | | | 0 | | | | | | | | | | | | | | exostosin-1 | | | | | | | | | |  |  |  |  |  |  |  |  |  |  |  |  |
| EFV55335.1 | | | | | 0 | | | | | | | | | | | | | | putative HEAT repeat-containing domain protein | | | | | | | | | |  |  |  |  |  |  |  |  |  |  |  |  |
| EFV55337.1 | | | | | 0 | | | | | | | | | | | | | | transcriptional adapter 2-beta | | | | | | | | | |  |  |  |  |  |  |  |  |  |  |  |  |
| EFV55339.1 | | | | | 0 | | | | | | | | | | | | | | NUC153 domain protein | | | | | | | | | |  |  |  |  |  |  |  |  |  |  |  |  |
| EFV55352.1 | | | | | 0 | | | | | | | | | | | | | | queuine tRNA-ribosyltransferase domain-containing protein 1 | | | | | | | | | |  |  |  |  |  |  |  |  |  |  |  |  |
| EFV55357.1 | | | | | 0 | | | | | | | | | | | | | | molybdenum cofactor synthesis protein 3 | | | | | | | | | |  |  |  |  |  |  |  |  |  |  |  |  |
| EFV55358.1 | | | | | 0 | | | | | | | | | | | | | | putative BTB/POZ domain protein | | | | | | | | | |  |  |  |  |  |  |  |  |  |  |  |  |
| EFV55362.1 | | | | | 0 | | | | | | | | | | | | | | transmembrane anterior posterior transformation protein 1 | | | | | | | | | |  |  |  |  |  |  |  |  |  |  |  |  |
| EFV55364.1 | | | | | 0 | | | | | | | | | | | | | | elongation factor 1-gamma | | | | | | | | | |  |  |  |  |  |  |  |  |  |  |  |  |
| EFV55365.1 | | | | | 0 | | | | | | | | | | | | | | nicotinamide N-methyltransferase | | | | | | | | | |  |  |  |  |  |  |  |  |  |  |  |  |
| EFV55402.1 | | | | | 0 | | | | | | | | | | | | | | vacuolar sorting protein 9 domain protein | | | | | | | | | |  |  |  |  |  |  |  |  |  |  |  |  |
| EFV55432.1 | | | | | 0 | | | | | | | | | | | | | | putative arrestin domain protein | | | | | | | | | |  |  |  |  |  |  |  |  |  |  |  |  |
| EFV55455.1 | | | | | 0 | | | | | | | | | | | | | | hypothetical protein Tsp_04191 | | | | | | | | | |  |  |  |  |  |  |  |  |  |  |  |  |
| EFV55460.1 | | | | | 0 | | | | | | | | | | | | | | methionine aminopeptidase 2 | | | | | | | | | |  |  |  |  |  |  |  |  |  |  |  |  |
| EFV55488.1 | | | | | 0 | | | | | | | | | | | | | | SOX-1 protein | | | | | | | | | |  |  |  |  |  |  |  |  |  |  |  |  |
| EFV55493.1 | | | | | 0 | | | | | | | | | | | | | | vacuolar protein-sorting-associated protein 36 | | | | | | | | | |  |  |  |  |  |  |  |  |  |  |  |  |
| EFV55517.1 | | | | | 0 | | | | | | | | | | | | | | f-actin-capping protein subunit alpha | | | | | | | | | |  |  |  |  |  |  |  |  |  |  |  |  |
| EFV55521.1 | | | | | 0 | | | | | | | | | | | | | | trafficking protein particle complex subunit 4 | | | | | | | | | |  |  |  |  |  |  |  |  |  |  |  |  |
| EFV55525.1 | | | | | 0 | | | | | | | | | | | | | | selenoprotein P, N region | | | | | | | | | |  |  |  |  |  |  |  |  |  |  |  |  |
| EFV55539.1 | | | | | 0 | | | | | | | | | | | | | | O-phosphoseryl-tRNA(Sec) selenium transferase | | | | | | | | | |  |  |  |  |  |  |  |  |  |  |  |  |
| EFV55553.1 | | | | | 0 | | | | | | | | | | | | | | putative fibronectin type III domain protein | | | | | | | | | |  |  |  |  |  |  |  |  |  |  |  |  |
| EFV55581.1 | | | | | 0 | | | | | | | | | | | | | | putative thrombospondin type 1 domain protein | | | | | | | | | |  |  |  |  |  |  |  |  |  |  |  |  |
| EFV55594.1 | | | | | 0 | | | | | | | | | | | | | | tRNA (5-methylaminomethyl-2-thiouridylate)-methyltransferase | | | | | | | | | |  |  |  |  |  |  |  |  |  |  |  |  |
| EFV55601.1 | | | | | 0 | | | | | | | | | | | | | | 26S proteasome non-ATPase regulatory subunit 1 | | | | | | | | | |  |  |  |  |  |  |  |  |  |  |  |  |
| EFV55609.1 | | | | | 0 | | | | | | | | | | | | | | UNC45 protein | | | | | | | | | |  |  |  |  |  |  |  |  |  |  |  |  |
| EFV55613.1 | | | | | 0 | | | | | | | | | | | | | | DNA repair protein Rad4 | | | | | | | | | |  |  |  |  |  |  |  |  |  |  |  |  |
| EFV55624.1 | | | | | 0 | | | | | | | | | | | | | | 7 transmembrane receptor | | | | | | | | | |  |  |  |  |  |  |  |  |  |  |  |  |
| EFV55625.1 | | | | | 0 | | | | | | | | | | | | | | polyribonucleotide nucleotidyltransferase | | | | | | | | | |  |  |  |  |  |  |  |  |  |  |  |  |
| EFV55633.1 | | | | | 0 | | | | | | | | | | | | | | mannose-6-phosphate isomerase | | | | | | | | | |  |  |  |  |  |  |  |  |  |  |  |  |
| EFV55636.1 | | | | | 0 | | | | | | | | | | | | | | EB module family protein, partial | | | | | | | | | |  |  |  |  |  |  |  |  |  |  |  |  |
| EFV55653.1 | | | | | 0 | | | | | | | | | | | | | | isoleucyl-tRNA synthetase | | | | | | | | | |  |  |  |  |  |  |  |  |  |  |  |  |
| EFV55660.1 | | | | | 0 | | | | | | | | | | | | | | ribosomal protein S16 | | | | | | | | | |  |  |  |  |  |  |  |  |  |  |  |  |
| EFV55663.1 | | | | | 0 | | | | | | | | | | | | | | Rho guanine nucleotide exchange factor 7 | | | | | | | | | |  |  |  |  |  |  |  |  |  |  |  |  |
| EFV55675.1 | | | | | 0 | | | | | | | | | | | | | | GMP synthase, partial | | | | | | | | | |  |  |  |  |  |  |  |  |  |  |  |  |
| EFV55688.1 | | | | | 0 | | | | | | | | | | | | | | Pre-mRNA-processing factor 6 | | | | | | | | | |  |  |  |  |  |  |  |  |  |  |  |  |
| EFV55707.1 | | | | | 0 | | | | | | | | | | | | | | domain protein, SNF2 family | | | | | | | | | |  |  |  |  |  |  |  |  |  |  |  |  |
| EFV55721.1 | | | 0 | | | | | | | | | | | | | | | | diacylglycerol O-acyltransferase 2 | | | | | | | | | | |  |  |  |  |  |  |  |  |  |  |  |
| EFV55726.1 | | | 0 | | | | | | | | | | | | | | | | hypothetical protein Tsp_03915 | | | | | | | | | | |  |  |  |  |  |  |  |  |  |  |  |
| EFV55768.1 | | | 0 | | | | | | | | | | | | | | | | 60S ribosomal export protein Nmd3 | | | | | | | | | | |  |  |  |  |  |  |  |  |  |  |  |
| EFV55806.1 | | | 0 | | | | | | | | | | | | | | | | pyrroline-5-carboxylate reductase | | | | | | | | | | |  |  |  |  |  |  |  |  |  |  |  |
| EFV55811.1 | | | 0 | | | | | | | | | | | | | | | | putative sushi domain protein | | | | | | | | | | |  |  |  |  |  |  |  |  |  |  |  |
| EFV55869.1 | | | 0 | | | | | | | | | | | | | | | | formin-binding protein 1 | | | | | | | | | | |  |  |  |  |  |  |  |  |  |  |  |
| EFV55895.1 | | | 0 | | | | | | | | | | | | | | | | FAD synthetase | | | | | | | | | | |  |  |  |  |  |  |  |  |  |  |  |
| EFV55906.1 | | | 0 | | | | | | | | | | | | | | | | sphingolipid delta(4)-desaturase DES1 | | | | | | | | | | |  |  |  |  |  |  |  |  |  |  |  |
| EFV55907.1 | | | 0 | | | | | | | | | | | | | | | | alpha-soluble NSF attachment protein | | | | | | | | | | |  |  |  |  |  |  |  |  |  |  |  |
| EFV55914.1 | | | 0 | | | | | | | | | | | | | | | | 40S ribosomal protein S4 | | | | | | | | | | |  |  |  |  |  |  |  |  |  |  |  |
| EFV55953.1 | | | 0 | | | | | | | | | | | | | | | | F-box/SPRY domain-containing protein 1 | | | | | | | | | | |  |  |  |  |  |  |  |  |  |  |  |
| EFV55962.1 | | | 0 | | | | | | | | | | | | | | | | conserved hypothetical protein | | | | | | | | | | |  |  |  |  |  |  |  |  |  |  |  |
| EFV55989.1 | | | 0 | | | | | | | | | | | | | | | | DNA cross-link repair 1A protein | | | | | | | | | | |  |  |  |  |  |  |  |  |  |  |  |
| EFV55995.1 | | | 0 | | | | | | | | | | | | | | | | glutamate--tRNA ligase | | | | | | | | | | |  |  |  |  |  |  |  |  |  |  |  |
| EFV56008.1 | | | 0 | | | | | | | | | | | | | | | | serine/threonine- protein phosphatase regulatory subunit gamma | | | | | | | | | | |  |  |  |  |  |  |  |  |  |  |  |
| EFV56044.1 | | | 0 | | | | | | | | | | | | | | | | fanconi anemia group M protein | | | | | | | | | | |  |  |  |  |  |  |  |  |  |  |  |
| EFV56061.1 | | | 0 | | | | | | | | | | | | | | | | lipid phosphate phosphohydrolase 1 | | | | | | | | | | |  |  |  |  |  |  |  |  |  |  |  |
| EFV56074.1 | | | 0 | | | | | | | | | | | | | | | | eukaryotic translation initiation factor 4H | | | | | | | | | | |  |  |  |  |  |  |  |  |  |  |  |
| EFV56075.1 | | | 0 | | | | | | | | | | | | | | | | putative ABC transporter, ATP-binding protein | | | | | | | | | | |  |  |  |  |  |  |  |  |  |  |  |
| EFV56076.1 | | | 0 | | | | | | | | | | | | | | | | syntaxin-16 | | | | | | | | | | |  |  |  |  |  |  |  |  |  |  |  |
| EFV56082.1 | | | 0 | | | | | | | | | | | | | | | | barrier-to-autointegration factor 1 | | | | | | | | | | |  |  |  |  |  |  |  |  |  |  |  |
| EFV56098.1 | | | 0 | | | | | | | | | | | | | | | | g patch domain and KOW motif proteins-containing protein | | | | | | | | | | |  |  |  |  |  |  |  |  |  |  |  |
| EFV56101.1 | | | 0 | | | | | | | | | | | | | | | | putative semaphorin-5B | | | | | | | | | | |  |  |  |  |  |  |  |  |  |  |  |
| EFV56108.1 | | | 0 | | | | | | | | | | | | | | | | putative integrin alpha pat-2 | | | | | | | | | | |  |  |  |  |  |  |  |  |  |  |  |
| EFV56114.1 | | | 0 | | | | | | | | | | | | | | | | phosphomevalonate kinase | | | | | | | | | | |  |  |  |  |  |  |  |  |  |  |  |
| EFV56115.1 | | | 0 | | | | | | | | | | | | | | | | putative tetratricopeptide repeat-containing domain protein | | | | | | | | | | |  |  |  |  |  |  |  |  |  |  |  |
| EFV56156.1 | | | 0 | | | | | | | | | | | | | | | | conserved hypothetical protein | | | | | | | | | | |  |  |  |  |  |  |  |  |  |  |  |
| EFV56185.1 | | | 0 | | | | | | | | | | | | | | | | domain protein, SNF2 family | | | | | | | | | | |  |  |  |  |  |  |  |  |  |  |  |
| EFV56187.1 | | | 0 | | | | | | | | | | | | | | | | DENN domain-containing protein 1B | | | | | | | | | | |  |  |  |  |  |  |  |  |  |  |  |
| EFV56197.1 | | | 0 | | | | | | | | | | | | | | | | acyl CoA binding protein | | | | | | | | | | |  |  |  |  |  |  |  |  |  |  |  |
| EFV56231.1 | | | 0 | | | | | | | | | | | | | | | | putative IQ calmodulin-binding motif protein | | | | | | | | | | |  |  |  |  |  |  |  |  |  |  |  |
| EFV56237.1 | | | 0 | | | | | | | | | | | | | | | | 7 transmembrane receptor | | | | | | | | | | |  |  |  |  |  |  |  |  |  |  |  |
| EFV56277.1 | | | 0 | | | | | | | | | | | | | | | | Polyadenylate-binding protein 2 | | | | | | | | | | |  |  |  |  |  |  |  |  |  |  |  |
| EFV56306.1 | | | 0 | | | | | | | | | | | | | | | | protein phosphatase 1 regulatory subunit 7 | | | | | | | | | | |  |  |  |  |  |  |  |  |  |  |  |
| EFV56314.1 | | | 0 | | | | | | | | | | | | | | | | 2',3'-cyclic nucleotide 3'-phosphodiesterase family protein | | | | | | | | | | |  |  |  |  |  |  |  |  |  |  |  |
| EFV56324.1 | | | 0 | | | | | | | | | | | | | | | | cleavage and polyadenylation specificity factor protein subunit 4 | | | | | | | | | | |  |  |  |  |  |  |  |  |  |  |  |
| EFV56325.1 | | | 0 | | | | | | | | | | | | | | | | hypothetical protein Tsp_06395 | | | | | | | | | | |  |  |  |  |  |  |  |  |  |  |  |
| EFV56335.1 | | | 0 | | | | | | | | | | | | | | | | TRAP240 family protein | | | | | | | | | | |  |  |  |  |  |  |  |  |  |  |  |
| EFV56346.1 | | | 0 | | | | | | | | | | | | | | | | cystathionine beta-lyase | | | | | | | | | | |  |  |  |  |  |  |  |  |  |  |  |
| EFV56368.1 | | | | 0 | | | | | | | | | | | | | | | phosphoprotein phosphatase 1, partial | | | | | | | | | | | |  |  |  |  |  |  |  |  |  |  |
| EFV56377.1 | | | | 0 | | | | | | | | | | | | | | | putative LIM domain protein | | | | | | | | | | | |  |  |  |  |  |  |  |  |  |  |
| EFV56401.1 | | | | 0 | | | | | | | | | | | | | | | asparaginyl-tRNA synthetase, cytoplasmic | | | | | | | | | | | |  |  |  |  |  |  |  |  |  |  |
| EFV56407.1 | | | | 0 | | | | | | | | | | | | | | | 40S ribosomal protein S25 | | | | | | | | | | | |  |  |  |  |  |  |  |  |  |  |
| EFV56413.1 | | | | 0 | | | | | | | | | | | | | | | synaptic vesicular amine transporter | | | | | | | | | | | |  |  |  |  |  |  |  |  |  |  |
| EFV56432.1 | | | | 0 | | | | | | | | | | | | | | | ribosomal protein L11 | | | | | | | | | | | |  |  |  |  |  |  |  |  |  |  |
| EFV56439.1 | | | | 0 | | | | | | | | | | | | | | | phospholipase, patatin family | | | | | | | | | | | |  |  |  |  |  |  |  |  |  |  |
| EFV56450.1 | | | | 0 | | | | | | | | | | | | | | | GTP-binding ADP-ribosylation factor | | | | | | | | | | | |  |  |  |  |  |  |  |  |  |  |
| EFV56454.1 | | | | 0 | | | | | | | | | | | | | | | conserved hypothetical protein | | | | | | | | | | | |  |  |  |  |  |  |  |  |  |  |
| EFV56461.1 | | | | 0 | | | | | | | | | | | | | | | activating signal cointegrator 1 | | | | | | | | | | | |  |  |  |  |  |  |  |  |  |  |
| EFV56469.1 | | | | 0 | | | | | | | | | | | | | | | chromatin regulator subfamily B member 1 | | | | | | | | | | | |  |  |  |  |  |  |  |  |  |  |
| EFV56485.1 | | | | 0 | | | | | | | | | | | | | | | CTP synthase | | | | | | | | | | | |  |  |  |  |  |  |  |  |  |  |
| EFV56504.1 | | | | 0 | | | | | | | | | | | | | | | DNA polymerase subunit delta-2 | | | | | | | | | | | |  |  |  |  |  |  |  |  |  |  |
| EFV56516.1 | | | | 0 | | | | | | | | | | | | | | | putative phosphatidylinositol 3-kinase regulatory subunit alpha | | | | | | | | | | | |  |  |  |  |  |  |  |  |  |  |
| EFV56520.1 | | | | 0 | | | | | | | | | | | | | | | excitatory amino acid transporter 1 | | | | | | | | | | | |  |  |  |  |  |  |  |  |  |  |
| EFV56529.1 | | | | 0 | | | | | | | | | | | | | | | TBC1 domain family member 23 | | | | | | | | | | | |  |  |  |  |  |  |  |  |  |  |
| EFV56531.1 | | | | 0 | | | | | | | | | | | | | | | 5-hydroxytryptamine receptor 2C | | | | | | | | | | | |  |  |  |  |  |  |  |  |  |  |
| EFV56545.1 | | | | 0 | | | | | | | | | | | | | | | putative malonyl CoA-acyl carrier protein transacylase | | | | | | | | | | | |  |  |  |  |  |  |  |  |  |  |
| EFV56570.1 | | | | 0 | | | | | | | | | | | | | | | N(2),N(2)-dimethylguanosine tRNA methyltransferase | | | | | | | | | | | |  |  |  |  |  |  |  |  |  |  |
| EFV56588.1 | | | | 0 | | | | | | | | | | | | | | | phosphoglycerate mutase, 2,3-bisphosphoglycerate-independent | | | | | | | | | | | |  |  |  |  |  |  |  |  |  |  |
| EFV56619.1 | | | | 0 | | | | | | | | | | | | | | | putative protein phosphatase 2C | | | | | | | | | | | |  |  |  |  |  |  |  |  |  |  |
| EFV56639.1 | | | | 0 | | | | | | | | | | | | | | | small nuclear ribonucleoprotein F | | | | | | | | | | | |  |  |  |  |  |  |  |  |  |  |
| EFV56656.1 | | | | 0 | | | | | | | | | | | | | | | diphthamide biosynthesis protein 1 | | | | | | | | | | | |  |  |  |  |  |  |  |  |  |  |
| EFV56674.1 | | | | 0 | | | | | | | | | | | | | | | multifunctional protein ADE2, partial | | | | | | | | | | | |  |  |  |  |  |  |  |  |  |  |
| EFV56681.1 | | | | 0 | | | | | | | | | | | | | | | bestrophin-1 | | | | | | | | | | | |  |  |  |  |  |  |  |  |  |  |
| EFV56726.1 | | | | 0 | | | | | | | | | | | | | | | 40S ribosomal protein S15 | | | | | | | | | | | |  |  |  |  |  |  |  |  |  |  |
| EFV56739.1 | | | | 0 | | | | | | | | | | | | | | | PAB-dependent poly(A)-specific ribonuclease subunit 2 | | | | | | | | | | | |  |  |  |  |  |  |  |  |  |  |
| EFV56743.1 | | | | 0 | | | | | | | | | | | | | | | polypeptide N-acetylgalactosaminyltransferase 3 | | | | | | | | | | | |  |  |  |  |  |  |  |  |  |  |
| EFV56788.1 | | | | 0 | | | | | | | | | | | | | | | homeobox protein SAX-1 | | | | | | | | | | | |  |  |  |  |  |  |  |  |  |  |
| EFV56795.1 | | | | 0 | | | | | | | | | | | | | | | NDT80 / PhoG like DNA-binding family protein | | | | | | | | | | | |  |  |  |  |  |  |  |  |  |  |
| EFV56797.1 | | | | 0 | | | | | | | | | | | | | | | conserved hypothetical protein | | | | | | | | | | | |  |  |  |  |  |  |  |  |  |  |
| EFV56841.1 | | | | 0 | | | | | | | | | | | | | | | putative protein rogdi-like protein | | | | | | | | | | | |  |  |  |  |  |  |  |  |  |  |
| EFV56850.1 | | | | 0 | | | | | | | | | | | | | | | putative patched domain-containing protein 3 | | | | | | | | | | | |  |  |  |  |  |  |  |  |  |  |
| EFV56859.1 | | | | 0 | | | | | | | | | | | | | | | G2/mitotic-specific cyclin-B3 | | | | | | | | | | | |  |  |  |  |  |  |  |  |  |  |
| EFV56867.1 | | | | 0 | | | | | | | | | | | | | | | conserved hypothetical protein | | | | | | | | | | | |  |  |  |  |  |  |  |  |  |  |
| EFV56879.1 | | | | 0 | | | | | | | | | | | | | | | ATPase, AAA family | | | | | | | | | | | |  |  |  |  |  |  |  |  |  |  |
| EFV56884.1 | | | | 0 | | | | | | | | | | | | | | | putative LIM domain protein | | | | | | | | | | | |  |  |  |  |  |  |  |  |  |  |
| EFV56908.1 | | | | 0 | | | | | | | | | | | | | | | conserved hypothetical protein | | | | | | | | | | | |  |  |  |  |  |  |  |  |  |  |
| EFV56912.1 | | | | 0 | | | | | | | | | | | | | | | cyclin-dependent kinase inhibitor 2B-related protein | | | | | | | | | | | |  |  |  |  |  |  |  |  |  |  |
| EFV56915.1 | | | | 0 | | | | | | | | | | | | | | | hypothetical protein Tsp_02039 | | | | | | | | | | | |  |  |  |  |  |  |  |  |  |  |
| EFV56925.1 | | | | 0 | | | | | | | | | | | | | | | transcription factor Dp-1 | | | | | | | | | | | |  |  |  |  |  |  |  |  |  |  |
| EFV56926.1 | | | | 0 | | | | | | | | | | | | | | | dynein light chain 1, cytoplasmic | | | | | | | | | | | |  |  |  |  |  |  |  |  |  |  |
| EFV57011.1 | | | | 0 | | | | | | | | | | | | | | | intermediate filament protein ifa-1 | | | | | | | | | | | |  |  |  |  |  |  |  |  |  |  |
| EFV57040.1 | | | | | | 0 | | | | | | | | | | | | | | conserved hypothetical protein | | | | | | | | | | | |  |  |  |  |  |  |  |  |  |
| EFV57050.1 | | | | | | 0 | | | | | | | | | | | | | | ATPase, AAA family, partial | | | | | | | | | | | |  |  |  |  |  |  |  |  |  |
| EFV57057.1 | | | | | | 0 | | | | | | | | | | | | | | putative CRAL/TRIO domain protein | | | | | | | | | | | |  |  |  |  |  |  |  |  |  |
| EFV57071.1 | | | | | | 0 | | | | | | | | | | | | | | putative LIM and SH3 domain protein | | | | | | | | | | | |  |  |  |  |  |  |  |  |  |
| EFV57072.1 | | | | | | 0 | | | | | | | | | | | | | | conserved hypothetical protein | | | | | | | | | | | |  |  |  |  |  |  |  |  |  |
| EFV57091.1 | | | | | | 0 | | | | | | | | | | | | | | putative cAMP-regulated phosphoprotein 21 | | | | | | | | | | | |  |  |  |  |  |  |  |  |  |
| EFV57109.1 | | | | | | 0 | | | | | | | | | | | | | | conserved hypothetical protein | | | | | | | | | | | |  |  |  |  |  |  |  |  |  |
| EFV57160.1 | | | | | | 0 | | | | | | | | | | | | | | putative elongation factor Tu GTP binding domain protein | | | | | | | | | | | |  |  |  |  |  |  |  |  |  |
| EFV57196.1 | | | | | | 0 | | | | | | | | | | | | | | coronin-6 | | | | | | | | | | | |  |  |  |  |  |  |  |  |  |
| EFV57237.1 | | | | | | 0 | | | | | | | | | | | | | | tubulin-tyrosine ligase family protein | | | | | | | | | | | |  |  |  |  |  |  |  |  |  |
| EFV57260.1 | | | | | | 0 | | | | | | | | | | | | | | splicing factor, arginine/serine-rich 3 | | | | | | | | | | | |  |  |  |  |  |  |  |  |  |
| EFV57314.1 | | | | | | 0 | | | | | | | | | | | | | | DNA repair protein RAD51 | | | | | | | | | | | |  |  |  |  |  |  |  |  |  |
| EFV57316.1 | | | | | | 0 | | | | | | | | | | | | | | protein FRG1 | | | | | | | | | | | |  |  |  |  |  |  |  |  |  |
| EFV57325.1 | | | | | | 0 | | | | | | | | | | | | | | putative kinesin motor domain protein | | | | | | | | | | | |  |  |  |  |  |  |  |  |  |
| EFV57365.1 | | | | | | 0 | | | | | | | | | | | | | | pantothenate kinase 2 | | | | | | | | | | | |  |  |  |  |  |  |  |  |  |
| EFV57375.1 | | | | | | 0 | | | | | | | | | | | | | | serine protease inhibitor family protein | | | | | | | | | | | |  |  |  |  |  |  |  |  |  |
| EFV57379.1 | | | | | | 0 | | | | | | | | | | | | | | hypothetical protein Tsp_01546 | | | | | | | | | | | |  |  |  |  |  |  |  |  |  |
| EFV57402.1 | | | | | | 0 | | | | | | | | | | | | | | conserved hypothetical protein | | | | | | | | | | | |  |  |  |  |  |  |  |  |  |
| EFV57456.1 | | | | | | 0 | | | | | | | | | | | | | | CBF1-interacting corepressor | | | | | | | | | | | |  |  |  |  |  |  |  |  |  |
| EFV57458.1 | | | | | | 0 | | | | | | | | | | | | | | gamma-glutamyltranspeptidase 1 | | | | | | | | | | | |  |  |  |  |  |  |  |  |  |
| EFV57481.1 | | | | | | 0 | | | | | | | | | | | | | | conserved hypothetical protein | | | | | | | | | | | |  |  |  |  |  |  |  |  |  |
| EFV57482.1 | | | | | | 0 | | | | | | | | | | | | | | putative rRNA-processing protein EBP2 | | | | | | | | | | | |  |  |  |  |  |  |  |  |  |
| EFV57488.1 | | | | | | 0 | | | | | | | | | | | | | | acyl carrier protein | | | | | | | | | | | |  |  |  |  |  |  |  |  |  |
| EFV57489.1 | | | | | | 0 | | | | | | | | | | | | | | putative midasin | | | | | | | | | | | |  |  |  |  |  |  |  |  |  |
| EFV57521.1 | | | | | | 0 | | | | | | | | | | | | | | cytoplasmic FMR1-interacting protein | | | | | | | | | | | |  |  |  |  |  |  |  |  |  |
| EFV57535.1 | | | | | | 0 | | | | | | | | | | | | | | nuclear distribution protein NudE protein | | | | | | | | | | | |  |  |  |  |  |  |  |  |  |
| EFV57546.1 | | | | | | 0 | | | | | | | | | | | | | | hexosaminidase domain-containing protein | | | | | | | | | | | |  |  |  |  |  |  |  |  |  |
| EFV57562.1 | | | | | | 0 | | | | | | | | | | | | | | conserved hypothetical protein | | | | | | | | | | | |  |  |  |  |  |  |  |  |  |
| EFV57564.1 | | | | | | 0 | | | | | | | | | | | | | | mediator of RNA polymerase II transcription subunit 18 | | | | | | | | | | | |  |  |  |  |  |  |  |  |  |
| EFV57567.1 | | | | | | 0 | | | | | | | | | | | | | | E3 ubiquitin-protein ligase | | | | | | | | | | | |  |  |  |  |  |  |  |  |  |
| EFV57584.1 | | | | | | 0 | | | | | | | | | | | | | | 40S ribosomal protein S3a | | | | | | | | | | | |  |  |  |  |  |  |  |  |  |
| EFV57596.1 | | | | | | 0 | | | | | | | | | | | | | | glutamate synthase | | | | | | | | | | | |  |  |  |  |  |  |  |  |  |
| EFV57606.1 | | | | | | 0 | | | | | | | | | | | | | | conserved hypothetical protein | | | | | | | | | | | |  |  |  |  |  |  |  |  |  |
| EFV57612.1 | | | | | | 0 | | | | | | | | | | | | | | chromatin-remodeling complex ATPase chain Iswi, partial | | | | | | | | | | | |  |  |  |  |  |  |  |  |  |
| EFV57630.1 | | | | | | 0 | | | | | | | | | | | | | | putative nucleoredoxin | | | | | | | | | | | |  |  |  |  |  |  |  |  |  |
| EFV57649.1 | | | | | | 0 | | | | | | | | | | | | | | protein phosphatase PTC7-like protein | | | | | | | | | | | |  |  |  |  |  |  |  |  |  |
| EFV57652.1 | | | | | | 0 | | | | | | | | | | | | | | putative methionine synthase reductase | | | | | | | | | | | |  |  |  |  |  |  |  |  |  |
| EFV57653.1 | | | | | | 0 | | | | | | | | | | | | | | conserved hypothetical protein | | | | | | | | | | | |  |  |  |  |  |  |  |  |  |
| EFV57673.1 | | | | | | 0 | | | | | | | | | | | | | | Gut-specific cysteine proteinase | | | | | | | | | | | |  |  |  |  |  |  |  |  |  |
| EFV57695.1 | | | | | | 0 | | | | | | | | | | | | | | putative helix-loop-helix DNA-binding domain protein | | | | | | | | | | | |  |  |  |  |  |  |  |  |  |
| EFV57716.1 | | | | | | | 0 | | | | | | | | | | | | | | cytoChrome c oxidase subunit Va superfamily | | | | | | | | | | | |  |  |  |  |  |  |  |  |
| EFV57723.1 | | | | | | | 0 | | | | | | | | | | | | | | extracellular signal-regulated kinase 1 | | | | | | | | | | | |  |  |  |  |  |  |  |  |
| EFV57735.1 | | | | | | | 0 | | | | | | | | | | | | | | putative ribosomal protein S7e | | | | | | | | | | | |  |  |  |  |  |  |  |  |
| EFV57812.1 | | | | | | | 0 | | | | | | | | | | | | | | 7 transmembrane receptor | | | | | | | | | | | |  |  |  |  |  |  |  |  |
| EFV57823.1 | | | | | | | 0 | | | | | | | | | | | | | | protein-S-isoprenylcysteine O-methyltransferase | | | | | | | | | | | |  |  |  |  |  |  |  |  |
| EFV57838.1 | | | | | | | 0 | | | | | | | | | | | | | | nucleolar MIF4G domain-containing protein 1 | | | | | | | | | | | |  |  |  |  |  |  |  |  |
| EFV57840.1 | | | | | | | 0 | | | | | | | | | | | | | | putative PH domain protein | | | | | | | | | | | |  |  |  |  |  |  |  |  |
| EFV57870.1 | | | | | | | 0 | | | | | | | | | | | | | | cysteine and glycine-rich protein 1 | | | | | | | | | | | |  |  |  |  |  |  |  |  |
| EFV57903.1 | | | | | | | 0 | | | | | | | | | | | | | | proline--tRNA ligase | | | | | | | | | | | |  |  |  |  |  |  |  |  |
| EFV57904.1 | | | | | | | 0 | | | | | | | | | | | | | | CCR4-NOT transcription complex subunit 3 | | | | | | | | | | | |  |  |  |  |  |  |  |  |
| EFV57916.1 | | | | | | | 0 | | | | | | | | | | | | | | conserved hypothetical protein | | | | | | | | | | | |  |  |  |  |  |  |  |  |
| EFV57938.1 | | | | | | | 0 | | | | | | | | | | | | | | NADH dehydrogenase [ubiquinone] 1 beta subcomplex subunit 10 | | | | | | | | | | | |  |  |  |  |  |  |  |  |
| EFV57941.1 | | | | | | | 0 | | | | | | | | | | | | | | putative cadherin domain protein | | | | | | | | | | | |  |  |  |  |  |  |  |  |
| EFV57943.1 | | | | | | | 0 | | | | | | | | | | | | | | protein-tyrosine sulfotransferase A | | | | | | | | | | | |  |  |  |  |  |  |  |  |
| EFV57949.1 | | | | | | | 0 | | | | | | | | | | | | | | polymerase delta-interacting protein 2 | | | | | | | | | | | |  |  |  |  |  |  |  |  |
| EFV57966.1 | | | | | | | 0 | | | | | | | | | | | | | | protein BAT5 | | | | | | | | | | | |  |  |  |  |  |  |  |  |
| EFV57981.1 | | | | | | | 0 | | | | | | | | | | | | | | conserved hypothetical protein | | | | | | | | | | | |  |  |  |  |  |  |  |  |
| EFV57986.1 | | | | | | | 0 | | | | | | | | | | | | | | conserved hypothetical protein | | | | | | | | | | | |  |  |  |  |  |  |  |  |
| EFV57990.1 | | | | | | | 0 | | | | | | | | | | | | | | NADH dehydrogenase I, D subunit | | | | | | | | | | | |  |  |  |  |  |  |  |  |
| EFV58014.1 | | | | | | | 0 | | | | | | | | | | | | | | DNA topoisomerase 2-alpha | | | | | | | | | | | |  |  |  |  |  |  |  |  |
| EFV58028.1 | | | | | | | 0 | | | | | | | | | | | | | | glycine hydroxymethyltransferase | | | | | | | | | | | |  |  |  |  |  |  |  |  |
| EFV58065.1 | | | | | | | 0 | | | | | | | | | | | | | | nucleolar MIF4G domain-containing protein 1 | | | | | | | | | | | |  |  |  |  |  |  |  |  |
| EFV58078.1 | | | | | | | 0 | | | | | | | | | | | | | | tubulin alpha chain | | | | | | | | | | | |  |  |  |  |  |  |  |  |
| EFV58096.1 | | | | | | | 0 | | | | | | | | | | | | | | putative H/ACA ribonucleoprotein complex subunit 4 | | | | | | | | | | | |  |  |  |  |  |  |  |  |
| EFV58107.1 | | | | | | | 0 | | | | | | | | | | | | | | phosphorylase B kinase regulatory subunit alpha, liver isoform | | | | | | | | | | | |  |  |  |  |  |  |  |  |
| EFV58132.1 | | | | | | | 0 | | | | | | | | | | | | | | zinc finger protein ZPR1 | | | | | | | | | | | |  |  |  |  |  |  |  |  |
| EFV58141.1 | | | | | | | 0 | | | | | | | | | | | | | | zinc transporter 8 | | | | | | | | | | | |  |  |  |  |  |  |  |  |
| EFV58144.1 | | | | | | | 0 | | | | | | | | | | | | | | DnaJ protein subfamily C member 9 | | | | | | | | | | | |  |  |  |  |  |  |  |  |
| EFV58174.1 | | | | | | | 0 | | | | | | | | | | | | | | ribose 5-phosphate isomerase A | | | | | | | | | | | |  |  |  |  |  |  |  |  |
| EFV58197.1 | | | | | | | 0 | | | | | | | | | | | | | | fructose-1,6-bisphosphatase | | | | | | | | | | | |  |  |  |  |  |  |  |  |
| EFV58198.1 | | | | | | | 0 | | | | | | | | | | | | | | putative protein lin-9 | | | | | | | | | | | |  |  |  |  |  |  |  |  |
| EFV58199.1 | | | | | | | 0 | | | | | | | | | | | | | | splicing factor 3B subunit 1 | | | | | | | | | | | |  |  |  |  |  |  |  |  |
| EFV58201.1 | | | | | | | 0 | | | | | | | | | | | | | | arfaptin-1 | | | | | | | | | | | |  |  |  |  |  |  |  |  |
| EFV58204.1 | | | | | | | 0 | | | | | | | | | | | | | | putative CXXC zinc finger domain protein | | | | | | | | | | | |  |  |  |  |  |  |  |  |
| EFV58278.1 | | | | | | | | 0 | | | | | | | | | | | | | | nascent polypeptide-associated complex subunit alpha | | | | | | | | | | | |  |  |  |  |  |  |  |
| EFV58282.1 | | | | | | | | 0 | | | | | | | | | | | | | | 60S ribosomal protein L7a | | | | | | | | | | | |  |  |  |  |  |  |  |
| EFV58293.1 | | | | | | | | 0 | | | | | | | | | | | | | | ubiquitin fusion degradation protein 1-like protein | | | | | | | | | | | |  |  |  |  |  |  |  |
| EFV58303.1 | | | | | | | | 0 | | | | | | | | | | | | | | 60S ribosomal protein L18 | | | | | | | | | | | |  |  |  |  |  |  |  |
| EFV58307.1 | | | | | | | | 0 | | | | | | | | | | | | | | N-acetyl-D-glucosamine kinase | | | | | | | | | | | |  |  |  |  |  |  |  |
| EFV58309.1 | | | | | | | | 0 | | | | | | | | | | | | | | ATP-dependent RNA helicase abstrakt | | | | | | | | | | | |  |  |  |  |  |  |  |
| EFV58312.1 | | | | | | | | 0 | | | | | | | | | | | | | | 5-hydroxytryptamine receptor 1D | | | | | | | | | | | |  |  |  |  |  |  |  |
| EFV58323.1 | | | | | | | | 0 | | | | | | | | | | | | | | Gut-specific cysteine proteinase | | | | | | | | | | | |  |  |  |  |  |  |  |
| EFV58347.1 | | | | | | | | 0 | | | | | | | | | | | | | | transmembrane protein 120A | | | | | | | | | | | |  |  |  |  |  |  |  |
| EFV58359.1 | | | | | | | | 0 | | | | | | | | | | | | | | hypothetical protein Tsp_00570 | | | | | | | | | | | |  |  |  |  |  |  |  |
| EFV58367.1 | | | | | | | | 0 | | | | | | | | | | | | | | conserved hypothetical protein | | | | | | | | | | | |  |  |  |  |  |  |  |
| EFV58395.1 | | | | | | | | 0 | | | | | | | | | | | | | | fructosamine-3-kinase | | | | | | | | | | | |  |  |  |  |  |  |  |
| EFV58396.1 | | | | | | | | 0 | | | | | | | | | | | | | | cytochrome b5 domain-containing protein 2 | | | | | | | | | | | |  |  |  |  |  |  |  |
| EFV58401.1 | | | | | | | | 0 | | | | | | | | | | | | | | oxysterol-binding protein 2 | | | | | | | | | | | |  |  |  |  |  |  |  |
| EFV58402.1 | | | | | | | | 0 | | | | | | | | | | | | | | arginyl-tRNA synthetase, cytoplasmic | | | | | | | | | | | |  |  |  |  |  |  |  |
| EFV58431.1 | | | | | | | | 0 | | | | | | | | | | | | | | calumenin | | | | | | | | | | | |  |  |  |  |  |  |  |
| EFV58432.1 | | | | | | | | 0 | | | | | | | | | | | | | | Fip1 motif protein family protein | | | | | | | | | | | |  |  |  |  |  |  |  |
| EFV58464.1 | | | | | | | | 0 | | | | | | | | | | | | | | ribosome biogenesis protein RLP24 | | | | | | | | | | | |  |  |  |  |  |  |  |
| EFV58494.1 | | | | | | | | 0 | | | | | | | | | | | | | | signal peptidase complex catalytic subunit Sec11 | | | | | | | | | | | |  |  |  |  |  |  |  |
| EFV58515.1 | | | | | | | | 0 | | | | | | | | | | | | | | putative RanBP1 domain protein | | | | | | | | | | | |  |  |  |  |  |  |  |
| EFV58516.1 | | | | | | | | 0 | | | | | | | | | | | | | | putative RNA recognition motif protein | | | | | | | | | | | |  |  |  |  |  |  |  |
| EFV58537.1 | | | | | | | | 0 | | | | | | | | | | | | | | cyclin-Y | | | | | | | | | | | |  |  |  |  |  |  |  |
| EFV58549.1 | | | | | | | | 0 | | | | | | | | | | | | | | dimethyladenosine transferase | | | | | | | | | | | |  |  |  |  |  |  |  |
| EFV58554.1 | | | | | | | | 0 | | | | | | | | | | | | | | conserved hypothetical protein | | | | | | | | | | | |  |  |  |  |  |  |  |
| EFV58556.1 | | | | | | | | 0 | | | | | | | | | | | | | | deoxyhypusine hydroxylase | | | | | | | | | | | |  |  |  |  |  |  |  |
| EFV58557.1 | | | | | | | | 0 | | | | | | | | | | | | | | branched-chain-amino-acid transaminase | | | | | | | | | | | |  |  |  |  |  |  |  |
| EFV58568.1 | | | | | | | | 0 | | | | | | | | | | | | | | putative PDZ domain protein | | | | | | | | | | | |  |  |  |  |  |  |  |
| EFV58572.1 | | | | | | | | 0 | | | | | | | | | | | | | | glutaredoxin 4 | | | | | | | | | | | |  |  |  |  |  |  |  |
| EFV58588.1 | | | | | | | | 0 | | | | | | | | | | | | | | lonCoA ligase 5 | | | | | | | | | | | |  |  |  |  |  |  |  |
| EFV58633.1 | | | | | | | | 0 | | | | | | | | | | | | | | putative transcription initiation factor TFIID subunit 7 | | | | | | | | | | | |  |  |  |  |  |  |  |
| EFV58637.1 | | | | | | | | 0 | | | | | | | | | | | | | | conserved hypothetical protein | | | | | | | | | | | |  |  |  |  |  |  |  |
| EFV58637.1 | | | | | | | | 0 | | | | | | | | | | | | | | conserved hypothetical protein | | | | | | | | | | | |  |  |  |  |  |  |  |
| EFV58647.1 | | | | | | | | 0 | | | | | | | | | | | | | | timeless protein | | | | | | | | | | | |  |  |  |  |  |  |  |
| EFV58673.1 | | | | | | | | 0 | | | | | | | | | | | | | | putative zinc finger protein | | | | | | | | | | | |  |  |  |  |  |  |  |
| EFV58705.1 | | | | | | | | 0 | | | | | | | | | | | | | | small G protein signaling modulator 3-like protein | | | | | | | | | | | |  |  |  |  |  |  |  |
| EFV58714.1 | | | | | | | | 0 | | | | | | | | | | | | | | putative aconitate hydratase 2 | | | | | | | | | | | |  |  |  |  |  |  |  |
| EFV58736.1 | | | | | | | | 0 | | | | | | | | | | | | | | secretin receptor | | | | | | | | | | | |  |  |  |  |  |  |  |
| EFV58754.1 | | | | | | | | 0 | | | | | | | | | | | | | | nicotinamide mononucleotide adenylyltransferase 1 | | | | | | | | | | | |  |  |  |  |  |  |  |
| EFV58763.1 | | | | | | | | 0 | | | | | | | | | | | | | | methyltransferase domain protein | | | | | | | | | | | |  |  |  |  |  |  |  |
| EFV58768.1 | | | | | | | | | 0 | | | | | | | | | | | | secretin receptor | | | | | | | | | | | | | |  |  |  |  |  |  |
| EFV58812.1 | | | | | | | | | 0 | | | | | | | | | | | | protein Jade-1 | | | | | | | | | | | | | |  |  |  |  |  |  |
| EFV58823.1 | | | | | | | | | 0 | | | | | | | | | | | | glyco protein 3-alpha-L-fucosyltransferase A | | | | | | | | | | | | | |  |  |  |  |  |  |
| EFV58826.1 | | | | | | | | | 0 | | | | | | | | | | | | dual specificity mitogen-activated protein kinase kinase 4 | | | | | | | | | | | | | |  |  |  |  |  |  |
| EFV58830.1 | | | | | | | | | 0 | | | | | | | | | | | | phosphoribosyl pyrophosphate synthetase-associated protein 2 | | | | | | | | | | | | | |  |  |  |  |  |  |
| EFV58865.1 | | | | | | | | | 0 | | | | | | | | | | | | putative RNase3 domain protein | | | | | | | | | | | | | |  |  |  |  |  |  |
| EFV58871.1 | | | | | | | | | 0 | | | | | | | | | | | | PDZ domain protein | | | | | | | | | | | | | |  |  |  |  |  |  |
| EFV58892.1 | | | | | | | | | 0 | | | | | | | | | | | | putative snRNA-activating protein complex subunit 4 | | | | | | | | | | | | | |  |  |  |  |  |  |
| EFV58952.1 | | | | | | | | | 0 | | | | | | | | | | | | DnaJ protein | | | | | | | | | | | | | |  |  |  |  |  |  |
| EFV58953.1 | | | | | | | | | 0 | | | | | | | | | | | | growth/differentiation factor 8, partial | | | | | | | | | | | | | |  |  |  |  |  |  |
| EFV58965.1 | | | | | | | | | 0 | | | | | | | | | | | | chitin synthase domain protein | | | | | | | | | | | | | |  |  |  |  |  |  |
| EFV58983.1 | | | | | | | | | 0 | | | | | | | | | | | | ribulose-phosphate 3-epimerase | | | | | | | | | | | | | |  |  |  |  |  |  |
| EFV58989.1 | | | | | | | | | 0 | | | | | | | | | | | | aldose reductase | | | | | | | | | | | | | |  |  |  |  |  |  |
| EFV58998.1 | | | | | | | | | 0 | | | | | | | | | | | | helix-loop-helix protein 4 | | | | | | | | | | | | | |  |  |  |  |  |  |
| EFV59020.1 | | | | | | | | | 0 | | | | | | | | | | | | glycogen debranching enzyme | | | | | | | | | | | | | |  |  |  |  |  |  |
| EFV59024.1 | | | | | | | | | 0 | | | | | | | | | | | | small conductance calcium-activated potassium channel protein 3 | | | | | | | | | | | | | |  |  |  |  |  |  |
| EFV59029.1 | | | | | | | | | 0 | | | | | | | | | | | | putative translation initiation factor IF-2 | | | | | | | | | | | | | |  |  |  |  |  |  |
| EFV59032.1 | | | | | | | | | 0 | | | | | | | | | | | | GDP-mannose 4,6-dehydratase | | | | | | | | | | | | | |  |  |  |  |  |  |
| EFV59041.1 | | | | | | | | | 0 | | | | | | | | | | | | ribosomal protein S11 | | | | | | | | | | | | | |  |  |  |  |  |  |
| EFV59046.1 | | | | | | | | | 0 | | | | | | | | | | | | putative calcium binding EGF domain protein | | | | | | | | | | | | | |  |  |  |  |  |  |
| EFV59079.1 | | | | | | | | | 0 | | | | | | | | | | | | U4/U6 small nuclear ribonucleoprotein Prp3 | | | | | | | | | | | | | |  |  |  |  |  |  |
| EFV59121.1 | | | | | | | | | 0 | | | | | | | | | | | | conserved hypothetical protein | | | | | | | | | | | | | |  |  |  |  |  |  |
| EFV59124.1 | | | | | | | | | 0 | | | | | | | | | | | | putative BRCA1 domain protein | | | | | | | | | | | | | |  |  |  |  |  |  |
| EFV59131.1 | | | | | | | | | 0 | | | | | | | | | | | | forkhead box protein E1 | | | | | | | | | | | | | |  |  |  |  |  |  |
| EFV59133.1 | | | | | | | | | 0 | | | | | | | | | | | | WD repeat-containing protein 57 | | | | | | | | | | | | | |  |  |  |  |  |  |
| EFV59137.1 | | | | | | | | | 0 | | | | | | | | | | | | putative homeobox domain protein | | | | | | | | | | | | | |  |  |  |  |  |  |
| EFV59144.1 | | | | | | | | | 0 | | | | | | | | | | | | putative mushroom body large-type Kenyon cell-specific protein 1 | | | | | | | | | | | | | |  |  |  |  |  |  |
| EFV59164.1 | | | | | | | | | 0 | | | | | | | | | | | | kelch domain-containing protein 3 | | | | | | | | | | | | | |  |  |  |  |  |  |
| EFV59174.1 | | | | | | | | | 0 | | | | | | | | | | | | putative glucosamine 6-phosphate N-acetyltransferase | | | | | | | | | | | | | |  |  |  |  |  |  |
| EFV59175.1 | | | | | | | | | 0 | | | | | | | | | | | | ribosomal protein S12 | | | | | | | | | | | | | |  |  |  |  |  |  |
| EFV59188.1 | | | | | | | | | 0 | | | | | | | | | | | | FERM domain-containing protein 1 | | | | | | | | | | | | | |  |  |  |  |  |  |
| EFV59201.1 | | | | | | | | | 0 | | | | | | | | | | | | putative immunoglobulin domain protein | | | | | | | | | | | | | |  |  |  |  |  |  |
| EFV59205.1 | | | | | | | | | 0 | | | | | | | | | | | | putative globin | | | | | | | | | | | | | |  |  |  |  |  |  |
| EFV59212.1 | | | | | | | | | 0 | | | | | | | | | | | | coatomer subunit gamma-2 | | | | | | | | | | | | | |  |  |  |  |  |  |
| EFV59223.1 | | | | | | | | | 0 | | | | | | | | | | | | putative SacI y domain protein | | | | | | | | | | | | | |  |  |  |  |  |  |
| EFV59230.1 | | | | | | | | | 0 | | | | | | | | | | | | U4/U6.U5 tri-snRNP-associated protein 2 | | | | | | | | | | | | | |  |  |  |  |  |  |
| EFV59279.1 | | | | | | | | | 0 | | | | | | | | | | | | PRKCA-binding protein | | | | | | | | | | | | | |  |  |  |  |  |  |
| EFV59284.1 | | | | | | | | | 0 | | | | | | | | | | | | 6-phosphogluconate dehydrogenase, decarboxylating | | | | | | | | | | | | | |  |  |  |  |  |  |
| EFV59293.1 | | | | | | | | | 0 | | | | | | | | | | | | | phosphatidylinositol-3,4,5-trisphosphate 3-phosphatase PTEN | | | | | | | | | | | | | |  |  |  |  |  |
| EFV59301.1 | | | | | | | | | 0 | | | | | | | | | | | | | putative G-patch domain protein | | | | | | | | | | | | | |  |  |  |  |  |
| EFV59302.1 | | | | | | | | | 0 | | | | | | | | | | | | | mediator of RNA polymerase II transcription subunit 10 | | | | | | | | | | | | | |  |  |  |  |  |
| EFV59307.1 | | | | | | | | | 0 | | | | | | | | | | | | | putative peptidase family M1 | | | | | | | | | | | | | |  |  |  |  |  |
| EFV59329.1 | | | | | | | | | 0 | | | | | | | | | | | | | putative Ku70/Ku80 beta-barrel domain protein | | | | | | | | | | | | | |  |  |  |  |  |
| EFV59335.1 | | | | | | | | | 0 | | | | | | | | | | | | | insulinoma-associated protein 2 | | | | | | | | | | | | | |  |  |  |  |  |
| EFV59360.1 | | | | | | | | | 0 | | | | | | | | | | | | | putative chitin binding Peritrophin-A domain protein | | | | | | | | | | | | | |  |  |  |  |  |
| EFV59373.1 | | | | | | | | | 0 | | | | | | | | | | | | | conserved hypothetical protein | | | | | | | | | | | | | |  |  |  |  |  |
| EFV59378.1 | | | | | | | | | 0 | | | | | | | | | | | | | protein SAND | | | | | | | | | | | | | |  |  |  |  |  |
| EFV59382.1 | | | | | | | | | 0 | | | | | | | | | | | | | sphingomyelin synthase-related protein 1 | | | | | | | | | | | | | |  |  |  |  |  |
| EFV59383.1 | | | | | | | | | 0 | | | | | | | | | | | | | vinculin family protein | | | | | | | | | | | | | |  |  |  |  |  |
| EFV59404.1 | | | | | | | | | 0 | | | | | | | | | | | | | putative proton-coupled amino acid transporter 4 | | | | | | | | | | | | | |  |  |  |  |  |
| EFV59413.1 | | | | | | | | | 0 | | | | | | | | | | | | | conserved hypothetical protein | | | | | | | | | | | | | |  |  |  |  |  |
| EFV59416.1 | | | | | | | | | 0 | | | | | | | | | | | | | putative NSFL1 cofactor p47 | | | | | | | | | | | | | |  |  |  |  |  |
| EFV59418.1 | | | | | | | | | 0 | | | | | | | | | | | | | putative fibronectin type III domain protein | | | | | | | | | | | | | |  |  |  |  |  |
| EFV59434.1 | | | | | | | | | 0 | | | | | | | | | | | | | GTP-binding protein yptV4 | | | | | | | | | | | | | |  |  |  |  |  |
| EFV59437.1 | | | | | | | | | 0 | | | | | | | | | | | | | protein CWC15 | | | | | | | | | | | | | |  |  |  |  |  |
| EFV59446.1 | | | | | | | | | 0 | | | | | | | | | | | | | WD domain, G-beta repeat-containing domain protein | | | | | | | | | | | | | |  |  |  |  |  |
| EFV59469.1 | | | | | | | | | 0 | | | | | | | | | | | | | putative CBS domain pair | | | | | | | | | | | | | |  |  |  |  |  |
| EFV59495.1 | | | | | | | | | 0 | | | | | | | | | | | | | Sly1 protein-like protein | | | | | | | | | | | | | |  |  |  |  |  |
| EFV59501.1 | | | | | | | | | 0 | | | | | | | | | | | | | putative dipeptidyl aminopeptidase | | | | | | | | | | | | | |  |  |  |  |  |
| EFV59505.1 | | | | | | | | | 0 | | | | | | | | | | | | | putative kinase domain protein | | | | | | | | | | | | | |  |  |  |  |  |
| EFV59530.1 | | | | | | | | | 0 | | | | | | | | | | | | | cytochrome c oxidase polypeptide VIb | | | | | | | | | | | | | |  |  |  |  |  |
| EFV59547.1 | | | | | | | | | 0 | | | | | | | | | | | | | conserved hypothetical protein | | | | | | | | | | | | | |  |  |  |  |  |
| EFV59548.1 | | | | | | | | | 0 | | | | | | | | | | | | | Sel1 repeat family protein | | | | | | | | | | | | | |  |  |  |  |  |
| EFV59558.1 | | | | | | | | | 0 | | | | | | | | | | | | | putative homeobox domain protein | | | | | | | | | | | | | |  |  |  |  |  |
| EFV59570.1 | | | | | | | | | 0 | | | | | | | | | | | | | Pre-mRNA-splicing factor SLU7 | | | | | | | | | | | | | |  |  |  |  |  |
| EFV59576.1 | | | | | | | | | 0 | | | | | | | | | | | | | succinate dehydrogenase, flavoprotein subunit | | | | | | | | | | | | | |  |  |  |  |  |
| EFV59578.1 | | | | | | | | | 0 | | | | | | | | | | | | | putative transcription initiation factor TFIID subunit 4 | | | | | | | | | | | | | |  |  |  |  |  |
| EFV59622.1 | | | | | | | | | 0 | | | | | | | | | | | | | 60S ribosomal protein L13 | | | | | | | | | | | | | |  |  |  |  |  |
| EFV59661.1 | | | | | | | | | 0 | | | | | | | | | | | | | LIM domain-containing protein unc-97 | | | | | | | | | | | | | |  |  |  |  |  |
| EFV59697.1 | | | | | | | | | 0 | | | | | | | | | | | | | hypothetical protein Tsp_06863 | | | | | | | | | | | | | |  |  |  |  |  |
| EFV59700.1 | | | | | | | | | 0 | | | | | | | | | | | | | putative Ras association domain protein | | | | | | | | | | | | | |  |  |  |  |  |
| EFV59708.1 | | | | | | | | | 0 | | | | | | | | | | | | | CDGSH iron sulfur domain-containing protein 2-A | | | | | | | | | | | | | |  |  |  |  |  |
| EFV59723.1 | | | | | | | | | 0 | | | | | | | | | | | | | uridine/cytidine kinase A | | | | | | | | | | | | | |  |  |  |  |  |
| EFV59726.1 | | | | | | | | | 0 | | | | | | | | | | | | | conserved hypothetical protein | | | | | | | | | | | | | |  |  |  |  |  |
| EFV59727.1 | | | | | | | | | 0 | | | | | | | | | | | | | conserved hypothetical protein | | | | | | | | | | | | | |  |  |  |  |  |
| EFV59764.1 | | | | | | | | | 0 | | | | | | | | | | | | | brain protein 44 | | | | | | | | | | | | | |  |  |  |  |  |
| EFV59786.1 | | | | | | | | | 0 | | | | | | | | | | | | | putative RhoGEF domain protein | | | | | | | | | | | | | |  |  |  |  |  |
| EFV59796.1 | | | | | | | | | | 0 | | | | | | | | | | | exosome complex exonuclease RRP4 | | | | | | | | | | | | | | |  |  |  |  |  |
| EFV59823.1 | | | | | | | | | | 0 | | | | | | | | | | | ATP-binding domain-containing protein 3 | | | | | | | | | | | | | | |  |  |  |  |  |
| EFV59851.1 | | | | | | | | | | 0 | | | | | | | | | | | protein pellino | | | | | | | | | | | | | | |  |  |  |  |  |
| EFV59896.1 | | | | | | | | | | 0 | | | | | | | | | | | 60S ribosomal protein L35 | | | | | | | | | | | | | | |  |  |  |  |  |
| EFV59963.1 | | | | | | | | | | 0 | | | | | | | | | | | ST7 protein | | | | | | | | | | | | | | |  |  |  |  |  |
| EFV59968.1 | | | | | | | | | | 0 | | | | | | | | | | | elongation of very long chain fatty acids protein 6 | | | | | | | | | | | | | | |  |  |  |  |  |
| EFV60018.1 | | | | | | | | | | 0 | | | | | | | | | | | putative phorbol esters/diacylglycerol binding domain protein | | | | | | | | | | | | | | |  |  |  |  |  |
| EFV60024.1 | | | | | | | | | | 0 | | | | | | | | | | | translation initiation factor eIF-2B subunit beta | | | | | | | | | | | | | | |  |  |  |  |  |
| EFV60038.1 | | | | | | | | | | 0 | | | | | | | | | | | ferredoxin--NADP reductase | | | | | | | | | | | | | | |  |  |  |  |  |
| EFV60047.1 | | | | | | | | | | 0 | | | | | | | | | | | conserved hypothetical protein | | | | | | | | | | | | | | |  |  |  |  |  |
| EFV60050.1 | | | | | | | | | | 0 | | | | | | | | | | | adapter molecule Crk | | | | | | | | | | | | | | |  |  |  |  |  |
| EFV60080.1 | | | | | | | | | | 0 | | | | | | | | | | | putative ribosomal protein S18 | | | | | | | | | | | | | | |  |  |  |  |  |
| EFV60084.1 | | | | | | | | | | 0 | | | | | | | | | | | lysosomal aspartic protease | | | | | | | | | | | | | | |  |  |  |  |  |
| EFV60121.1 | | | | | | | | | | 0 | | | | | | | | | | | surface antigen repeat family protein | | | | | | | | | | | | | | |  |  |  |  |  |
| EFV60129.1 | | | | | | | | | | 0 | | | | | | | | | | | hypothetical protein Tsp_05964 | | | | | | | | | | | | | | |  |  |  |  |  |
| EFV60134.1 | | | | | | | | | | 0 | | | | | | | | | | | pyruvate carboxylase 1 | | | | | | | | | | | | | | |  |  |  |  |  |
| EFV60140.1 | | | | | | | | | | 0 | | | | | | | | | | | DnaJ protein subfamily B member 12 | | | | | | | | | | | | | | |  |  |  |  |  |
| EFV60141.1 | | | | | | | | | | 0 | | | | | | | | | | | solute carrier family 41 member 1 | | | | | | | | | | | | | | |  |  |  |  |  |
| EFV60143.1 | | | | | | | | | | 0 | | | | | | | | | | | RNA polymerase-associated protein LEO1 | | | | | | | | | | | | | | |  |  |  |  |  |
| EFV60157.1 | | | | | | | | | | 0 | | | | | | | | | | | nicotinamide phosphoribosyltransferase | | | | | | | | | | | | | | |  |  |  |  |  |
| EFV60165.1 | | | | | | | | | | 0 | | | | | | | | | | | alpha-mannosidase 2C1 | | | | | | | | | | | | | | |  |  |  |  |  |
| EFV60170.1 | | | | | | | | | | 0 | | | | | | | | | | | RING finger protein 13 | | | | | | | | | | | | | | |  |  |  |  |  |
| EFV60181.1 | | | | | | | | | | 0 | | | | | | | | | | | conserved hypothetical protein | | | | | | | | | | | | | | |  |  |  |  |  |
| EFV60187.1 | | | | | | | | | | 0 | | | | | | | | | | | putative thromboxane-A synthase | | | | | | | | | | | | | | |  |  |  |  |  |
| EFV60188.1 | | | | | | | | | | 0 | | | | | | | | | | | 5'-AMP-activated protein kinase subunit beta-1 | | | | | | | | | | | | | | |  |  |  |  |  |
| EFV60199.1 | | | | | | | | | | 0 | | | | | | | | | | | translationally-controlled tumor protein-like protein | | | | | | | | | | | | | | |  |  |  |  |  |
| EFV60201.1 | | | | | | | | | | 0 | | | | | | | | | | | vacuolar protein sorting-associated protein 41-like protein | | | | | | | | | | | | | | |  |  |  |  |  |
| EFV60217.1 | | | | | | | | | | 0 | | | | | | | | | | | JmjC domain-containing protein 5 | | | | | | | | | | | | | | |  |  |  |  |  |
| EFV60220.1 | | | | | | | | | | 0 | | | | | | | | | | | general transcription factor IIH subunit 4 | | | | | | | | | | | | | | |  |  |  |  |  |
| EFV60234.1 | | | | | | | | | | 0 | | | | | | | | | | | putative calcium-translocating P-type ATPase, PMCA-type | | | | | | | | | | | | | | |  |  |  |  |  |
| EFV60271.1 | | | | | | | | | | 0 | | | | | | | | | | | glycogenin-1 | | | | | | | | | | | | | | |  |  |  |  |  |
| EFV60294.1 | | | | | | | | | | 0 | | | | | | | | | | | protein OPI10-like protein | | | | | | | | | | | | | | |  |  |  |  |  |
| EFV60316.1 | | | | | | | | | | 0 | | | | | | | | | | | putative MIZ/SP-RING zinc finger, partial | | | | | | | | | | | | | | |  |  |  |  |  |
| EFV60320.1 | | | | | | | | | | 0 | | | | | | | | | | | protein transport protein SEC24 | | | | | | | | | | | | | | |  |  |  |  |  |
| EFV60336.1 | | | | | | | | | | 0 | | | | | | | | | | | protein SET | | | | | | | | | | | | | | |  |  |  |  |  |
| EFV60363.1 | | | | | | | | | | 0 | | | | | | | | | | | mediator of RNA polymerase II transcription subunit 4 | | | | | | | | | | | | | | |  |  |  |  |  |
| EFV60367.1 | | | | | | | | | | 0 | | | | | | | | | | | U1 small nuclear ribonucleoprotein C | | | | | | | | | | | | | | |  |  |  |  |  |
| EFV60379.1 | | | | | | | | | | 0 | | | | | | | | | | | HAD-superfamily hydrolase, subfamily IA, variant 3 | | | | | | | | | | | | | | |  |  |  |  |  |
| EFV60381.1 | | | | | | | | | | 0 | | | | | | | | | | | | | zinc phosphodiesterase ELAC protein 2 | | | | | | | | | | | | | | |  |  |  |
| EFV60393.1 | | | | | | | | | | 0 | | | | | | | | | | | | | conserved hypothetical protein | | | | | | | | | | | | | | |  |  |  |
| EFV60396.1 | | | | | | | | | | 0 | | | | | | | | | | | | | Pre-mRNA cleavage complex II protein Clp1 | | | | | | | | | | | | | | |  |  |  |
| EFV60416.1 | | | | | | | | | | 0 | | | | | | | | | | | | | erythroid transcription factor | | | | | | | | | | | | | | |  |  |  |
| EFV60445.1 | | | | | | | | | | 0 | | | | | | | | | | | | | dystroglycan | | | | | | | | | | | | | | |  |  |  |
| EFV60448.1 | | | | | | | | | | 0 | | | | | | | | | | | | | cytochrome c | | | | | | | | | | | | | | |  |  |  |
| EFV60478.1 | | | | | | | | | | 0 | | | | | | | | | | | | | cystinosin | | | | | | | | | | | | | | |  |  |  |
| EFV60512.1 | | | | | | | | | | 0 | | | | | | | | | | | | | calcium-binding protein p22 | | | | | | | | | | | | | | |  |  |  |
| EFV60513.1 | | | | | | | | | | 0 | | | | | | | | | | | | | poly(A) polymerase gamma | | | | | | | | | | | | | | |  |  |  |
| EFV60533.1 | | | | | | | | | | 0 | | | | | | | | | | | | | cuticle collagen 34 protein | | | | | | | | | | | | | | |  |  |  |
| EFV60567.1 | | | | | | | | | | 0 | | | | | | | | | | | | | cAMP-dependent protein kinase catalytic subunit beta | | | | | | | | | | | | | | |  |  |  |
| EFV60580.1 | | | | | | | | | | 0 | | | | | | | | | | | | | conserved hypothetical protein | | | | | | | | | | | | | | |  |  |  |
| EFV60589.1 | | | | | | | | | | 0 | | | | | | | | | | | | | interferon regulatory factor 2-binding protein 2-B | | | | | | | | | | | | | | |  |  |  |
| EFV60595.1 | | | | | | | | | | 0 | | | | | | | | | | | | | AP-2 complex subunit sigma | | | | | | | | | | | | | | |  |  |  |
| EFV60602.1 | | | | | | | | | | 0 | | | | | | | | | | | | | cuticle collagen 39 | | | | | | | | | | | | | | |  |  |  |
| EFV60604.1 | | | | | | | | | | 0 | | | | | | | | | | | | | conserved hypothetical protein | | | | | | | | | | | | | | |  |  |  |
| EFV60606.1 | | | | | | | | | | 0 | | | | | | | | | | | | | conserved hypothetical protein | | | | | | | | | | | | | | |  |  |  |
| EFV60623.1 | | | | | | | | | | 0 | | | | | | | | | | | | | neuroendocrine protein 7B2 | | | | | | | | | | | | | | |  |  |  |
| EFV60629.1 | | | | | | | | | | 0 | | | | | | | | | | | | | 60S ribosomal protein L5-A | | | | | | | | | | | | | | |  |  |  |
| EFV60641.1 | | | | | | | | | | 0 | | | | | | | | | | | | | peptidase family M3 | | | | | | | | | | | | | | |  |  |  |
| EFV60646.1 | | | | | | | | | | 0 | | | | | | | | | | | | | 60S ribosomal protein L30 | | | | | | | | | | | | | | |  |  |  |
| EFV60649.1 | | | | | | | | | | 0 | | | | | | | | | | | | | conserved hypothetical protein | | | | | | | | | | | | | | |  |  |  |
| EFV60653.1 | | | | | | | | | | 0 | | | | | | | | | | | | | conserved hypothetical protein | | | | | | | | | | | | | | |  |  |  |
| EFV60654.1 | | | | | | | | | | 0 | | | | | | | | | | | | | LBP / BPI / CETP family domain protein | | | | | | | | | | | | | | |  |  |  |
| EFV60666.1 | | | | | | | | | | 0 | | | | | | | | | | | | | putative tetratricopeptide repeat-containing domain protein | | | | | | | | | | | | | | |  |  |  |
| EFV60704.1 | | | | | | | | | | 0 | | | | | | | | | | | | | prolyl 4-hydroxylase subunit alpha-2 | | | | | | | | | | | | | | |  |  |  |
| EFV60718.1 | | | | | | | | | | 0 | | | | | | | | | | | | | hypothetical protein Tsp_03303 | | | | | | | | | | | | | | |  |  |  |
| EFV60724.1 | | | | | | | | | | 0 | | | | | | | | | | | | | zinc finger protein | | | | | | | | | | | | | | |  |  |  |
| EFV60743.1 | | | | | | | | | | 0 | | | | | | | | | | | | | adenosine deaminase | | | | | | | | | | | | | | |  |  |  |
| EFV60744.1 | | | | | | | | | | 0 | | | | | | | | | | | | | exocyst complex component 6 | | | | | | | | | | | | | | |  |  |  |
| EFV60745.1 | | | | | | | | | | 0 | | | | | | | | | | | | | YY1-associated factor 2 | | | | | | | | | | | | | | |  |  |  |
| EFV60751.1 | | | | | | | | | | 0 | | | | | | | | | | | | | putative KH domain protein | | | | | | | | | | | | | | |  |  |  |
| EFV60786.1 | | | | | | | | | | 0 | | | | | | | | | | | | | testican-1 | | | | | | | | | | | | | | |  |  |  |
| EFV60812.1 | | | | | | | | | | 0 | | | | | | | | | | | | | BAT2 domain protein | | | | | | | | | | | | | | |  |  |  |
| EFV60822.1 | | | | | | | | | | 0 | | | | | | | | | | | | | conserved domain protein | | | | | | | | | | | | | | |  |  |  |
| EFV60852.1 | | | | | | | | | | 0 | | | | | | | | | | | | | conserved hypothetical protein, partial | | | | | | | | | | | | | | |  |  |  |
| EFV60873.1 | | | | | | | | | | 0 | | | | | | | | | | | | | putative protein RIC1-like protein | | | | | | | | | | | | | | |  |  |  |
| EFV60907.1 | | | | | | | | | | 0 | | | | | | | | | | | | | partitioning defective protein 6 | | | | | | | | | | | | | | |  |  |  |
| EFV60919.1 | | | | | | | | | | | 0 | | | | | | | | | | | | putative nudix hydrolase 6 | | | | | | | | | | | | | |  |  |  |  |
| EFV60920.1 | | | | | | | | | | | 0 | | | | | | | | | | | | putative leucine Rich repeat-containing domain protein | | | | | | | | | | | | | |  |  |  |  |
| EFV60948.1 | | | | | | | | | | | 0 | | | | | | | | | | | | conserved hypothetical protein, partial | | | | | | | | | | | | | |  |  |  |  |
| EFV60961.1 | | | | | | | | | | | 0 | | | | | | | | | | | | FYVE, RhoGEF and PH domain-containing protein 3 | | | | | | | | | | | | | |  |  |  |  |
| EFV60966.1 | | | | | | | | | | | 0 | | | | | | | | | | | | neuroendocrine convertase 2 | | | | | | | | | | | | | |  |  |  |  |
| EFV60967.1 | | | | | | | | | | | 0 | | | | | | | | | | | | hypothetical protein Tsp_03059 | | | | | | | | | | | | | |  |  |  |  |
| EFV60969.1 | | | | | | | | | | | 0 | | | | | | | | | | | | MAGUK p55 subfamily member 5 | | | | | | | | | | | | | |  |  |  |  |
| EFV60977.1 | | | | | | | | | | | 0 | | | | | | | | | | | | selenide, water dikinase | | | | | | | | | | | | | |  |  |  |  |
| EFV60978.1 | | | | | | | | | | | 0 | | | | | | | | | | | | putative WAP-type 'four-disulfide core' | | | | | | | | | | | | | |  |  |  |  |
| EFV60982.1 | | | | | | | | | | | 0 | | | | | | | | | | | | 60S ribosomal protein L34 | | | | | | | | | | | | | |  |  |  |  |
| EFV60985.1 | | | | | | | | | | | 0 | | | | | | | | | | | | protein ADRM1 | | | | | | | | | | | | | |  |  |  |  |
| EFV60986.1 | | | | | | | | | | | 0 | | | | | | | | | | | | cyclin-C | | | | | | | | | | | | | |  |  |  |  |
| EFV60987.1 | | | | | | | | | | | 0 | | | | | | | | | | | | actin-related protein 2 | | | | | | | | | | | | | |  |  |  |  |
| EFV60993.1 | | | | | | | | | | | 0 | | | | | | | | | | | | gephyrin | | | | | | | | | | | | | |  |  |  |  |
| EFV61046.1 | | | | | | | | | | | 0 | | | | | | | | | | | | E3 ubiquitin-protein ligase synoviolin-A | | | | | | | | | | | | | |  |  |  |  |
| EFV61050.1 | | | | | | | | | | | 0 | | | | | | | | | | | | peptidase, S1A subfamily | | | | | | | | | | | | | |  |  |  |  |
| EFV61063.1 | | | | | | | | | | | 0 | | | | | | | | | | | | RAS guanyl-releasing protein 1 | | | | | | | | | | | | | |  |  |  |  |
| EFV61123.1 | | | | | | | | | | | 0 | | | | | | | | | | | | putative kunitz/Bovine pancreatic trypsin inhibitor domain protein, partial | | | | | | | | | | | | | |  |  |  |  |
| EFV61143.1 | | | | | | | | | | | 0 | | | | | | | | | | | | ankyrin repeat-containing domain protein | | | | | | | | | | | | | |  |  |  |  |
| EFV61170.1 | | | | | | | | | | | 0 | | | | | | | | | | | | putative calcium binding EGF domain protein | | | | | | | | | | | | | |  |  |  |  |
| EFV61183.1 | | | | | | | | | | | 0 | | | | | | | | | | | | putative zinc finger protein | | | | | | | | | | | | | |  |  |  |  |
| EFV61190.1 | | | | | | | | | | | 0 | | | | | | | | | | | | putative degenerin mec-4 | | | | | | | | | | | | | |  |  |  |  |
| EFV61199.1 | | | | | | | | | | | 0 | | | | | | | | | | | | EH domain-containing protein | | | | | | | | | | | | | |  |  |  |  |
| EFV61204.1 | | | | | | | | | | | 0 | | | | | | | | | | | | SMEK protein | | | | | | | | | | | | | |  |  |  |  |
| EFV61230.1 | | | | | | | | | | | 0 | | | | | | | | | | | | protein YIPF3 | | | | | | | | | | | | | |  |  |  |  |
| EFV61233.1 | | | | | | | | | | | 0 | | | | | | | | | | | | Rab GDP dissociation inhibitor alpha | | | | | | | | | | | | | |  |  |  |  |
| EFV61244.1 | | | | | | | | | | | 0 | | | | | | | | | | | | autophagy protein Apg9 | | | | | | | | | | | | | |  |  |  |  |
| EFV61250.1 | | | | | | | | | | | 0 | | | | | | | | | | | | seryl-tRNA synthetase, cytoplasmic | | | | | | | | | | | | | |  |  |  |  |
| EFV61255.1 | | | | | | | | | | | 0 | | | | | | | | | | | | metal cation transporter, zinc (Zn2+)-iron (Fe2+) permease (ZIP) family | | | | | | | | | | | | | |  |  |  |  |
| EFV61375.1 | | | | | | | | | | | 0 | | | | | | | | | | | | RNA-binding protein 47 | | | | | | | | | | | | | |  |  |  |  |
| EFV61397.1 | | | | | | | | | | | 0 | | | | | | | | | | | | GTPase KRas | | | | | | | | | | | | | |  |  |  |  |
| EFV61402.1 | | | | | | | | | | | 0 | | | | | | | | | | | | nuclear GTP-binding protein NUG1, partial | | | | | | | | | | | | | |  |  |  |  |
| EFV61417.1 | | | | | | | | | | | 0 | | | | | | | | | | | | putative WD repeat-containing protein 85 | | | | | | | | | | | | | |  |  |  |  |
| EFV61421.1 | | | | | | | | | | | 0 | | | | | | | | | | | | surfeit locus protein 4 | | | | | | | | | | | | | |  |  |  |  |
| EFV61442.1 | | | | | | | | | | | 0 | | | | | | | | | | | | transmembrane and TPR repeat-containing protein 4 | | | | | | | | | | | | | |  |  |  |  |
| EFV61445.1 | | | | | | | | | | | 0 | | | | | | | | | | | | tRNA (guanine-N(7)-)-methyltransferase | | | | | | | | | | | | | |  |  |  |  |
| EFV61446.1 | | | | | | | | | | | | 0 | | | | | | | | | | putative disintegrin | | | | | | | | | | | | | | |  |  |  |  |
| EFV61454.1 | | | | | | | | | | | | 0 | | | | | | | | | | mRNA-capping enzyme | | | | | | | | | | | | | | |  |  |  |  |
| EFV61456.1 | | | | | | | | | | | | 0 | | | | | | | | | | putative leucine Rich repeat-containing domain protein | | | | | | | | | | | | | | |  |  |  |  |
| EFV61472.1 | | | | | | | | | | | | 0 | | | | | | | | | | neurogenin-3 | | | | | | | | | | | | | | |  |  |  |  |
| EFV61541.1 | | | | | | | | | | | | 0 | | | | | | | | | | activator ofheat shock protein ATPase protein | | | | | | | | | | | | | | |  |  |  |  |
| EFV61552.1 | | | | | | | | | | | | 0 | | | | | | | | | | putative ubiquinol-cytochrome c reductase, iron-sulfur subunit | | | | | | | | | | | | | | |  |  |  |  |
| EFV61555.1 | | | | | | | | | | | | 0 | | | | | | | | | | conserved hypothetical protein | | | | | | | | | | | | | | |  |  |  |  |
| EFV61579.1 | | | | | | | | | | | | 0 | | | | | | | | | | protein FAM50A | | | | | | | | | | | | | | |  |  |  |  |
| EFV61580.1 | | | | | | | | | | | | 0 | | | | | | | | | | vacuolar ATP synthase subunit e 2 | | | | | | | | | | | | | | |  |  |  |  |
| EFV61581.1 | | | | | | | | | | | | 0 | | | | | | | | | | conserved hypothetical protein | | | | | | | | | | | | | | |  |  |  |  |
| EFV61591.1 | | | | | | | | | | | | 0 | | | | | | | | | | ryR and IP3R associated family protein, partial | | | | | | | | | | | | | | |  |  |  |  |
| EFV61632.1 | | | | | | | | | | | | 0 | | | | | | | | | | tyrosyl-tRNA synthetase, cytoplasmic | | | | | | | | | | | | | | |  |  |  |  |
| EFV61654.1 | | | | | | | | | | | | 0 | | | | | | | | | | stromal interaction molecule-like protein | | | | | | | | | | | | | | |  |  |  |  |
| EFV61657.1 | | | | | | | | | | | | 0 | | | | | | | | | | conserved hypothetical protein | | | | | | | | | | | | | | |  |  |  |  |
| EFV61660.1 | | | | | | | | | | | | 0 | | | | | | | | | | KRR1 small subunit processome component-like protein | | | | | | | | | | | | | | |  |  |  |  |
| EFV61691.1 | | | | | | | | | | | | 0 | | | | | | | | | | protein RER1 | | | | | | | | | | | | | | |  |  |  |  |
| EFV61716.1 | | | | | | | | | | | | 0 | | | | | | | | | | zinc finger protein | | | | | | | | | | | | | | |  |  |  |  |
| EFV61745.1 | | | | | | | | | | | | 0 | | | | | | | | | | N-acetyltransferase ESCO1 | | | | | | | | | | | | | | |  |  |  |  |
| EFV61832.1 | | | | | | | | | | | | 0 | | | | | | | | | | PDF receptor | | | | | | | | | | | | | | |  |  |  |  |
| EFV61876.1 | | | | | | | | | | | | 0 | | | | | | | | | | eukaryotic peptide chain release factor GTP-binding subunit ERF3A | | | | | | | | | | | | | | |  |  |  |  |
| EFV61879.1 | | | | | | | | | | | | 0 | | | | | | | | | | ribosome biogenesis protein NSA2-like protein, partial | | | | | | | | | | | | | | |  |  |  |  |
| EFV61909.1 | | | | | | | | | | | | 0 | | | | | | | | | | signal peptide peptidase family protein, partial | | | | | | | | | | | | | | |  |  |  |  |
| EFV61969.1 | | | | | | | | | | | | 0 | | | | | | | | | | putative PH domain protein | | | | | | | | | | | | | | |  |  |  |  |
| EFV61978.1 | | | | | | | | | | | | 0 | | | | | | | | | | hint module superfamily | | | | | | | | | | | | | | |  |  |  |  |
| EFV61981.1 | | | | | | | | | | | | 0 | | | | | | | | | | putative helix-loop-helix DNA-binding domain protein | | | | | | | | | | | | | | |  |  |  |  |
| EFV61997.1 | | | | | | | | | | | | 0 | | | | | | | | | | coatomer subunit delta | | | | | | | | | | | | | | |  |  |  |  |
| EFV61998.1 | | | | | | | | | | | | 0 | | | | | | | | | | putative phosphoglycolate/pyridoxal phosphate phosphatase family protein | | | | | | | | | | | | | | |  |  |  |  |
| EFV62007.1 | | | | | | | | | | | | 0 | | | | | | | | | | putative BSD domain protein | | | | | | | | | | | | | | |  |  |  |  |
| EFV62013.1 | | | | | | | | | | | | 0 | | | | | | | | | | conserved hypothetical protein | | | | | | | | | | | | | | |  |  |  |  |
| EFV62060.1 | | | | | | | | | | | | 0 | | | | | | | | | | putative leucine Rich repeat-containing domain protein | | | | | | | | | | | | | | |  |  |  |  |
| EFV62081.1 | | | | | | | | | | | | 0 | | | | | | | | | | putative BUD13-like protein | | | | | | | | | | | | | | |  |  |  |  |
| EFV62093.1 | | | | | | | | | | | | 0 | | | | | | | | | | ectonucleotide pyrophosphatase/phosphodiesterase family member 5 precursor | | | | | | | | | | | | | | |  |  |  |  |
| EFV62145.1 | | | | | | | | | | | | 0 | | | | | | | | | | golgi SNAP receptor complex member 1 | | | | | | | | | | | | | | |  |  |  |  |
| EFV62150.1 | | | | | | | | | | | | 0 | | | | | | | | | | putative SPRY domain protein | | | | | | | | | | | | | | |  |  |  |  |
| EFV62160.1 | | | | | | | | | | | | 0 | | | | | | | | | | protein spinster protein | | | | | | | | | | | | | | |  |  |  |  |
| EFV62174.1 | | | | | | | | | | | | 0 | | | | | | | | | | conserved hypothetical protein | | | | | | | | | | | | | | |  |  |  |  |
| EFV62202.1 | | | | | | | | | | | | | 0 | | | | | | | | | | | sterile alpha and TIR motif protein-containing protein tir-1 | | | | | | | | | | | | | | | |  |
| EFV62220.1 | | | | | | | | | | | | | 0 | | | | | | | | | | | FACT complex subunit Ssrp1 | | | | | | | | | | | | | | | |  |
| EFV62236.1 | | | | | | | | | | | | | 0 | | | | | | | | | | | putative deoxycytidylate deaminase, partial | | | | | | | | | | | | | | | |  |
| EFV62240.1 | | | | | | | | | | | | | 0 | | | | | | | | | | | putative C2 domain protein | | | | | | | | | | | | | | | |  |
| EFV62244.1 | | | | | | | | | | | | | 0 | | | | | | | | | | | conserved hypothetical protein | | | | | | | | | | | | | | | |  |
| EFV62248.1 | | | | | | | | | | | | | 0 | | | | | | | | | | | DNA excision repair protein haywire | | | | | | | | | | | | | | | |  |
| EFV62271.1 | | | | | | | | | | | | | 0 | | | | | | | | | | | breast carcinoma-amplified sequence 3-like protein | | | | | | | | | | | | | | | |  |
| EFV62291.1 | | | | | | | | | | | | | 0 | | | | | | | | | | | y+L amino acid transporter 1 | | | | | | | | | | | | | | | |  |
| EFV62300.1 | | | | | | | | | | | | | 0 | | | | | | | | | | | putative transcription factor TFIIB repeat-containing domain protein | | | | | | | | | | | | | | | |  |
| EFV62314.1 | | | | | | | | | | | | | 0 | | | | | | | | | | | Pre-mRNA-splicing factor 38A | | | | | | | | | | | | | | | |  |
| EFV62321.1 | | | | | | | | | | | | | 0 | | | | | | | | | | | ubiquitin family protein, partial | | | | | | | | | | | | | | | |  |
| EFV62339.1 | | | | | | | | | | | | | 0 | | | | | | | | | | | conserved hypothetical protein | | | | | | | | | | | | | | | |  |
| EFV62362.1 | | | | | | | | | | | | | 0 | | | | | | | | | | | synaptic glycoprotein SC2 | | | | | | | | | | | | | | | |  |
| EFV62406.1 | | | | | | | | | | | | | 0 | | | | | | | | | | | zinc finger protein | | | | | | | | | | | | | | | |  |
| EFV62441.1 | | | | | | | | | | | | | 0 | | | | | | | | | | | phosphoglucomutase-2 | | | | | | | | | | | | | | | |  |
| EFV62453.1 | | | | | | | | | | | | | 0 | | | | | | | | | | | conserved domain protein | | | | | | | | | | | | | | | |  |
| EFV62456.1 | | | | | | | | | | | | | 0 | | | | | | | | | | | homeobox protein abdominal-B | | | | | | | | | | | | | | | |  |
| EFV62515.1 | | | | | | | | | | | | | 0 | | | | | | | | | | | conserved hypothetical protein | | | | | | | | | | | | | | | |  |
| EFV62526.1 | | | | | | | | | | | | | 0 | | | | | | | | | | | conserved hypothetical protein | | | | | | | | | | | | | | | |  |
| EFV62537.1 | | | | | | | | | | | | | 0 | | | | | | | | | | | protein UPS1 | | | | | | | | | | | | | | | |  |
| EFV62552.1 | | | | | | | | | | | | | 0 | | | | | | | | | | | putative RNA recognition motif protein | | | | | | | | | | | | | | | |  |
| EFV47705.1 | | | | | | | | | | | | | 0.001 | | | | | | | | | | | protein couch potato, partial | | | | | | | | | | | | | | | |  |
| EFV48078.1 | | | | | | | | | | | | | 0.001 | | | | | | | | | | | conserved hypothetical protein | | | | | | | | | | | | | | | |  |
| EFV49029.1 | | | | | | | | | | | | | 0.001 | | | | | | | | | | | conserved hypothetical protein | | | | | | | | | | | | | | | |  |
| EFV50046.1 | | | | | | | | | | | | | 0.001 | | | | | | | | | | | double-strand break repair protein MRE11A | | | | | | | | | | | | | | | |  |
| EFV50703.1 | | | | | | | | | | | | | 0.001 | | | | | | | | | | | putative irregular chiasm C-roughest protein | | | | | | | | | | | | | | | |  |
| EFV50728.1 | | | | | | | | | | | | | 0.001 | | | | | | | | | | | hypothetical protein Tsp_05077 | | | | | | | | | | | | | | | |  |
| EFV51228.1 | | | | | | | | | | | | | 0.001 | | | | | | | | | | | multicopy suppressor of Ras1 | | | | | | | | | | | | | | | |  |
| EFV51367.1 | | | | | | | | | | | | | 0.001 | | | | | | | | | | | protein IMPACT | | | | | | | | | | | | | | | |  |
| EFV51596.1 | | | | | | | | | | | | | 0.001 | | | | | | | | | | | putative alanine--tRNA ligase | | | | | | | | | | | | | | | |  |
| EFV51698.1 | | | | | | | | | | | | | 0.001 | | | | | | | | | | | conserved hypothetical protein | | | | | | | | | | | | | | | |  |
| EFV52004.1 | | | | | | | | | | | | | 0.001 | | | | | | | | | | | D-glucuronyl C5-epimerase | | | | | | | | | | | | | | | |  |
| EFV52090.1 | | | | | | | | | | | | | 0.001 | | | | | | | | | | | PHR domain protein | | | | | | | | | | | | | | | |  |
| EFV52193.1 | | | | | | | | | | | | | 0.001 | | | | | | | | | | | putative O-sialoglycoprotein endopeptidase | | | | | | | | | | | | | | | |  |
| EFV52589.1 | | | | | | | | | | | | | 0.001 | | | | | | | | | | | hypothetical protein Tsp_11659 | | | | | | | | | | | | | | | |  |
| EFV52597.1 | | | | | | | | | | | | | 0.001 | | | | | | | | | | | putative DB module | | | | | | | | | | | | | | | |  |
| EFV52829.1 | | | | | | | | | | | | | 0.001 | | | | | | | | | | | phosphatidylinositol-4-phosphate 5-Kinase superfamily | | | | | | | | | | | | | | | |  |
| EFV53046.1 | | | | | | | | | | | | | 0.001 | | | | | | | | | | | putative neurotransmitter-gated ion-channel ligand binding domain protein | | | | | | | | | | | | | | | |  |
| EFV54817.1 | | | | | | | | | | | | | 0.001 | | | | | | | | | | | putative BAR domain protein | | | | | | | | | | | | | | | |  |
| EFV55223.1 | | | | | | | | | | | | | | 0.001 | | | | | | | | | | | calcium/calmodulin-dependent 3',5'-cyclic nucleotide phosphodiesterase1C | | | | | | | | | | | | | |  |  |
| EFV55486.1 | | | | | | | | | | | | | | 0.001 | | | | | | | | | | | DNA ligase 1 | | | | | | | | | | | | | |  |  |
| EFV55622.1 | | | | | | | | | | | | | | 0.001 | | | | | | | | | | | choline-phosphate cytidylyltransferase B | | | | | | | | | | | | | |  |  |
| EFV56341.1 | | | | | | | | | | | | | | 0.001 | | | | | | | | | | | signal recognition particle receptor subunit alpha | | | | | | | | | | | | | |  |  |
| EFV57406.1 | | | | | | | | | | | | | | 0.001 | | | | | | | | | | | conserved hypothetical protein | | | | | | | | | | | | | |  |  |
| EFV57431.1 | | | | | | | | | | | | | | 0.001 | | | | | | | | | | | eukaryotic porin family protein | | | | | | | | | | | | | |  |  |
| EFV57748.1 | | | | | | | | | | | | | | 0.001 | | | | | | | | | | | putative S1 RNA-binding domain-containing protein 1 | | | | | | | | | | | | | |  |  |
| EFV57827.1 | | | | | | | | | | | | | | 0.001 | | | | | | | | | | | conserved hypothetical protein | | | | | | | | | | | | | |  |  |
| EFV58355.1 | | | | | | | | | | | | | | 0.001 | | | | | | | | | | | 4-hydroxybenzoate octaprenyltransferase | | | | | | | | | | | | | |  |  |
| EFV58416.1 | | | | | | | | | | | | | | 0.001 | | | | | | | | | | | rRNA-processing protein FCF1 | | | | | | | | | | | | | |  |  |
| EFV58492.1 | | | | | | | | | | | | | | 0.001 | | | | | | | | | | | cystathionine beta-synthase | | | | | | | | | | | | | |  |  |
| EFV58553.1 | | | | | | | | | | | | | | 0.001 | | | | | | | | | | | splicing factor 3A subunit 3 | | | | | | | | | | | | | |  |  |
| EFV58915.1 | | | | | | | | | | | | | | 0.001 | | | | | | | | | | | putative actin-binding protein anillin | | | | | | | | | | | | | |  |  |
| EFV59686.1 | | | | | | | | | | | | | | 0.001 | | | | | | | | | | | orphan steroid hormone receptor 2 | | | | | | | | | | | | | |  |  |
| EFV59898.1 | | | | | | | | | | | | | | 0.001 | | | | | | | | | | | carboxylesterase family protein | | | | | | | | | | | | | |  |  |
| EFV59922.1 | | | | | | | | | | | | | | 0.001 | | | | | | | | | | | protein disulfide-isomerase 2 | | | | | | | | | | | | | |  |  |
| EFV60190.1 | | | | | | | | | | | | | | 0.001 | | | | | | | | | | | putative translation initiation factor eIF-2B subunit epsilon | | | | | | | | | | | | | |  |  |
| EFV60540.1 | | | | | | | | | | | | | | 0.001 | | | | | | | | | | | coiled-coil domain-containing protein 51 | | | | | | | | | | | | | |  |  |
| EFV60600.1 | | | | | | | | | | | | | | 0.001 | | | | | | | | | | | putative RUN and FYVE domain-containing protein 2 | | | | | | | | | | | | | |  |  |
| EFV60808.1 | | | | | | | | | | | | | | 0.001 | | | | | | | | | | | Egl nine protein | | | | | | | | | | | | | |  |  |
| EFV61162.1 | | | | | | | | | | | | | | 0.001 | | | | | | | | | | | E3 ubiquitin-protein ligase MARCH8 | | | | | | | | | | | | | |  |  |
| EFV50718.1 | | | | | | | | | | | | | | 0.002 | | | | | | | | | | | putative HEAT repeat-containing domain protein | | | | | | | | | | | | | |  |  |
| EFV50939.1 | | | | | | | | | | | | | | 0.002 | | | | | | | | | | | indoleamine 2,3-dioxygenase | | | | | | | | | | | | | |  |  |
| EFV51173.1 | | | | | | | | | | | | | | 0.002 | | | | | | | | | | | transmembrane protein 184B | | | | | | | | | | | | | |  |  |
| EFV51366.1 | | | | | | | | | | | | | | 0.002 | | | | | | | | | | | conserved hypothetical protein | | | | | | | | | | | | | |  |  |
| EFV53348.1 | | | | | | | | | | | | | | 0.002 | | | | | | | | | | | putative proto-onCogene tyrosine-protein kinase FER | | | | | | | | | | | | | |  |  |
| EFV53880.1 | | | | | | | | | | | | | | 0.002 | | | | | | | | | | | conserved hypothetical protein | | | | | | | | | | | | | |  |  |
| EFV54297.1 | | | | | | | | | | | | | | 0.002 | | | | | | | | | | | conserved hypothetical protein | | | | | | | | | | | | | |  |  |
| EFV54390.1 | | | | | | | | | | | | | | 0.002 | | | | | | | | | | | eukaryotic-type DNA primase, large subunit superfamily | | | | | | | | | | | | | |  |  |
| EFV54808.1 | | | | | | | | | | | | | | 0.002 | | | | | | | | | | | ANK repeat and LEM domain-containing protein | | | | | | | | | | | | | |  |  |
| EFV55090.1 | | | | | | | | | | | | | | 0.002 | | | | | | | | | | | putative PDZ domain protein, partial | | | | | | | | | | | | | |  |  |
| EFV55586.1 | | | | | | | | | | | | | | 0.002 | | | | | | | | | | | T-complex protein 1 subunit alpha | | | | | | | | | | | | | |  |  |
| EFV55842.1 | | | | | | | | | | | | | | 0.002 | | | | | | | | | | | transporter, cation channel family | | | | | | | | | | | | | |  |  |
| EFV55872.1 | | | | | | | | | | | | | | 0.002 | | | | | | | | | | | ubiquitin carboxyl- hydrolase family protein | | | | | | | | | | | | | |  |  |
| EFV56183.1 | | | | | | | | | | | | | | 0.002 | | | | | | | | | | | protein OS-9 | | | | | | | | | | | | | |  |  |
| EFV56205.1 | | | | | | | | | | | | | | 0.002 | | | | | | | | | | | serine/threonine-protein kinase haspin | | | | | | | | | | | | | |  |  |
| EFV56287.1 | | | | | | | | | | | | | | 0.002 | | | | | | | | | | | hypothetical protein Tsp_06356 | | | | | | | | | | | | | |  |  |
| EFV56471.1 | | | | | | | | | | | | | | 0.002 | | | | | | | | | | | exocyst complex component 1 | | | | | | | | | | | | | |  |  |
| EFV56842.1 | | | | | | | | | | | | | | 0.002 | | | | | | | | | | | peptidase family C50 | | | | | | | | | | | | | |  |  |
| EFV56847.1 | | | | | | | | | | | | | | 0.002 | | | | | | | | | | | putative HpaII tinys locus 9c protein | | | | | | | | | | | | | |  |  |
| EFV58008.1 | | | | | | | | | | | | | | | 0.002 | | | | | | | | drug resistance transporter, EmrB/QacA family | | | | | | | | | | | | | | | |  |  |
| EFV59494.1 | | | | | | | | | | | | | | | 0.002 | | | | | | | | zinc finger protein | | | | | | | | | | | | | | | |  |  |
| EFV59704.1 | | | | | | | | | | | | | | | 0.002 | | | | | | | | retinol dehydrogenase 13 | | | | | | | | | | | | | | | |  |  |
| EFV60103.1 | | | | | | | | | | | | | | | 0.002 | | | | | | | | 60S ribosomal protein L8 | | | | | | | | | | | | | | | |  |  |
| EFV60399.1 | | | | | | | | | | | | | | | 0.002 | | | | | | | | GTP-binding protein Era | | | | | | | | | | | | | | | |  |  |
| EFV60479.1 | | | | | | | | | | | | | | | 0.002 | | | | | | | | thymidylate synthase | | | | | | | | | | | | | | | |  |  |
| EFV60590.1 | | | | | | | | | | | | | | | 0.002 | | | | | | | | conserved hypothetical protein | | | | | | | | | | | | | | | |  |  |
| EFV60905.1 | | | | | | | | | | | | | | | 0.002 | | | | | | | | phospholipase D3 protein | | | | | | | | | | | | | | | |  |  |
| EFV62419.1 | | | | | | | | | | | | | | | 0.002 | | | | | | | | putative cubilin, partial | | | | | | | | | | | | | | | |  |  |
| EFV48137.1 | | | | | | | | | | | | | | | 0.003 | | | | | | | | uridine diphosphate glucose pyrophosphatase, partial | | | | | | | | | | | | | | | |  |  |
| EFV49306.1 | | | | | | | | | | | | | | | 0.003 | | | | | | | | zinc finger matrin-type protein 2 | | | | | | | | | | | | | | | |  |  |
| EFV52768.1 | | | | | | | | | | | | | | | 0.003 | | | | | | | | metallophosphoesterase 1 | | | | | | | | | | | | | | | |  |  |
| EFV54473.1 | | | | | | | | | | | | | | | 0.003 | | | | | | | | membralin | | | | | | | | | | | | | | | |  |  |
| EFV54670.1 | | | | | | | | | | | | | | | 0.003 | | | | | | | | RNA-binding protein squid | | | | | | | | | | | | | | | |  |  |
| EFV56573.1 | | | | | | | | | | | | | | | 0.003 | | | | | | | | putative HMG box | | | | | | | | | | | | | | | |  |  |
| EFV57233.1 | | | | | | | | | | | | | | | 0.003 | | | | | | | | protein WBSCR14-like protein | | | | | | | | | | | | | | | |  |  |
| EFV57745.1 | | | | | | | | | | | | | | | 0.003 | | | | | | | | putative cytochrome c-type heme lyase | | | | | | | | | | | | | | | |  |  |
| EFV58646.1 | | | | | | | | | | | | | | | 0.003 | | | | | | | | DNA-directed RNA polymerases I, II, and III subunit RPABC3 | | | | | | | | | | | | | | | |  |  |
| EFV59707.1 | | | | | | | | | | | | | | | 0.003 | | | | | | | | suppressor of SWI4 1-like protein | | | | | | | | | | | | | | | |  |  |
| EFV59717.1 | | | | | | | | | | | | | | | 0.003 | | | | | | | | transmembrane protein 151B | | | | | | | | | | | | | | | |  |  |
| EFV60368.1 | | | | | | | | | | | | | | | 0.003 | | | | | | | | proliferating cell nuclear antigen | | | | | | | | | | | | | | | |  |  |
| EFV60868.1 | | | | | | | | | | | | | | | 0.003 | | | | | | | | conserved hypothetical protein | | | | | | | | | | | | | | | |  |  |
| EFV60927.1 | | | | | | | | | | | | | | | 0.003 | | | | | | | | pyruvate dehydrogenase complex, E1 component, pyruvate  dehydrogenase, beta subunit | | | | | | | | | | | | | | | |  |  |
| EFV61392.1 | | | | | | | | | | | | | | | 0.003 | | | | | | | | putative transketolase, thiamine diphosphate binding domain protein | | | | | | | | | | | | | | | |  |  |
| EFV62058.1 | | | | | | | | | | | | | | | 0.003 | | | | | | | | sterol O-acyltransferase 2 | | | | | | | | | | | | | | | |  |  |
| EFV53549.1 | | | | | | | | | | | | | | | 0.004 | | | | | | | | exonuclease superfamily | | | | | | | | | | | | | | | |  |  |
| EFV54343.1 | | | | | | | | | | | | | | | 0.004 | | | | | | | | putative puromycin-sensitive aminopeptidase | | | | | | | | | | | | | | | |  |  |
| EFV54641.1 | | | | | | | | | | | | | | | 0.004 | | | | | | | | N-acetyltransferase | | | | | | | | | | | | | | | |  |  |
| EFV55016.1 | | | | | | | | | | | | | | | 0.004 | | | | | | | | T-complex protein 1 subunit delta | | | | | | | | | | | | | | | |  |  |
| EFV56175.1 | | | | | | | | | | | | | | | 0.004 | | | | | | | | mitotic checkpoint protein BUB3 | | | | | | | | | | | | | | | |  |  |
| EFV58032.1 | | | | | | | | | | | | | | | 0.004 | | | | | | | | serine/threonine- protein phosphatase regulatory subunit A alpha | | | | | | | | | | | | | | | |  |  |
| EFV58393.1 | | | | | | | | | | | | | | | 0.004 | | | | | | | | conserved hypothetical protein | | | | | | | | | | | | | | | |  |  |
| EFV58473.1 | | | | | | | | | | | | | | | 0.004 | | | | | | | | eukaryotic translation initiation factor 3 subunit D | | | | | | | | | | | | | | | |  |  |
| EFV47939.1 | | | | | | | | | | | | | | | 0.005 | | | | | | | | ribonucleo protein | | | | | | | | | | | | | | | |  |  |
| EFV50493.1 | | | | | | | | | | | | | | | 0.005 | | | | | | | | fibroblast growth factor 18 | | | | | | | | | | | | | | | |  |  |
| EFV51508.1 | | | | | | | | | | | | | | | 0.005 | | | | | | | | sodium/potassium-transporting ATPase subunit beta-1-interacting protein 3 | | | | | | | | | | | | | | | |  |  |
| EFV52103.1 | | | | | | | | | | | | | | | 0.005 | | | | | | | | putative DnaJ domain protein | | | | | | | | | | | | | | | |  |  |
| EFV52155.1 | | | | | | | | | | | | | | | | 0.005 | | | | | | ATP-dependent Clp protease, proteolytic subunit ClpP | | | | | | | | | | | | | | | | |  |  |
| EFV52284.1 | | | | | | | | | | | | | | | | 0.005 | | | | | | electron transfer flavoprotein, alpha subunit | | | | | | | | | | | | | | | | |  |  |
| EFV52642.1 | | | | | | | | | | | | | | | | 0.005 | | | | | | putative C2 domain protein | | | | | | | | | | | | | | | | |  |  |
| EFV52895.1 | | | | | | | | | | | | | | | | 0.005 | | | | | | hypothetical protein Tsp_09417 | | | | | | | | | | | | | | | | |  |  |
| EFV53960.1 | | | | | | | | | | | | | | | | 0.005 | | | | | | uracil-DNA glycosylase | | | | | | | | | | | | | | | | |  |  |
| EFV54137.1 | | | | | | | | | | | | | | | | 0.005 | | | | | | sarcoglycan complex subunit protein | | | | | | | | | | | | | | | | |  |  |
| EFV56685.1 | | | | | | | | | | | | | | | | 0.005 | | | | | | eukaryotic translation initiation factor 3 subunit B | | | | | | | | | | | | | | | | |  |  |
| EFV56960.1 | | | | | | | | | | | | | | | | 0.005 | | | | | | glycosyl hydrolase family 47 | | | | | | | | | | | | | | | | |  |  |
| EFV57197.1 | | | | | | | | | | | | | | | | 0.005 | | | | | | DNA polymerase kappa | | | | | | | | | | | | | | | | |  |  |
| EFV57656.1 | | | | | | | | | | | | | | | | 0.005 | | | | | | 60S ribosomal protein L6 | | | | | | | | | | | | | | | | |  |  |
| EFV58184.1 | | | | | | | | | | | | | | | | 0.005 | | | | | | leucyl-tRNA synthetase, cytoplasmic | | | | | | | | | | | | | | | | |  |  |
| EFV58350.1 | | | | | | | | | | | | | | | | 0.005 | | | | | | conserved hypothetical protein | | | | | | | | | | | | | | | | |  |  |
| EFV58820.1 | | | | | | | | | | | | | | | | 0.005 | | | | | | Exo70 exocyst complex subunit family protein | | | | | | | | | | | | | | | | |  |  |
| EFV59699.1 | | | | | | | | | | | | | | | | 0.005 | | | | | | GDP-L-fucose synthetase | | | | | | | | | | | | | | | | |  |  |
| EFV62096.1 | | | | | | | | | | | | | | | | 0.005 | | | | | | dolichyl pyrophosphate Man9GlcNAc2 alpha-1,3-glucosyltransferase | | | | | | | | | | | | | | | | |  |  |
| EFV55343.1 | | | | | | | | | | | | | | | | 0.006 | | | | | | small nuclear ribonucleoprotein E | | | | | | | | | | | | | | | | |  |  |
| EFV56134.1 | | | | | | | | | | | | | | | | 0.006 | | | | | | conserved hypothetical protein | | | | | | | | | | | | | | | | |  |  |
| EFV56408.1 | | | | | | | | | | | | | | | | 0.006 | | | | | | putative transcription initiation factor TFIID subunit 3 | | | | | | | | | | | | | | | | |  |  |
| EFV57430.1 | | | | | | | | | | | | | | | | 0.006 | | | | | | RNA dependent RNA polymerase family protein | | | | | | | | | | | | | | | | |  |  |
| EFV59695.1 | | | | | | | | | | | | | | | | 0.006 | | | | | | voltage gated calcium channel IQ domain protein | | | | | | | | | | | | | | | | |  |  |
| EFV59762.1 | | | | | | | | | | | | | | | | 0.006 | | | | | | arsenical pump-driving ATPase | | | | | | | | | | | | | | | | |  |  |
| EFV60168.1 | | | | | | | | | | | | | | | | 0.006 | | | | | | putative tRNA pseudouridine synthase 2 | | | | | | | | | | | | | | | | |  |  |
| EFV60530.1 | | | | | | | | | | | | | | | | 0.006 | | | | | | proteasome subunit beta type-2 | | | | | | | | | | | | | | | | |  |  |
| EFV62005.1 | | | | | | | | | | | | | | | | 0.006 | | | | | | conserved hypothetical protein | | | | | | | | | | | | | | | | |  |  |
| EFV52248.1 | | | | | | | | | | | | | | | | 0.007 | | | | | | putative cysteine rich repeat-containing domain protein | | | | | | | | | | | | | | | | |  |  |
| EFV53588.1 | | | | | | | | | | | | | | | | 0.007 | | | | | | brix domain-containing protein 1 | | | | | | | | | | | | | | | | |  |  |
| EFV53939.1 | | | | | | | | | | | | | | | | 0.007 | | | | | | hypothetical protein Tsp_08607 | | | | | | | | | | | | | | | | |  |  |
| EFV54606.1 | | | | | | | | | | | | | | | | 0.007 | | | | | | putative HEAT repeat-containing domain protein | | | | | | | | | | | | | | | | |  |  |
| EFV56116.1 | | | | | | | | | | | | | | | | 0.007 | | | | | | histone acetyltransferase type B catalytic subunit | | | | | | | | | | | | | | | | |  |  |
| EFV56492.1 | | | | | | | | | | | | | | | | 0.007 | | | | | | conserved hypothetical protein | | | | | | | | | | | | | | | | |  |  |
| EFV57490.1 | | | | | | | | | | | | | | | | 0.007 | | | | | | nucleolar complex-associated protein | | | | | | | | | | | | | | | | |  |  |
| EFV57539.1 | | | | | | | | | | | | | | | | 0.007 | | | | | | angiotensin-converting enzyme, testis-specific isoform | | | | | | | | | | | | | | | | |  |  |
| EFV59475.1 | | | | | | | | | | | | | | | | 0.007 | | | | | | putative surfeit locus protein 6-like protein | | | | | | | | | | | | | | | | |  |  |
| EFV62228.1 | | | | | | | | | | | | | | | | 0.007 | | | | | | putative tetratricopeptide repeat-containing domain protein | | | | | | | | | | | | | | | | |  |  |
| EFV62503.1 | | | | | | | | | | | | | | | | 0.007 | | | | | | histone H4 | | | | | | | | | | | | | | | | |  |  |
| EFV50310.1 | | | | | | | | | | | | | | | | 0.008 | | | | | | putative Fe-S protein assembly co-chaperone HscB | | | | | | | | | | | | | | | | |  |  |
| EFV50384.1 | | | | | | | | | | | | | | | | 0.008 | | | | | | pyridoxamine 5'-phosphate oxidase | | | | | | | | | | | | | | | | |  |  |
| EFV51273.1 | | | | | | | | | | | | | | | | 0.008 | | | | | | suppressor of G2 allele of SKP1-like protein | | | | | | | | | | | | | | | | | | |
| EFV56123.1 | | | | | | | | | | | | | | | | 0.008 | | | | | | fatty acid synthase | | | | | | | | | | | | | | | | | | |
| EFV56787.1 | | | | | | | | | | | | | | | | 0.008 | | | | | | hypothetical protein Tsp_02070 | | | | | | | | | | | | | | | | | | |
| EFV57497.1 | | | | | | | | | | | | | | | | 0.008 | | | | | | conserved hypothetical protein | | | | | | | | | | | | | | | | | | |
| EFV60563.1 | | | | | | | | | | | | | | | | 0.008 | | | | | | putative immunoglobulin I-set domain protein | | | | | | | | | | | | | | | | | | |
| EFV47704.1 | | | | | | | | | | | | | | | | 0.009 | | | | | | regulator of V-ATPase in vacuolar membrane protein 1 | | | | | | | | | | | | | | | | | | |
| EFV51048.1 | | | | | | | | | | | | | | | | 0.009 | | | | | | general transcription factor IIH subunit 2 | | | | | | | | | | | | | | | | | | |
| EFV58524.1 | | | | | | | | | | | | | | | | 0.009 | | | | | | eukaryotic peptide chain release factor subunit 1 | | | | | | | | | | | | | | | | | | |
| EFV60834.1 | | | | | | | | | | | | | | | | 0.009 | | | | | | putative MIF4G domain protein | | | | | | | | | | | | | | | | | | |
| EFV50271.1 | | | | | | | | | | | | | | | | 0.01 | | | | | | putative ERCC4 domain protein | | | | | | | | | | | | | | | | | | |
| EFV57080.1 | | | | | | | | | | | | | | | | 0.01 | | | | | | putative retinal-binding protein | | | | | | | | | | | | | | | | | | |
| EFV58167.1 | | | | | | | | | | | | | | | | 0.01 | | | | | | putative signal peptidase I | | | | | | | | | | | | | | | | | | |
| EFV61962.1 | | | | | | | | | | | | | | | | 0.01 | | | | | | putative peptidase dimerization domain protein | | | | | | | | | | | | | | | | | | |
